# Fe_3_O_4_@nano-almondshell/Si(CH_2_)_3_/2-(1-piperazinyl)ethylamine as an effective magnetite almond shell-based nanocatalyst for the synthesis of dihydropyrano[3,2-*c*]chromene and tetrahydrobenzo[*b*]pyran derivatives

**DOI:** 10.1038/s41598-023-33286-w

**Published:** 2023-04-19

**Authors:** Dina Mallah, Bi Bi Fatemeh Mirjalili, Abdolhamid Bamoniri

**Affiliations:** 1 Department of Chemistry, College of Science, Yazd University, P.O. Box 89195-741, Yazd, Islamic Republic of Iran; 2 Department of Organic Chemistry, Faculty of Chemistry, University of Kashan, Kashan, Islamic Republic of Iran

**Keywords:** Catalysis, Organic chemistry

## Abstract

The preparation and design of nano-catalysts based on magnetic biopolymers as green and biocompatible nano-catalysts have made many advances. This paper deals with the preparation of magnetite biopolymer-based Brønsted base nano-catalyst from a nano-almond (*Prunus dulcis*) shell. This magnetite biopolymer-based nano-catalyst was obtained through a simple process based on the core-shelling of nano-almond shell and Fe_3_O_4_ NPs and then the immobilization of 3-chloropropyltrimethoxysilane as linker and 2-aminoethylpiperazine as a basic section. Structural and morphological analysis of this magnetite biopolymer-based nano-catalyst were done using Fourier transform infrared spectroscopy, field emission scanning electron microscopy, X-ray diffraction, Thermogravimetric analysis, Vibrating sample magnetization, Energy-dispersive X-ray spectroscopy, Brunauer–Emmett–Teller, and Transmission electron microscopy techniques. The performance of the synthesized Fe_3_O_4_@nano-almondshell/Si(CH_2_)_3_/2-(1-piperazinyl)ethylamine as a novel magnetite biopolymer-based nano-catalyst for the synthesis of dihydropyrano[3,2-*c*]chromene and tetrahydrobenzo[*b*]pyran was investigated and showed excellent efficiency.

## Introduction

In recent years, extensive research has been conducted on new polymer-based nano-catalysts. New polymers known as biopolymers will be synthesized from non-edible and highly available plants as well as agricultural and industrial wastes^1–3^. In addition, some biopolymers can be obtained from renewable sources. These biopolymers include polysaccharides (cellulose, dextrin, chitosan, etc.), protein polymers (gluten, ovalbumin, soy protein, collagen, etc.), bacterial protein (3-hydroxybutyrate), and other polymers^4^. Among these biopolymers, cellulose, and derivatives become important due to their high flexibility, abundance, chemical inertness, high strength, and ability to modify surface chemistry^5–8^. The almond (Prunus dulcis) shell is a highly efficient biomass shell and is generally disposed of or incinerated as waste, which causes environmental pollution^9^. Almond shells make up about 35–75% of the total weight of the fruit. This volume of the shell has a high practical potential that has attracted a lot of attention in recent years^10,11^. Senturk et al. used the almond shell as an adsorbent to remove rhodamine dye from aqueous solutions^12^. Mohan et al. have prepared magnetically activated carbon from almond shells to remove 2,4,6-trinitrophenol from water^13^. Cellulose is one of the main components of the almond shell, which turns this waste into suitable material for preparing nano-catalysts^14,15^.

Benzopyran or chromene is an organic bicyclic heterocyclic compound consisting of benzene and pyran rings^16,17^. Chromene derivatives have various biological and medicinal properties and therapeutic applications that have been considered by pharmaceutical and organic chemists^18^. Chromenes have shown a variety of biological properties such as antimicrobial^19^, antibacterial^20^, anticancer^21^, anti-HIV^22^, and sex pheromone^23^. Therefore, due to the biological and therapeutic properties and the great importance of chromenes, several pathways for the synthesis of these compounds have been reported, including one-step or multi-step methods^24^. One of the best attractive methods for the synthesis of chromenes is based on multi-component reactions (MCRs)^25,26^. Multicomponent reactions are one of the most successful methods in the field of increasing structural diversity and molecular complexity using a simple process. This method, as a developing process for the preparation of organic compounds, allows the development of many chemical compounds, with more structural diversity. Also, these reactions are considered a useful and effective tool for the synthesis of organic compounds and generally show good selectivity along with the reduction of by-products compared to the classical step-by-step preparation^27,28^. Higher efficiency, simplicity, saving time, and materials are some of the advantages of this category of reaction^29^. Dihydropyrano[3,2-*c*]chromene and tetrahydrobenzo[*b*]pyran are heterocyclic organic compounds containing oxygen and are very attractive. For this reason, so far, many catalysts including ZnO NPs^30^, t-ZrO_2_ NPs^31^, SB-DABCO@eosin^32^, Fe_3_O_4_@GO-NH_2_^33^, [PEMIM][OH]^34^, [(EMIM)Ac]^35^, L-Proline^36^, Chitosan-ZnO^37^, CESA^38^, Glycine^39^, rGO@Fe_3_O_4_@ZrCp_2_Cl_2_^40^, Fe_3_O_4_@*G.tea*/Cu^41^, etc. have been used for the synthesis of this class of compounds.

**Figure 1:**
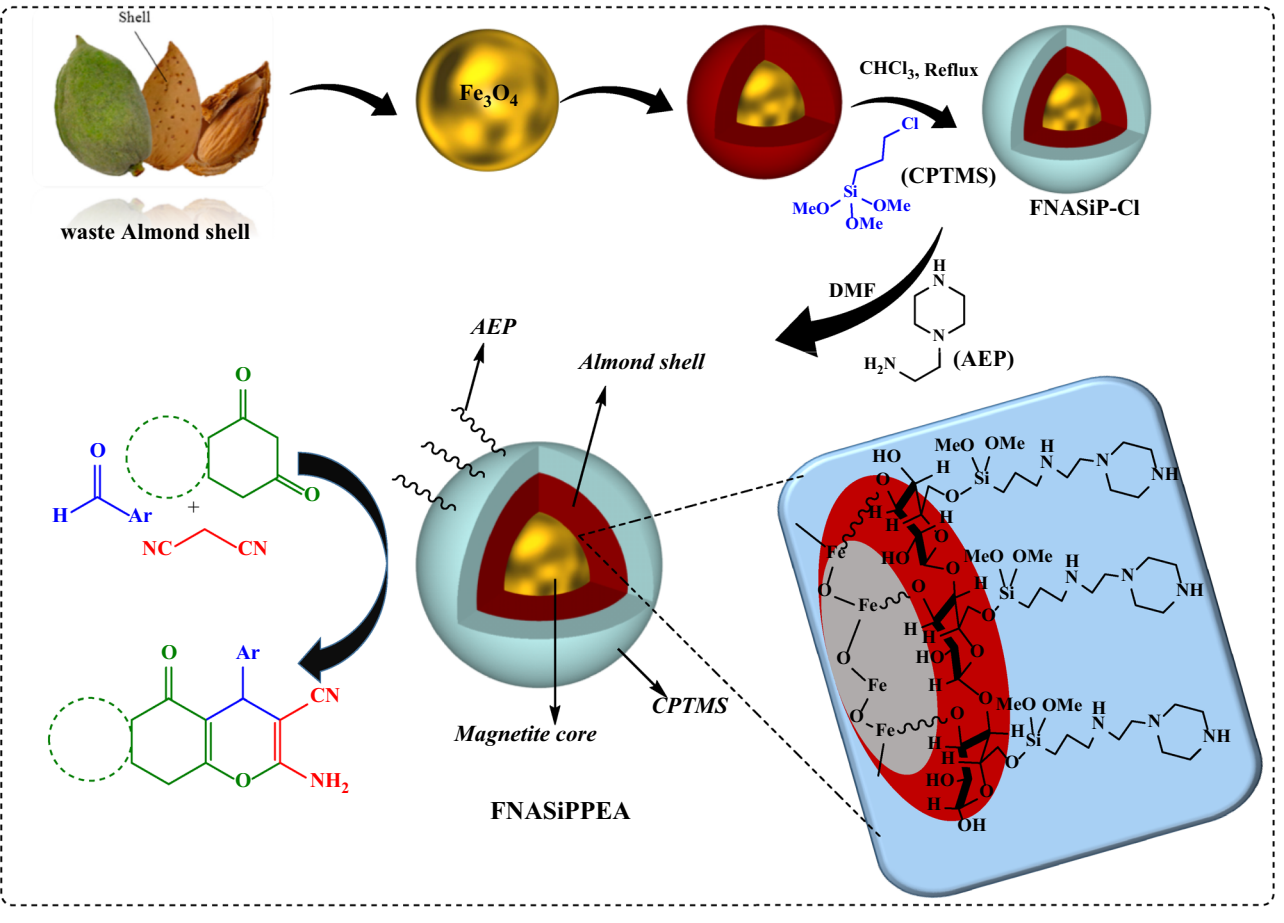
Schematic representation of FNASiPPEA, dihydropyrano[3,2-*c*]chromene, and tetrahydrobenzo[*b*]pyran.

In this work, Fe_3_O_4_@nano-almondshell/Si(CH_2_)_3_/2-(1-piperazinyl)ethylamine, abbreviated FNASiPPEA, as magnetite almond shell-based nano-catalyst was prepared and identified using FT-IR, FESEM, XRD, TGA, VSM, EDS-map, BET, TEM techniques, and then use it in the synthesis of the dihydropyrano[3,2-*c*]chromene and tetrahydrobenzo[*b*]pyran under optimized conditions (Fig. 1).

## Results and discussions

In this paper, the FNASiPPEA, a magnetite biopolymer-based nano-catalyst, was used as an environmentally friendly basic nano-catalyst for the synthesis of dihydropyrano[3,2-*c*]chromene (DHPC) and tetrahydrobenzo[*b*]pyran (THBP) derivatives through a multicomponent reaction under optimized conditions. The FNASiPPEA was first prepared by preparing Fe_3_O_4_@nanoalmondshell according to previously reported methods^42^. After that, FNASiPPEA was prepared by immobilization of 3-chloropropyltrimethoxysilane (CPTMS) and finally 2-aminoethylpiperazine (AEP) (as a base agent) on the surface of the nano-catalyst.

### FT-IR of FNASiPPEA

**Figure 2:**
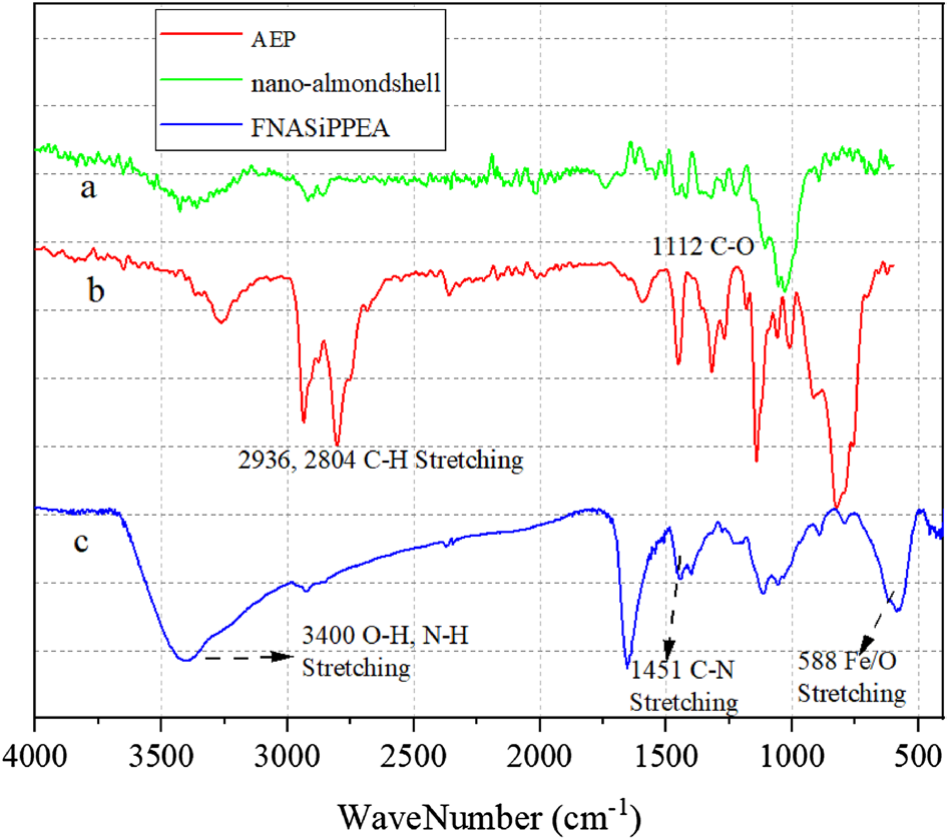
FT-IR spectra of (**a**) nano-almond shell, (**b**) AEP, and (**c**) FNASiPPEA.

FT-IR spectra of Fe_3_O_4_@nanoalmondshell, AEP, and FNASiPPEA are shown in Fig. 2. FT-IR spectrum of nano-almondshell (Fig. 2a) shows distinct peaks at 3428 cm^-1^, 2920 cm^-1^, and 1122 cm^-1^, which are related to O–H, C–H, and C–O vibrational stretching, respectively. In the FNASiPPEA spectrum (Fig. 2c), a distinct peak at 588 cm^-1^ is attributed to the Fe–O stretching vibration. Also, the broad peak at the range of 3400 cm^-1^ is attributed to the stretching vibration of N–H, which overlaps with the stretching vibration of the O–H group. CPTMS immobilization on Fe_3_O_4_@nanoalmondshell is confirmed by a characteristic peak at 1111 cm^-1^, which corresponds to the Si–O stretching vibration. The characteristic peak at 1451 cm^-1^ is related to C–N stretching vibration.

### FESEM and TEM of FNASiPPEA

**Figure 3:**
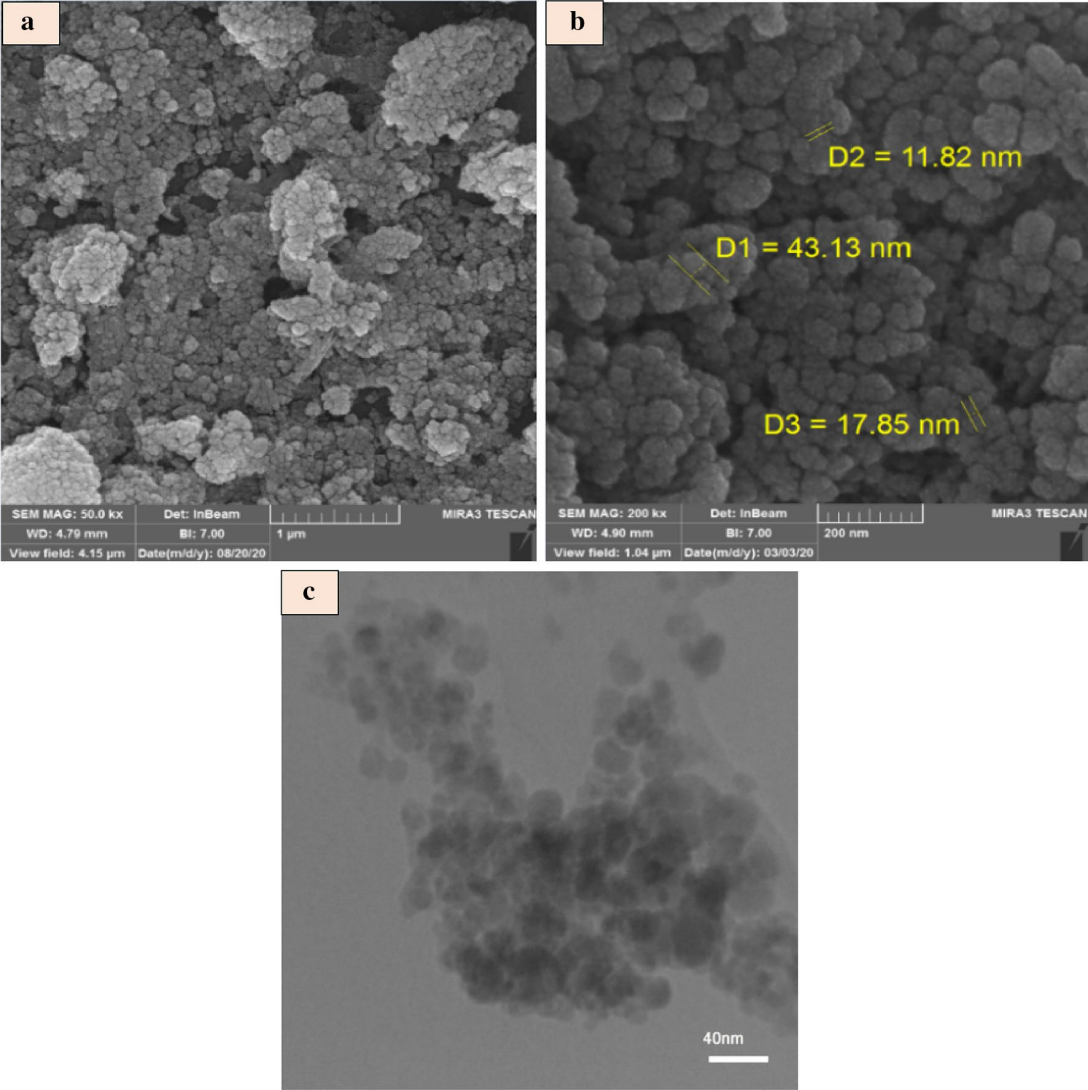
FESEM images of FNASiPPEA (**a** and **b**), and (**c**) TEM of FNASiPPEA.

The surface morphology and detailed structure of the FNASiPPEA nano-catalyst were investigated using FESEM (Fig. 3). Figures 3a and b show the average particle size of the catalyst (11–43 nm) which appeared as nanospheres with pseudo-spherical morphology. The intrinsic structure was characterized using TEM measurements (Fig. 3c) which show core–shell nanoparticles.

### PXRD (Powder X-ray diffraction) of FNASiPPEA

**Figure 4:**
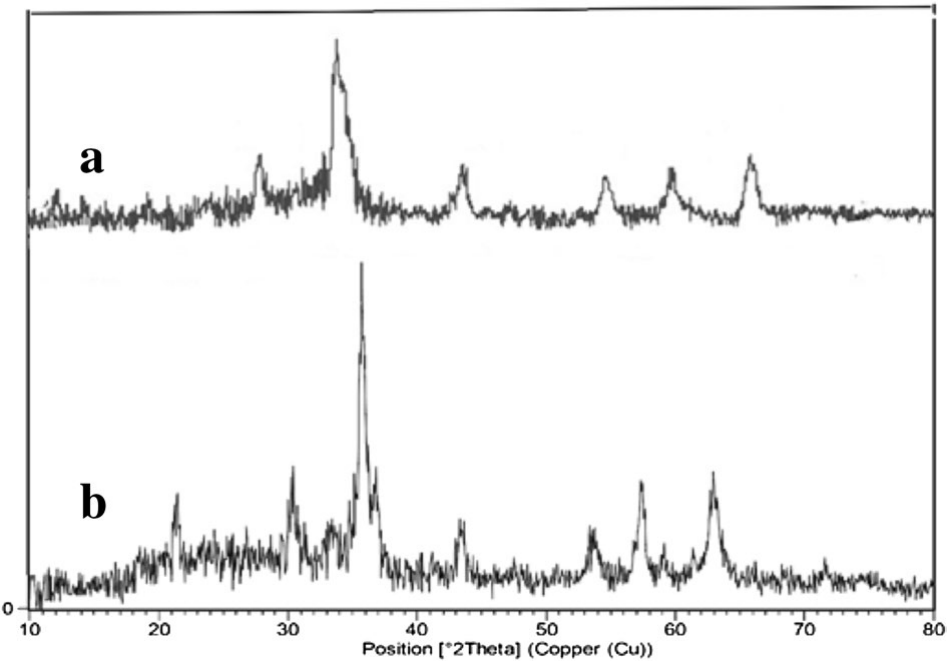
XRD patterns of (**a**) Fe_3_O_4_ NPs, and (**b**) FNASiPPEA.

Figure 4 shows the XRD patterns of Fe_3_O_4_ NPs and magnetite biopolymer-based FNASiPPEA nanoparticles. All the diffraction peaks appearing at 2*θ* = 31°, 35°, 43°, 54°, 57°, and 63° in the spectrum (4a) can be indexed as centered cubic Fe_3_O_4_, which agrees well with the reported data correspond^43^. In the XRD pattern (4b), a new peak appears at 2*θ* = 23° and a broad peak at 2*θ* = 20–30°, which is due to the presence of nano-almondshell and amorphous silica, respectively.

### TGA of FNASiPPEA

**Figure 5:**
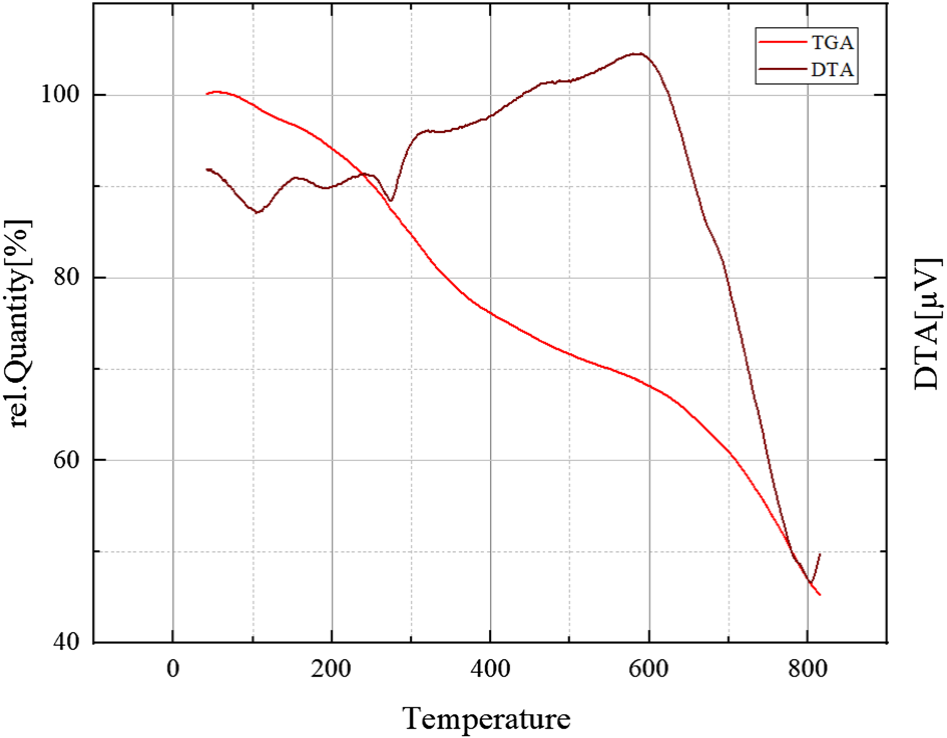
TGA/DTA curves of FNASiPPEA.

Figure 5 shows the TGA and DTA curves of magnetite FNASiPPEA. The nano-catalyst shows a small initial mass reduction at a temperature lower than 100 °C due to the removal of absorbed water and other organic solvents. At temperatures higher than 100 °C, (180–370 °C) the highest weight loss is observed in the TGA curve, which was probably due to the decomposition of nano-almondshell and organic parts (amine groups and methoxy groups) from the catalyst.

### VSM of FNASiPPEA

**Figure 6:**
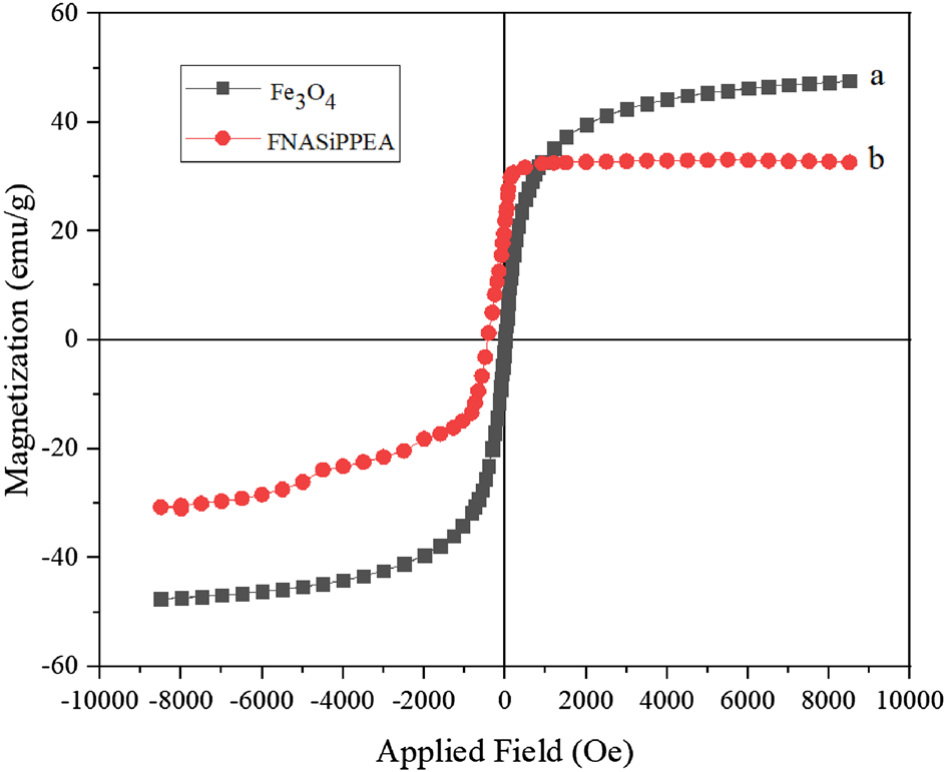
VSM analysis of (**a**) Fe_3_O_4_ NPs, (**b**) FNASiPPEA.

The magnetic properties of the FNASiPPEA were evaluated in Fig. 6. The magnetic curve shows no remanence and coercivity, indicating the nanocatalyst’s superparamagnetic behavior. The saturation magnetization value of FNASiPPEA (33 emu/g) is lower than that of Fe_3_O_4_ (47 emu/g). The low magnetization of the catalyst is attributed to the non-magnetic functionalized nano-almond shell coating on Fe_3_O_4_ NPs. However, the magnetic susceptibility of FNASiPPEA is strong enough to be separable by an external magnet from the reaction medium.

### EDX and EDS-map of FNASiPPEA

**Figure 7:**
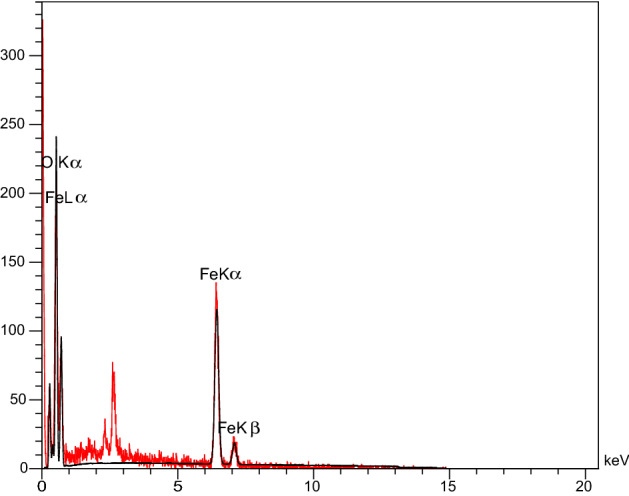
EDS diagram of FNASiPPEA.

**Figure 8:**
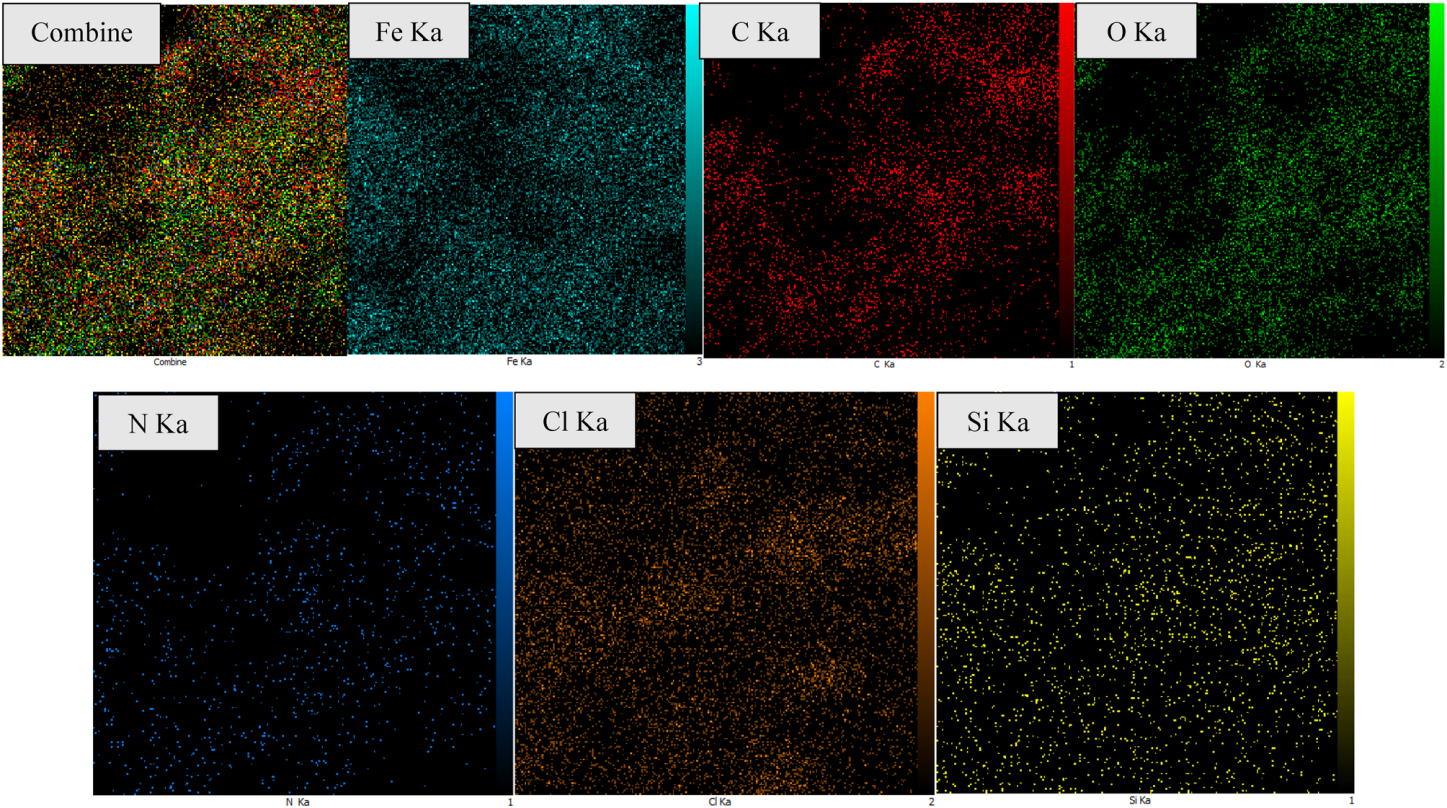
Maps of FNASiPPEA.

The elemental composition of the FNASiPPEA nano-catalyst was determined by EDX. As shown in Fig. 7, Fe, O, C, Si, and N signals are related to respectively, Fe_3_O_4_, and functionalized nano-almondshell, which appears in the EDX spectrum. The percentage composition of Fe, C, O, N, Cl, and Si elements is 30.39, 25.50, 15.39, 19.55, 9.08, and 0.48% respectively. According to the results of the EDS-mapping analysis Fig. 8, the distribution of these elements is homogeneously on the surface of the nano-catalyst.

### BET of FNASiPPEA

**Figure 9:**
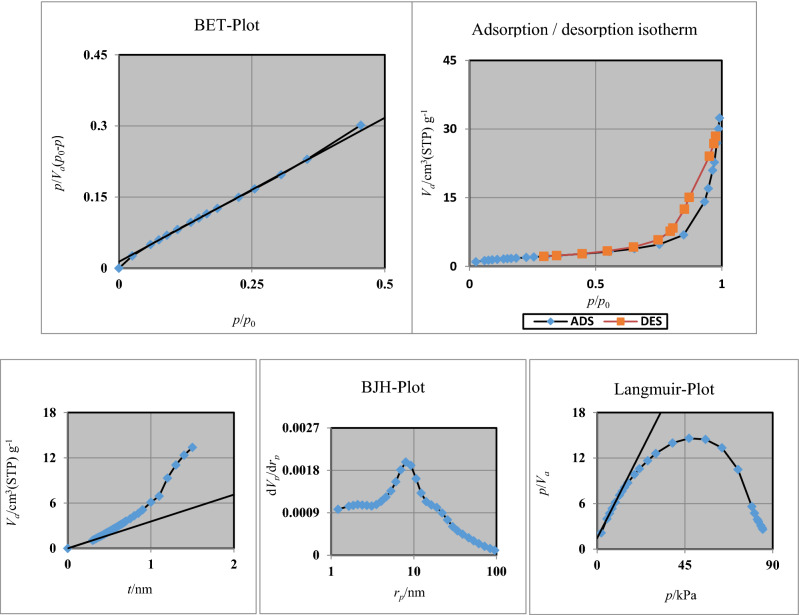
N_2_ adsorption (blue line)—desorption (red line) isotherm and corresponding diagrams pore size distributions (BJH, BET, Langmuir, *t*-plot).

The BET (Brunauer–Emmett–Teller) surface area of the prepared nano-catalyst was obtained by nitrogen adsorption and desorption measurements (Fig. 9). The N_2_ isotherms related to the type IV isotherm in the IUPAC classification have shown H_3_ type rings, which can indicate the existence of mesopores and also have non-hard pores. As shown in Table 1 a_s_, BJH (Barrett–Joyner–Halenda), and pore diameter were 7.0116 m^2^ g^-1^, 0.050029 cm^3^ g^-1^, and 28.206 nm respectively.

**Table 1:** Parameters obtained from porosity analysis.

BET	
*V*_*m*_	1.611 [cm^3^ (STP) g^−1^]
a_s,BET_	7.0116 [m^2^ g^−1^]
*C*	45.307
Total pore volume(*p*/*p*_0_ = 0.990)	0.049443 [cm^3^ g^−1^]
Mean pore diameter	28.206 [nm]
Langmuir plot	
Vm	1.9705[cm^3^ (STP) g^−1^]
a_s,Lang_	7.0116 [m^2^ g^−1^]
B	0.3498
t plot	
Plot data	Adsorption branch
a_1_	5.4877 [m^2^ g^−1^]
V_1_	0 [cm^3^ g^−1^]
BJH plot	
Plot data	Adsorption branch
V_p_	0.050029 [cm^3^ g^−1^]
*r*_*p,peak*_(*Area*)	7.99 [nm]
a_p_	9.0803 [m^2^ g^−1^]

All the above results confirm the successful synthesis of magnetite biopolymer-based FNASiPPEA. After the detailed description of the prepared nano-catalyst, its catalytic performance was investigated for DHPC synthesis. Therefore, different reaction conditions such as the amount of catalyst, solvent, and temperature were investigated for a model reaction between 4-nitrobenzaldehyde, 4-hydroxycoumarin, and malononitrile (Table 2). While screening the model reaction in different solvents such as H_2_O, EtOH, and H_2_O/EtOH, the best result was obtained in the EtOH solvent (Table 2, entry 10).

**Table 2:** Optimization of reaction conditions for the synthesis of DHPC.^a^


Entry	Conditions		
	Solvent/ Temp. (℃)/ Catalyst (g)	Time (min)	Yield (%)^b^
1	-/ r. t./ FNASiPPEA (0.005)	240	80
2	-/ r. t./ FNASiPPEA ( 0.01)	150	83
3	-/ r. t./ FNASiPPEA (0.015)	100	82
4	-/ r. t./ FNASiPPEA (0.02)	80	86
5	-/ 35/ FNASiPPEA (0.02)	80	85
6	-/ 45/ FNASiPPEA (0.02)	45	87
7	-/ 55/ FNASiPPEA (0.02)	40	86
8	-/ 65/ FNASiPPEA (0.02)	30	87
9	-/ 80/ FNASiPPEA (0.02)	30	89
10	EtOH/ 80/ FNASiPPEA (0.02)	10	95
11	EtOH: H_2_O (1:1)/ 80/ FNASiPPEA (0.02)	25	95
12	H_2_O/ 80/ FNASiPPEA (0.02)	30	90

^a^Conditions: 4-nitrobenzaldehyde (1 mmol), 4-hydroxycoumarin (1 mmol), and malononitrile (1.5 mmol), Solvent (10 ml). ^b^Isolated Yield.

After optimizing the reaction conditions, to determine the application range of FNASiPPEA, various aldehydes were used in the reaction. The results are summarized in Table 3.

**Table 3:** FNASiPPEA catalyzed the synthesis of DHPC.^a^


Entry	R	Time (min)	Yield (%)^b^	m. p. (refs.)
1	4-NO_2_–C_6_H_4_–	5	95	259–261 ^44^
2	4-F–C_6_H_4_–	7	96	261–263 ^45^
3	4-Br–C_6_H_4_–	5	96	248–250 ^44^
4	4-OH–C_6_H_4_–	10	94	260–262 ^44^
5	4-OMe-C_6_H_4_-	15	94	248–251 ^46^
6	4-isopropyl–C_6_H_4_–	25	90	250–252 ^45^
7	3-NO_2_–C_6_H_4_–	30	93	261–263 ^44^
8	2-NO_2_–C_6_H_4_–	30	95	259–261 ^44^
9	2-Cl–C_6_H_4_–	35	95	273–275 ^45^
10	2,4-(Cl)_2_–C_6_H_3_–	10	98	259–261 ^46^
11	2,6-(Cl)_2_–C_6_H_3_–	20	90	273–276 ^47^
12	C_6_H_5_–	20	92	255–257 ^46^
13	3-pyridine–	5	96	250–252
14	Pentyl–	25	80	188–190 ^48^

^a^Conditions: aldehyde (1 mmol), 4-hydroxycoumarin (1 mmol), malononitrile (1.5 mmol), 80 °C, EtOH Solvent, catalyst (0.02 g) ^b^Isolated yield.

Then, optimization of the reaction conditions for the synthesis of THBP was carried out. Hence, the reaction between 4-nitrobenzaldehyde, dimedone, and malononitrile in the presence of 0.02 g of catalyst at 50 °C in solvent-free conditions has been adopted (Table 4, entry 9). The results are summarized in Table 4.

**Table 4:** Optimization of the reaction conditions for the synthesis of THBP.^a^


Entry	Conditions		
	Solvent/ Temp. (℃)/ Catalyst (g)	Time (min)	Yield (%)^b^
1	-/ r. t./ FNASiPPEA ( 0.01)	110	83
2	-/ r. t./ FNASiPPEA (0.015)	88	82
3	-/ r. t. / FNASiPPEA (0.02)	75	86
4	-/ 35/ FNASiPPEA (0.02)	60	85
5	-/ 45/ FNASiPPEA (0.02)	45	87
6	-/ 50/ FNASiPPEA (0.02)	20	88
7	-/ 55/ FNASiPPEA (0.02)	30	86
8	-/ 80/ FNASiPPEA (0.02)	80	89
9	EtOH/ 50/ FNASiPPEA (0.02)	5	95
10	EtOH: H_2_O (1:1)/ 50/ FNASiPPEA (0.02)	25	95
11	H_2_O/ 50/ FNASiPPEA (0.02)	30	90

^a^conditions: 4-nitrobenzaldehyde (1 mmol), dimedone (1 mmol), malononitrile (1.5 mmol), Solvent (10 ml). ^b^Isolated Yield.

We used various aldehydes in the reaction to investigate the application range of FNASiPPEA as a magnetite biopolymer-based nano-catalyst. The result is presented in Table 5.

**Table 5:** Synthesis of THBP derivatives catalyzed by FNASiPPEA.^a^


Entry	R	Time (min)	Yield (%)^b^	m. p. (refs.)
1	4-NO_2_–C_6_H_4_–	5	95	181–217 ^49^
2	4-F–C_6_H_4_–	5	94	191–194 ^49^
3	4-Br–C_6_H_4_–	5	96	199–200 ^49^
4	4-Cl–C_6_H_4_–	7	97	215–216 ^48^
5	4-OH–C_6_H_4_–	15	94	213–215 ^51^
6	4-OMe–C_6_H_4_–	15	94	208–210 ^51^
8	3-NO_2_–C_6_H_4_–	35	93	216–218 ^51^
9	2-NO_2_–C_6_H_4_–	35	95	234–236 ^51^
10	2-Cl–C_6_H_4_–	35	95	216–218 ^50^
11	2,6-(Cl)_2_–C_6_H_3_–	15	95	249–251 ^51^
12	C_6_H_5_–	25	92	231–234 ^51^
13	Furan-	15	94	217–219 ^51^
14	Pentyl-	30	83	162–164 ^51^
15	Styryl-	25	90	217–219 ^51^

^a^Conditithons: aldehyde (1 mmol), dimedone (1 mmol), malononitrile (1.5 mmol), 50 °C, EtOH as the solvent, catalyst (0.02 g), ^b^Isolated yield.

To compare the efficiency of this magnetite biopolymer-based nano-catalyst with other catalysts for the synthesis of DHPC and THBP derivatives, a summary of the results was collected in Tables 6 and 7. As can be seen in Tables 6 and 7, the reaction efficiency of this catalyst is better than other catalysts and the reaction time is shorter than others.

**Table 6:** Comparison of FNASiPPEA with other catalysts for the synthesis of DHPC.

Entry	Conditions			
	Temp. (℃), Solvent, Catalyst	Time (min)	Yield (%)	Refs
1	70, H_2_O: EtOH, MNPs–PhSO_3_H	15	87	^52^
2	90, Solvent-free, Fe_3_O_4_@SiO_2_-Imine/Phenoxy-Cu(II)	17	95	^53^
3	80, Solvent-free, f CoFe_2_O_4_@FA-Er	26	96	^54^
4	Reflux, EtOH, Zn_3_(PO_4_) _2_.4H_2_O	30	85	^55^
5	80, EtOH, FNASiPPEA	10	95	This work

**Table 7:** Comparison activity of FNASiPPEA with another catalyst for the synthesis of THBP.

Entry	Conditions			
	Temp. (℃), Solvent, Catalyst	Time (min)	Yield (%)	Refs
1	80, EtOH, NH_4_Al(SO_4_)_2_ 12H_2_O	130	93	^56^
2	r. t., H_2_O, (S)-proline	30	82	^57^
3	50, EtOH, Hal-Py-IL	90	100	^58^
4	100, H_2_O, ChCl/urea/thiourea DES	17	90	^59^
5	50, EtOH, FNASiPPEA	5	95	This work

### Hot filtration test

**Figure 10:**
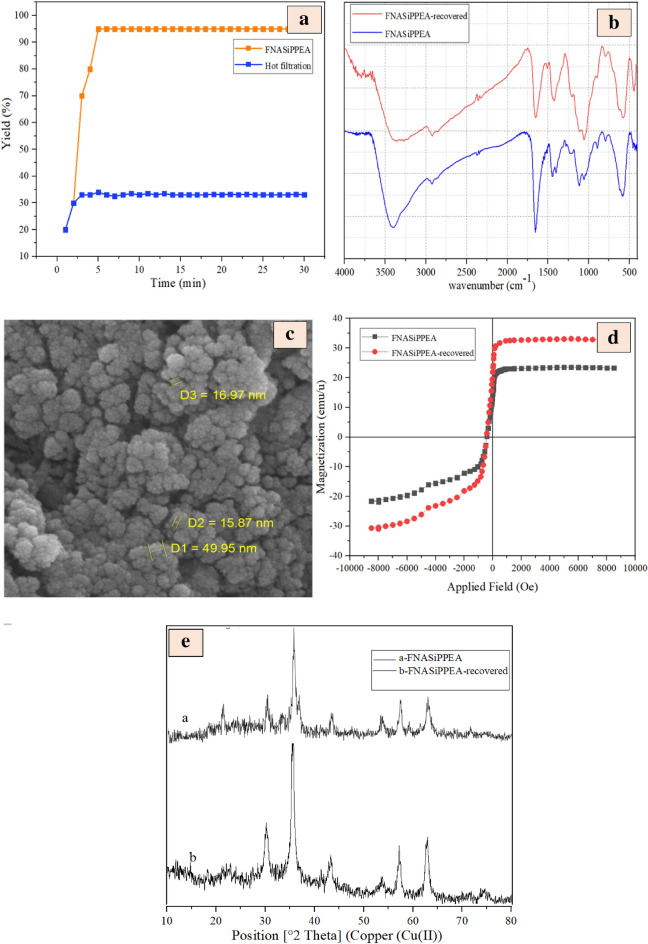
(**a**) Hot filtration test, (**b**) FT-IR, (**c**) FESEM, (**d**) VSM, and (**e**) XRD of recovered bio-based nano-catalyst.

Because the FNASiPPEA is a heterogeneous nano-catalyst, a heterogeneity test called hot filtration was performed. In this way, first, the reaction was allowed to continue in the presence of the FNASiPPEA nano-catalyst, and then after half the time, the catalyst was removed from the reaction mixture and continued the reaction, as can be seen in Fig. 10a, no reaction progress was observed in the absence of the nano-catalyst, which indicates no leakage of solid catalyst into the reaction mixture. Therefore, the FNASiPPEA nano-catalyst is heterogeneous and suitable for DHPC and THBP synthesis reactions without any leaching.

### Reusability of FNASiPPEA

**Figure 11:**
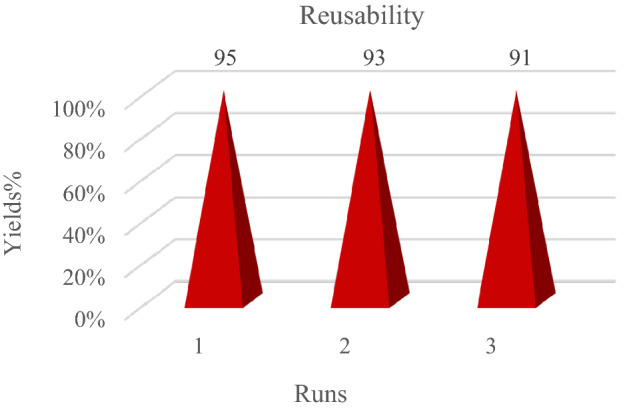
Reusability of FNASiPPEA for the synthesis of DHPC.

**Figure 12:**
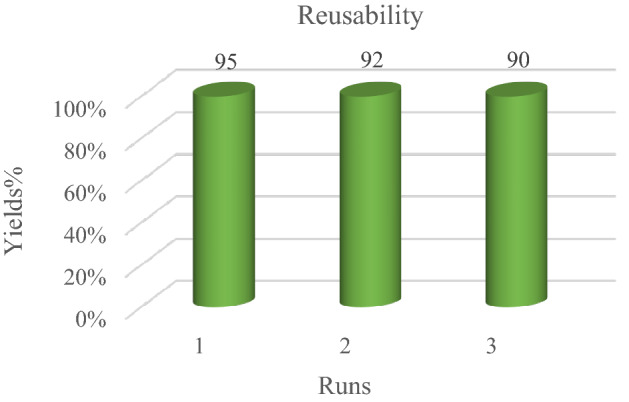
Recyclability of FNASiPPEA for the synthesis of THBP.

To check the recyclability of the catalyst, after the completion of the reaction, the catalyst can be separated from the reaction mixture with a magnet, and after washing with chloroform (CHCl_3_) and drying at the ambient temperature, and then can be reused for the synthesis of DHPC and THBP, which includes aldehyde (1 mmol), 1,3-diketone (4-hydroxycoumarin, dimedone), (1 mmol), and malononitrile (1.5 mmol) under optimized conditions. Therefore, the reusability of the catalyst for the model reaction was evaluated for the synthesis of DHPC and THBP (Figs. 11 and 12). FT-IR, XRD, VSM, and FESEM analyses of the nano-catalyst recovered after the 3rd run was also performed. According to Figs. 10b, c, d, and e, the matching of FT-IR, XRD, VSM, and SEM patterns obtained after the third run with the primary nano-catalyst confirmed the preservation of the catalyst structure.

### Proposed mechanism for synthesis of dihydropyrano[3,2-*c*]chromene and tetrahydrobenzo[*b*]pyran

**Figure 13:**
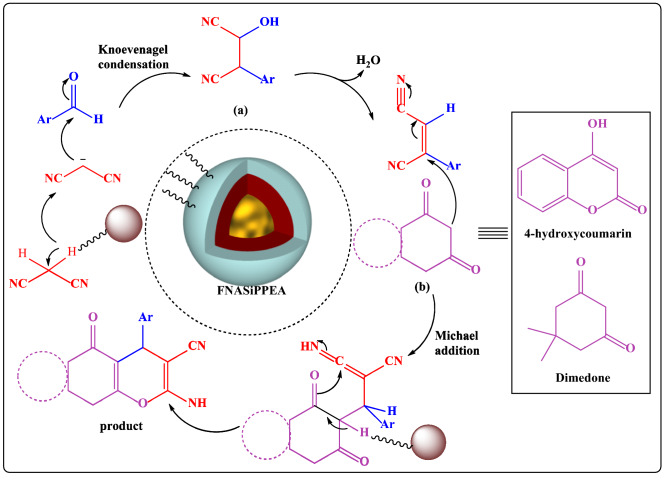
Proposed mechanism for synthesis of DHPC and THBP.

Figure 13 shows the possible mechanism for the synthesis of dihydropyrano[3,2-*c*]chromene and tetrahydrobenzo[*b*]pyran derivatives using FNASiPPEA as a magnetite Brønsted base nano-catalyst. First, the Knoevenagel condensation between malononitrile and aldehyde is followed by loss of water to form an intermediate (a). Then the Michael addition between the intermediates (a) and (b) (dimedone, 4-hydroxycoumarin) and then intramolecular cyclization and tautomerization in the presence of the catalyst leads to the production of the corresponding product.

## Experimental section

### Materials and methods

Chemicals were purchased from Merck, Fluka, and Aldrich Chemical Companies. ^1^H NMR and ^13^C NMR spectra were recorded at 400 and 100 MHz, respectively. Fourier transform infrared (FT-IR) measurements (in KBr pellets or ATR) were recorded on a Brucker spectrometer. Melting points were determined on a Büchi B-540 apparatus. The X-ray diffraction (XRD) pattern was obtained by a Philips Xpert MPD diffractometer equipped with a Cu Kα anode (*k* = 1.54 Å) in the 2*θ* range from 10° to 80°. Field Emission Scanning Electron Microscopy (FESEM) was obtained on a Mira 3-XMU. VSM measurements were performed by using a vibrating sample magnetometer (Meghnatis Daghigh Kavir Co. Kashan Kavir, Iran). Energy-dispersive X-ray spectroscopy (EDS) of nano-catalyst was measured by an EDS instrument and Phenom pro X. The EDX-MAP micrographs were obtained on the MIRA II detector SAMX (France). Thermal gravimetric analysis (TGA) was conducted using the “STA 504” instrument. Transmission electron microscopy (TEM) was obtained using a Philips CM120 with a LaB6 cathode and an accelerating voltage of 120 kV. BELSORP MINI II nitrogen adsorption apparatus (Japan) for recording Brunauer–Emmett–Teller (BET) of nano-catalyst at 77 K.

### Preparation of nano-almondshell

To prepare the nano-almondshell, the almondshell was heated in boiling water for 30 min, dried, and powdered. After that, was treated with a 17.5 w/v NaOH solution at 90 °C for 24 h under reflux conditions. Subsequently, the almondshell was filtered and washed with distilled water until the alkali was eliminated. Then, bleached with 100 mL of 1:1 aqueous dilution of 3.5% w/v sodium hypochlorite (NaOCl) at 80 °C for 3 h under reflux conditions. The resulting almondshell particles were hydrolyzed partially using the 35% sulfuric acid (H_2_SO_4_) aqueous solution with an almondshell-to-acid weight ratio of 1–10 at 45 °C. After 3 h, the obtained suspension was diluted with water five-fold to stop the hydrolysis reaction. The suspension was centrifuged at 4000 rpm to separate the nano-almondshell from the acidic medium (yield 60%).

### Preparation of Fe_3_O_4_@nano-almondshell

In a 250 mL flask, 3 g of nano-almondshell and 100 mL acetic acid (CH_3_COOH) of 0.05 M were added. After that, FeCl_3_.6H_2_O (3.51 g, 13 mmol) and FeCl_2_.4H_2_O (1.29 g, 6.5 mmol) were added and stirred for 6 h at 80 °C. Then, 8 mL of NH_4_OH (25%), was added dropwise and stirred for 45 min. The precipitated brown products were isolated from the solution by a magnet, washed 3 times with distilled water, and dried in an oven at 80 °C for 4 h. The weight of the Fe_3_O_4_@nano-almondshell obtained is 4.141 g.

### Synthesis of Fe_3_O_4_@nano-almondshell/Si(CH_2_)_3_Cl (FNASiP-Cl)

In a 100 mL flask, 1 g of dried Fe_3_O_4_@nano-almondshell was dispersed in the mixture of 10 mL of chloroform (CHCl_3_), and 3.4 mL of 3-chloropropyltrimethoxysilane was added dropwise. The mixture was sonicated at 25 °C for 20 min and then, the mixture was carried out under reflux conditions for 4 h. Finally, the result was collected using a magnet and washed three times with chloroform.

### Preparation of magnetite FNASiPPEA

The FNASiP-Cl (0.5 g) was dispersed in ethanol by ultrasonic for 20 min at room temperature and then dried. The next, 0.5 g of dried FNASiP-Cl and 2-(1-piperazinyl)ethylamine (AEP) (0.129 g, 1 mmol) was heated in 10 mL *N, N*-dimethylformamide (DMF) for 24 h at 80 °C. The resulting precipitates were cooled, washed with dichloromethane (CH_2_Cl_2_), and dried.

### General synthesis of dihydropyrano[3,2-*c*]chromene and tetrahydrobenzo[*b*]pyran derivatives

For the synthesis of dihydropyrano[3,2-*c*]chromene, in a 50 ml round bottom flask, 4-hydroxycoumarin (1 mmol, 0.162 g), 4-nitrobenzaldehyde (1 mmol, 0.151 g), malononitrile (1.5 mmol, 0.099 g), FNASiPPEA (0.02 g), and 10 ml EtOH was added. The reaction mixture was refluxed at 80 °C and stirred for appropriate periods as shown in Table 2. After the end of the reaction (TLC, n-hexane: ethyl acetate 6:4), the catalyst FNASiPPEA was separated from the reaction mixture by an external magnet, the solvent was removed under reduced pressure, and the precipitate was washed with methanol and recrystallized with chloroform for further purification.

For the synthesis of tetrahydrobenzo[*b*]pyran, the reaction of dimedone (1 mmol, 0.140 g), 4-nitrobenzaldehyde (1 mmol, 0.151 g), malononitrile (1.5 mmol, 0.099 g), and magnetite nano-catalyst FNASiPPEA (0.02 g) was carried out in 10 ml of EtOH (Table 4). After the completion of the reaction (TLC, *n*-hexane: ethyl acetate 7:3), the magnetite catalyst was removed from the reaction mixture by a magnet, the solvent was removed under reduced pressure, and the product was obtained after washing and recrystallization with chloroform.

## Conclusion

In summary, the magnetite almondshell-based nano-catalyst was prepared, characterized, and used for the synthesis of DHPC and THBP. The prepared nano-catalyst FNASiPPEA shows high catalytic activity and good reusability. Meanwhile, this method is non-toxic and biodegradable, it may be used to prepare other biopolymer-based nano-catalysts for more interesting reactions.

## Supplementary Information

Supplementary Information.

## Data Availability

The datasets generated and/or analysed during the current study are available in this article and its supplementary information files.
