# Supplementary material for: Fe3O4@nano-almondshell/Si(CH2)3/2-(1-piperazinyl)ethylamine as an effective magnetite almond shell-based nanocatalyst for the synthesis of dihydropyrano[3,2-c]chromene and tetrahydrobenzo[b]pyran derivatives
Source: Sci Rep. 2023 Apr 19;13:6376. doi: 10.1038/s41598-023-33286-w (PMC10115822; doi:10.1038/s41598-023-33286-w)
Supplement: Supplementary file 1 — Supplementary Information. [file 41598_2023_33286_MOESM1_ESM.docx]

**Fe_3_O_4_@nano-almondshell/Si(CH_2_)_3_/2-(1-piperazinyl)ethylamine as an effective magnetite almond shell-based nanocatalyst for the synthesis of dihydropyrano[3,2-*c*]chromene and**

**tetrahydrobenzo[*b*]pyran derivatives**

Dina Mallah,^1^ Bi Bi Fatemeh Mirjalili,^*1^ Abdolhamid Bamoniri^2^

^1^Department of Chemistry, College of Science, Yazd University, P.O. Box 89195-741, Yazd, I.R.IRAN. Fax: +983538210644; Tel: +983531232672. Email: [fmirjalili@yazd.ac.ir](mailto:fmirjalili@yazd.ac.ir)

^2^Department of Organic Chemistry, Faculty of Chemistry, University of Kashan, Kashan, I.R.IRAN.

**2-Amino-4-(4-nitrophenyl)-5-oxo-4,5-dihydropyrano[3,2-*c*]chromene-3-carbonitrile**

Pale yellow solid, Melting point: 259-261 ºC, FT-IR (ATR)/υ(cm^-1^): 3429, 3369, 3334 , 2195, 1716, 1672, 1603. ^1^HNMR (400MHz, DMSO-d_6_) δ( ppm): 4.67 (s, 1H, CH), 8.17 (d, 2H, *J*=8.8 Hz, Ar-H), 7.91 (d, 1H, *J*=7.2 Hz, Ar-H), 7.74 (t, 1H, *J*=7.6 Hz, Ar-H), 7.46-4.60 (m, 6H, Ar-H).

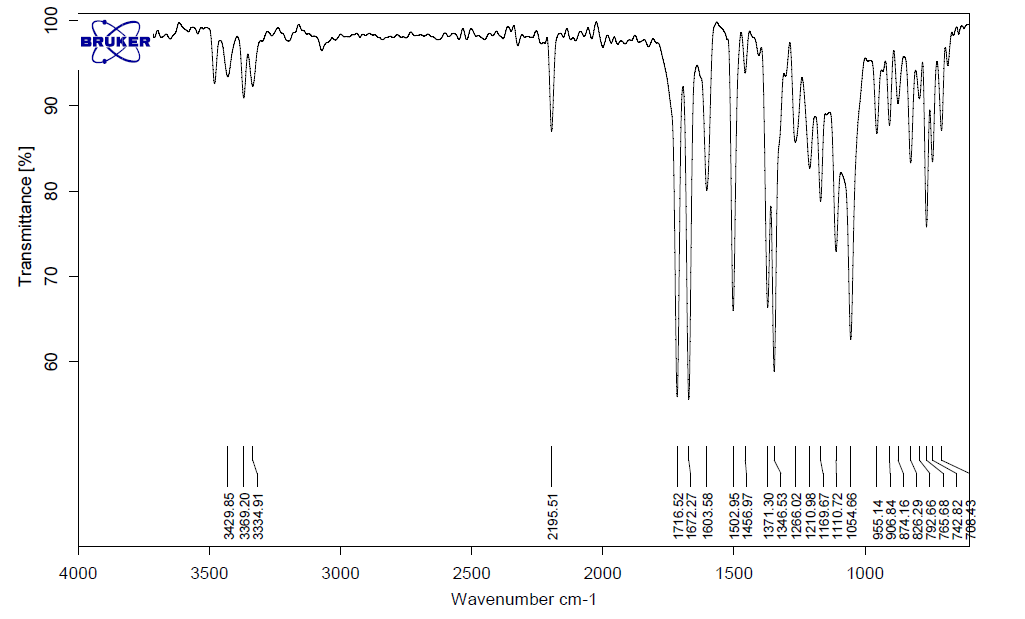


**Fig.S1. The FT-IR of** **2-Amino-4-(4-nitrophenyl)-5-oxo-4,5-dihydropyrano[3,2-*c*]chromene-3-carbonitrile**

**
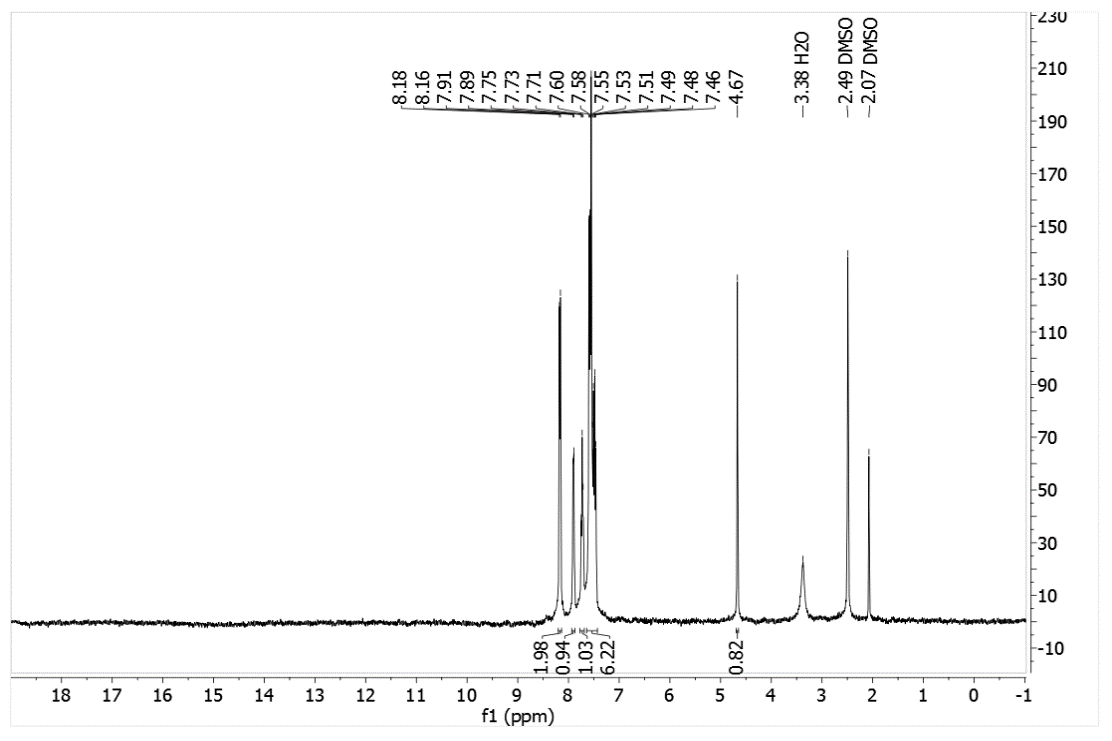
**

**Fig.S2. The ^1^H NMR spectrum of 2-Amino-4-(4-nitrophenyl)-5-oxo-4,5-dihydropyrano[3,2-*c*]chromene-3-carbonitrile**

**
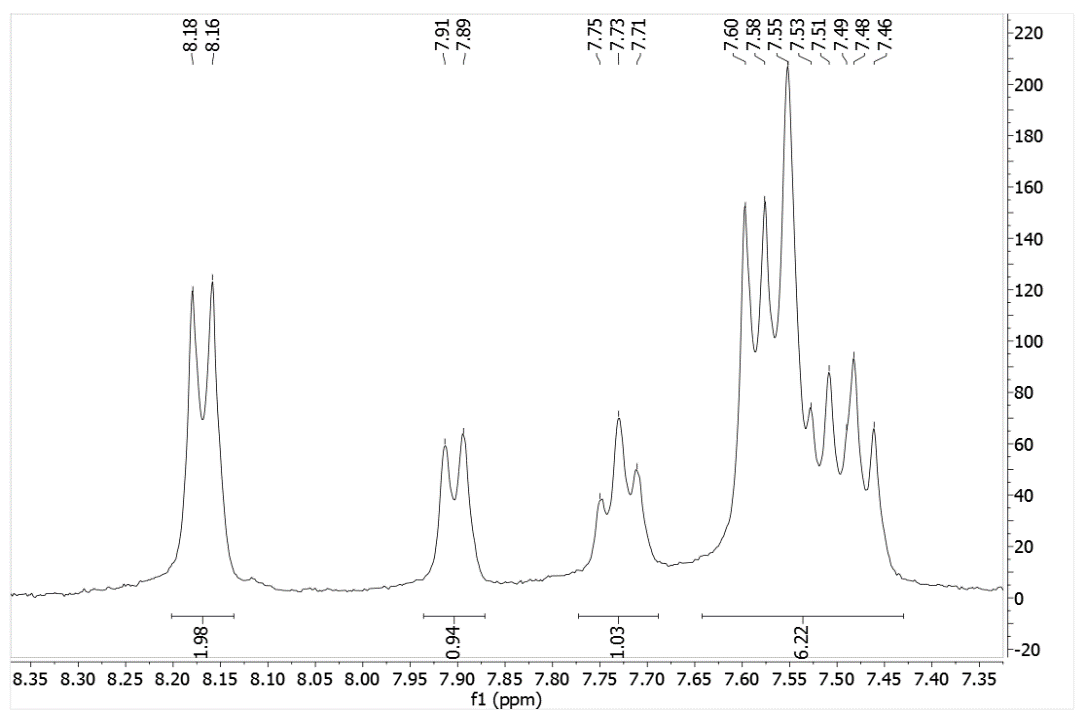
**

**Fig.S3. The ^1^H NMR spectrum of 2-Amino-4-(4-nitrophenyl)-5-oxo-4,5-dihydropyrano[3,2-*c*]chromene-3-carbonitrile**

**2-Amino-4-(4-fluorophenyl)-5-oxo-4,5-dihydropyrano[3,2-*c*]chromene-3-carbonitrile**

White Solid, Melting point: 261-263 ºC, FT-IR (ATR)/υ(cm^-1^): 3376, 3295, 3188, 2192, 1713, 1674, 1601; ^1^H NMR (400 MHz,DMSO-d_6_) δ (ppm): 4.54( 1H, s, CH), 7.17-7.21 (2H, t, *J*= 8.8Hz, Ar-H), 7.35-7.39 (2H, td, *J_1_*= 3.2Hz, *J*_2_= 6.8Hz, Ar-H), 7.49-7.57 (4H, m ), 7.75-7.80 (1H, dt, *J_1_*= 1.6Hz, J_2_= 8Hz, Ar-H),7.94-7.97 ( 1H, dd, *J*_1_= 1.6Hz, *J*_2_= 8Hz, Ar-H).

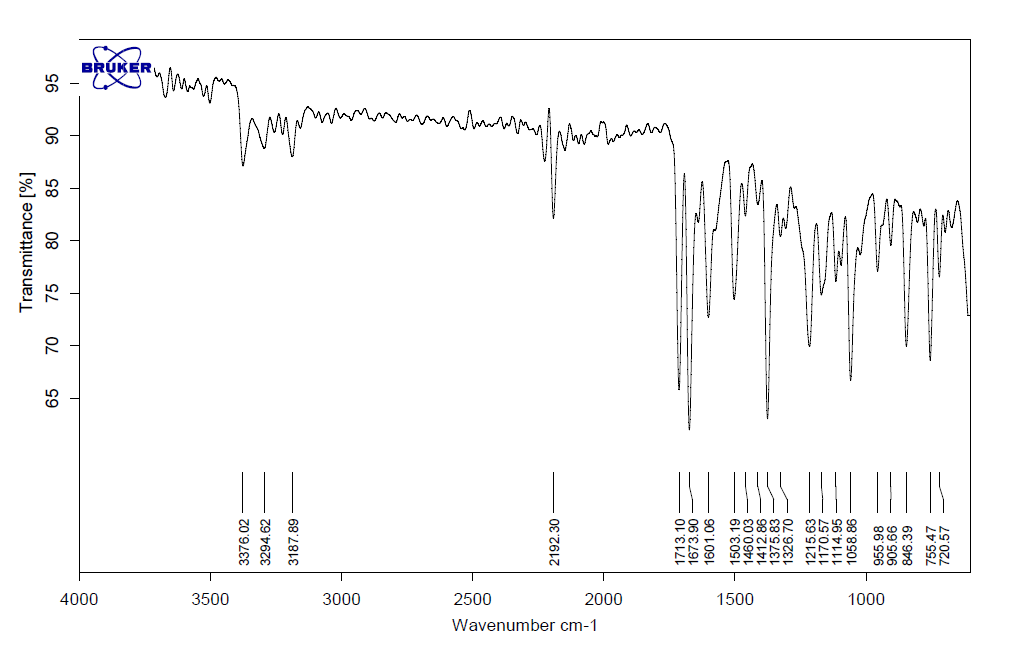


**Fig.S4. The FT-IR of 2-Amino-4-(4-fluorophenyl)-5-oxo-4,5-dihydropyrano[3,2-*c*]chromene-3-carbonitrile**


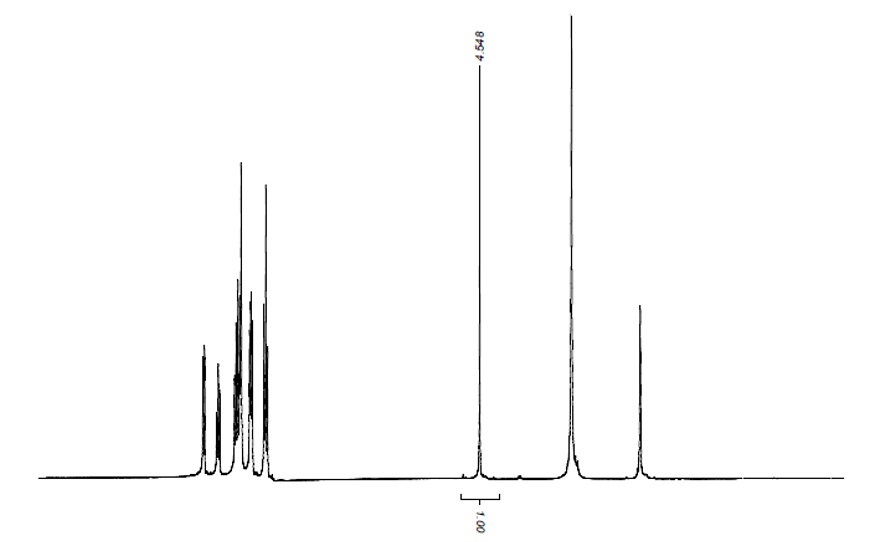


**Fig.S5. The ^1^H NMR spectrum of 2-Amino-4-(4-fluorophenyl)-5-oxo-4,5-dihydropyrano[3,2-*c*]chromene-3-carbonitrile**


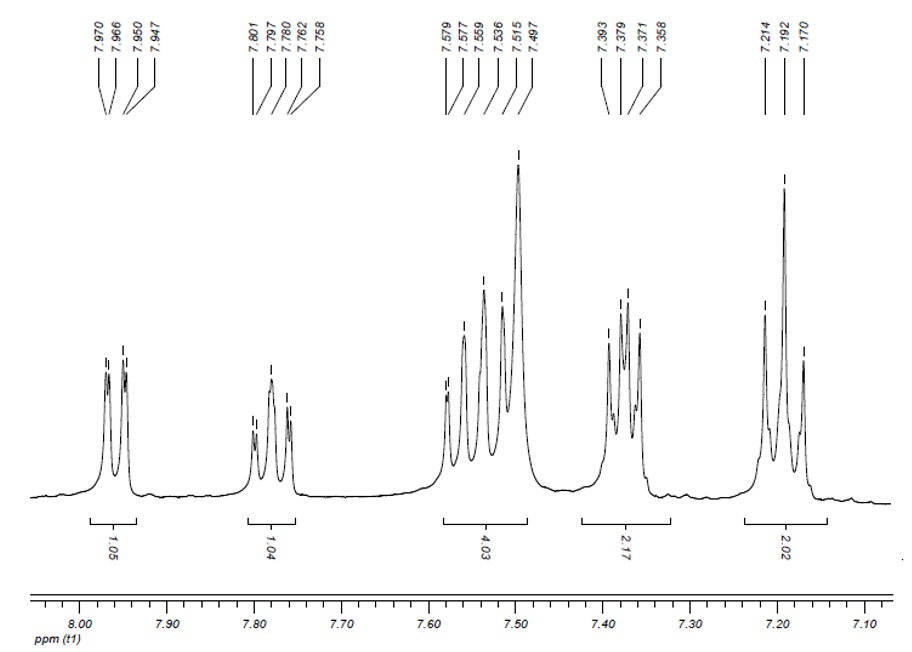


**Fig.S6. The ^1^H NMR spectrum of 2-Amino-4-(4-fluorophenyl)-5-oxo-4,5-dihydropyrano[3,2-*c*]chromene-3-carbonitrile**

**2-Amino-4-(4-bromophenyl)-5-oxo-4,5-dihydropyrano[3,2-*c*]chromene-3-carbonitrile**

White Solid, Melting point: 248-250 ºC. FT-IR (ATR)/υ(cm^-1^): 3382, 3292, 3186, 2189, 1707, 1672, 1604; ^1^HNMR (400MHz, DMSO-d_6_) δ( ppm): 4.46 (s, 1H, CH ), 7.45-7.51 (m, 6H, Ar-H), 7.73 (t, 1H, *J*=8.4 Hz, Ar-H), 7.87 (d, 2H, *J=*8 Hz, Ar-H).

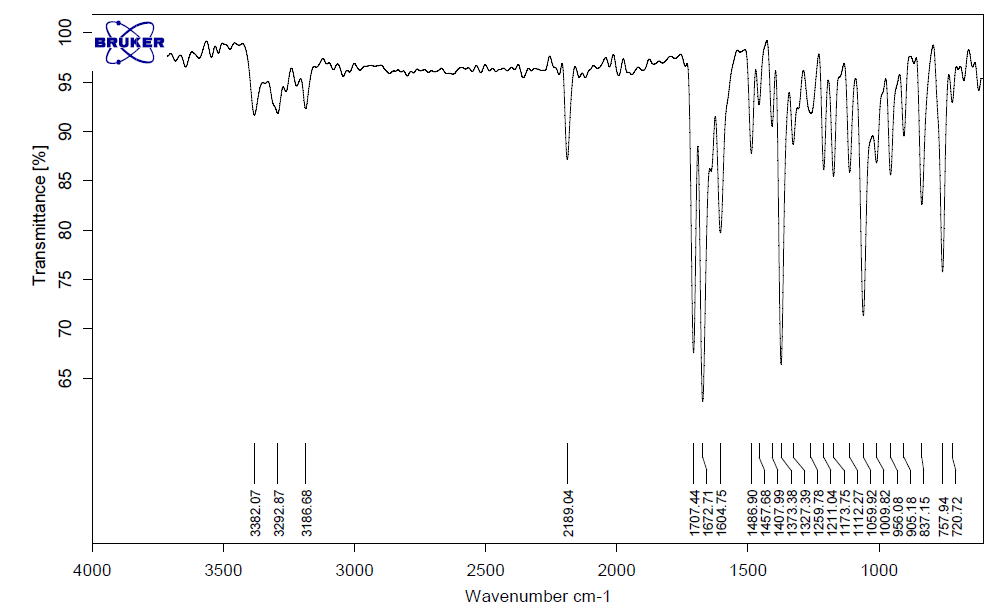


**Fig.S7. The FT-IR of 2-Amino-4-(4-bromophenyl)-5-oxo-4,5-dihydropyrano[3,2-*c*]chromene-3-carbonitrile**


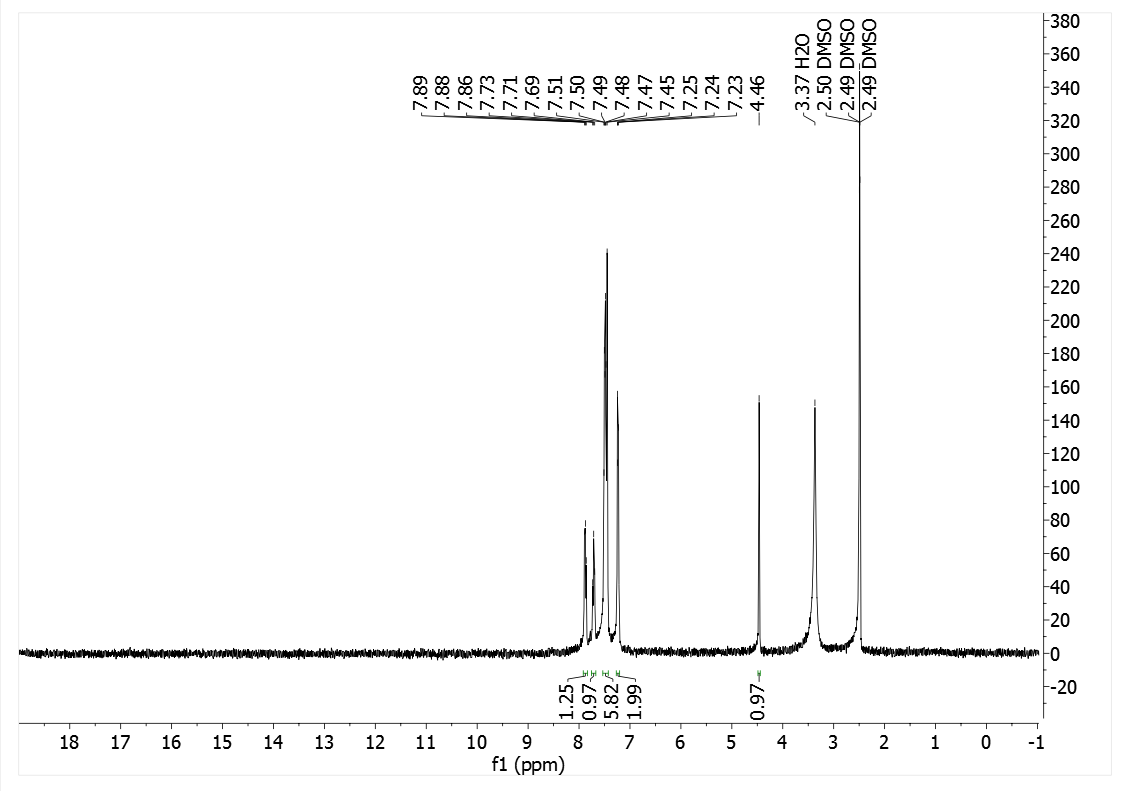


**Fig.S8. The ^1^H NMR spectrum of 2-Amino-4-(4-bromophenyl)-5-oxo-4,5-dihydropyrano[3,2-*c*]chromene-3-carbonitrile**


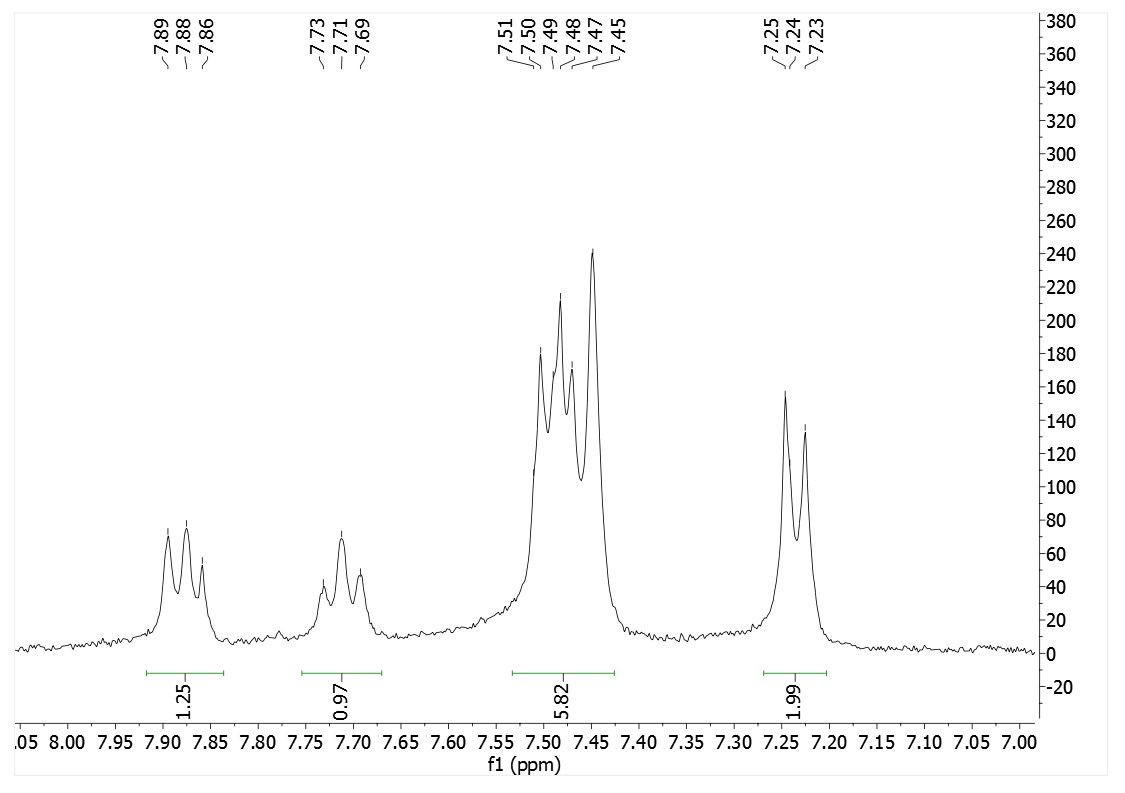


**Fig.S9. The ^1^H NMR spectrum of 2-Amino-4-(4-bromophenyl)-5-oxo-4,5-dihydropyrano[3,2-*c*]chromene-3-carbonitrile**

**2-Amino-4-(4-hydroxyphenyl)-5-oxo-4,5-dihydropyrano[3,2-*c*]chromene-3-carbonitrile**

White Solid, Melting point: 260-262 ºC, FT-IR (ATR)/υ(cm^-1^): 3399, 3285, 3180, 2195, 1692, 1670, 1601; ^1^HNMR(500MHz,DMSO-d_6_) δ (ppm): 4.33 (s, 1H), 6.70 (d, 2H, *J* = 8.5 Hz), 7.06 (d, 2H, *J* = 8.5 Hz), 7.34 (s, 2H, NH_2_), 7.42-7.48 (m, 2H), 7.69 (dt, 1H, *J*_1_ = 7.8 Hz, *J*_2_ = 1.6 Hz), 7.89 (dd, 1H, *J*_1_ = 7.8 Hz, *J*_2_ =1.4 Hz), 9.35 (s, 1H, OH). ^13^C NMR (125 MHz, DMSO-d_6_) *δ* (ppm) 58.40, 75.11, 104.50, 113.03, 114.21, 115.12, 116.63, 119.43, 122.79, 124.68, 128.73, 133.73, 152.05, 152.97, 156.49, 157.90, 159.55, 163.91.

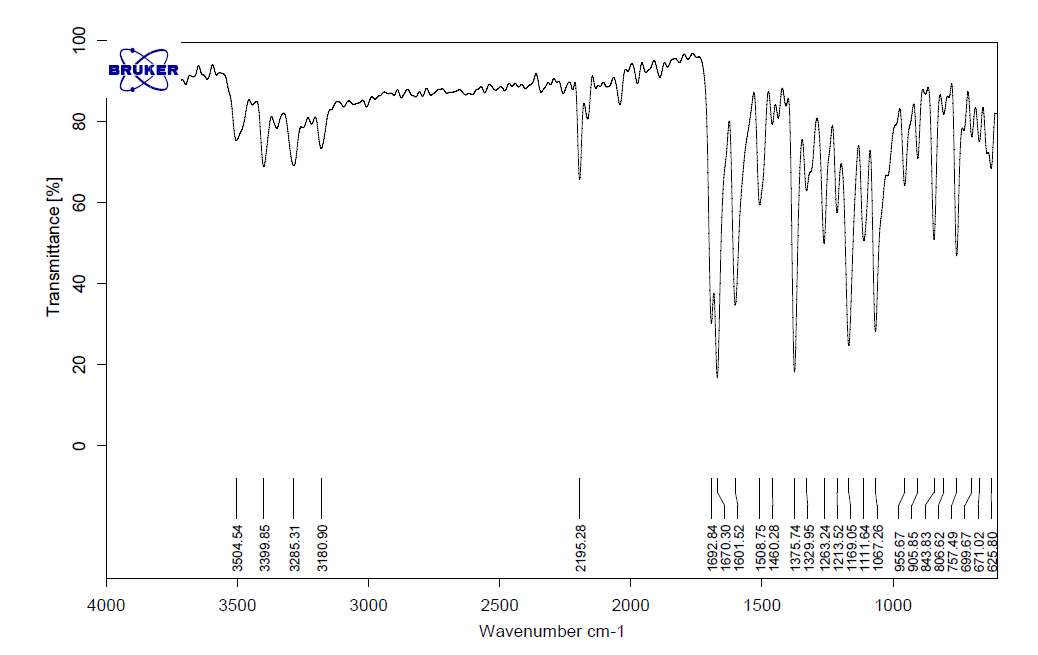


**Fig.S10. The FT-IR of 2-Amino-4-(4-hydroxyphenyl)-5-oxo-4,5-dihydropyrano[3,2-*c*]chromene-3-carbonitrile**


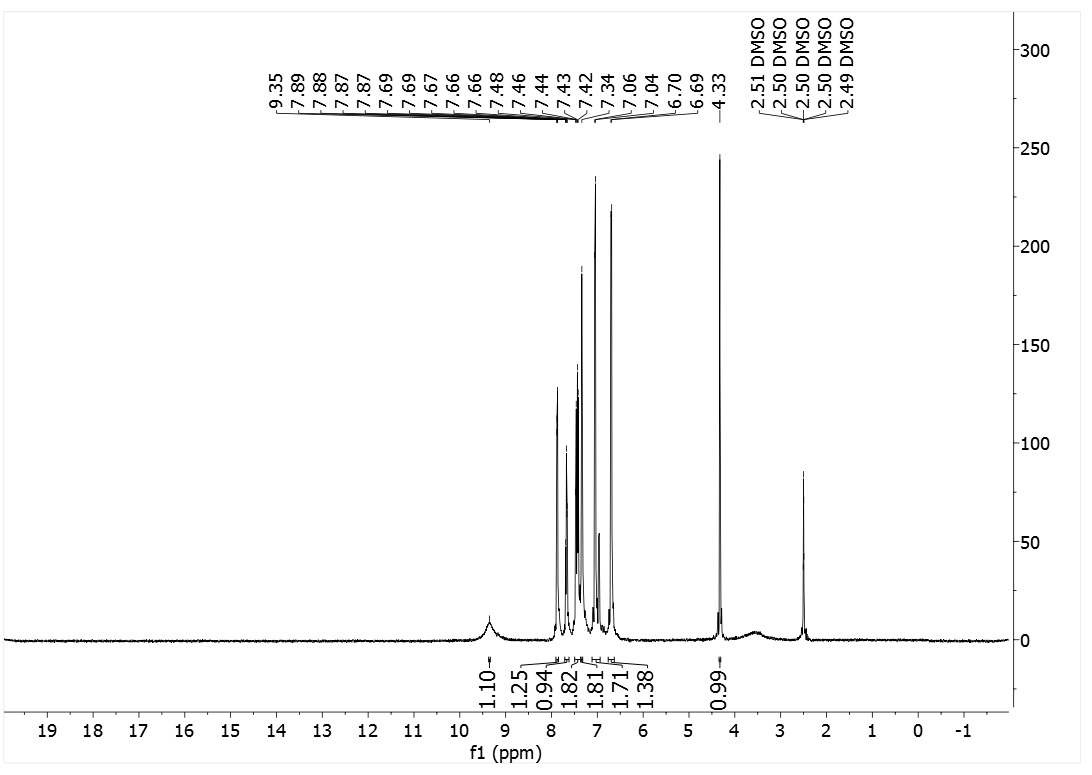


**Fig.S11. The ^1^H NMR spectrum of2-Amino-4-(4-hydroxyphenyl)-5-oxo-4,5-dihydropyrano[3,2-*c*]chromene-3-carbonitrile**


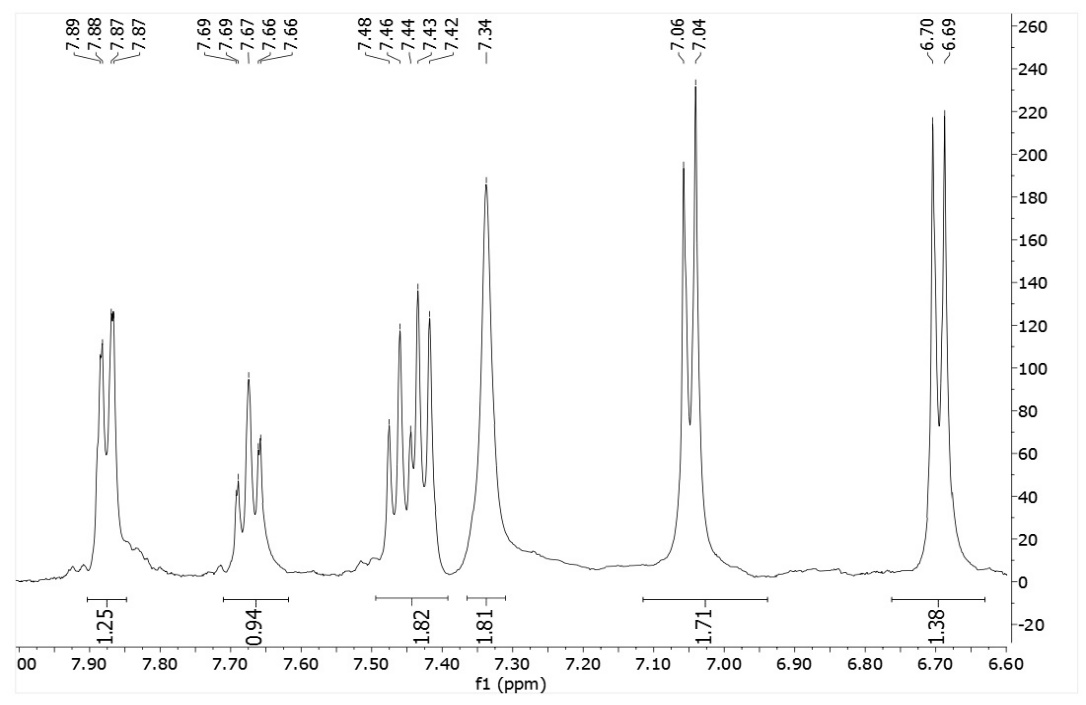


**Fig.S12. The ^1^H NMR spectrum of2-Amino-4-(4-hydroxyphenyl)-5-oxo-4,5-dihydropyrano[3,2-*c*]chromene-3-carbonitrile**


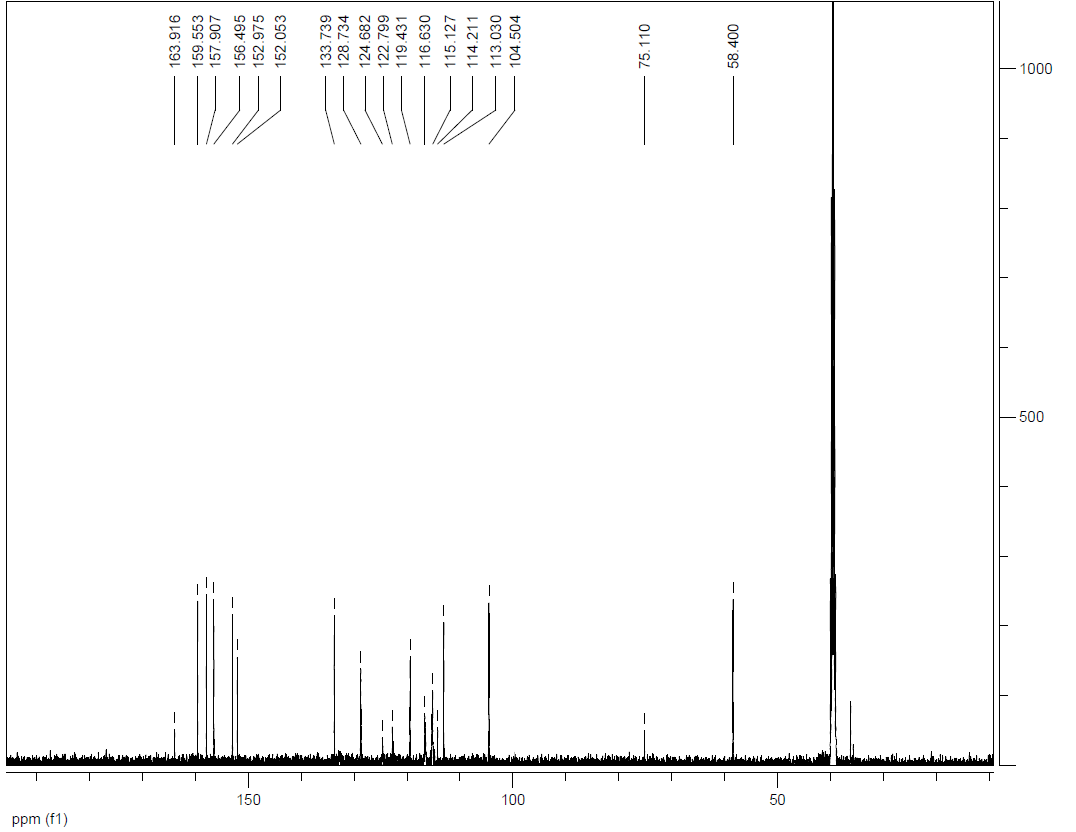


**Fig.S13. The ^1^H NMR spectrum of 2-Amino-4-(4-hydroxyphenyl)-5-oxo-4,5-dihydropyrano[3,2-*c*]chromene-3-carbonitrile**

**2-Amino-4-(4-methoxyphenyl)-5-oxo-4,5-dihydropyrano[3,2-*c*]chromene-3-carbonitrile**

Pale yellow solid, Melting point: 248-251 ºC, FT-IR (ATR)/υ(cm^-1^): 3401, 3322, 3217, 2194, 1707, 1664, 1596; ^1^HNMR(500MHz,DMSO-d_6_) δ (ppm): 3.71 (s, 3H, OCH_3_), 4.41 (s, 1H), 6.75-6.73 (dd, 1H, *J*_1_ = 8.5 Hz, *J*_2_ = 2.5 Hz, Ar-H), 6.84-6.88 (m, 2H, Ar-H), 7.37 (s, 2H, NH_2_), 7.45-7.50 (m, 2H, Ar-H), 7.72 (t, 1H, *J* = 7Hz, Ar-H), 7.88-7.90 (dd, 1H, *J*_1_ = 8 Hz, *J*_2_ =1.5 Hz, Ar-H). ^13^C NMR (125 MHz, DMSO-d_6_) *δ* (ppm) 55.49, 58.11, 66.34, 78.97, 101.95, 104.11, 106.59, 112.22, 113.05, 116.66, 119.34, 119.67, 124.69, 135.84, 147.93, 148.50, 152.11, 153.18, 157.92, 159.60.

**
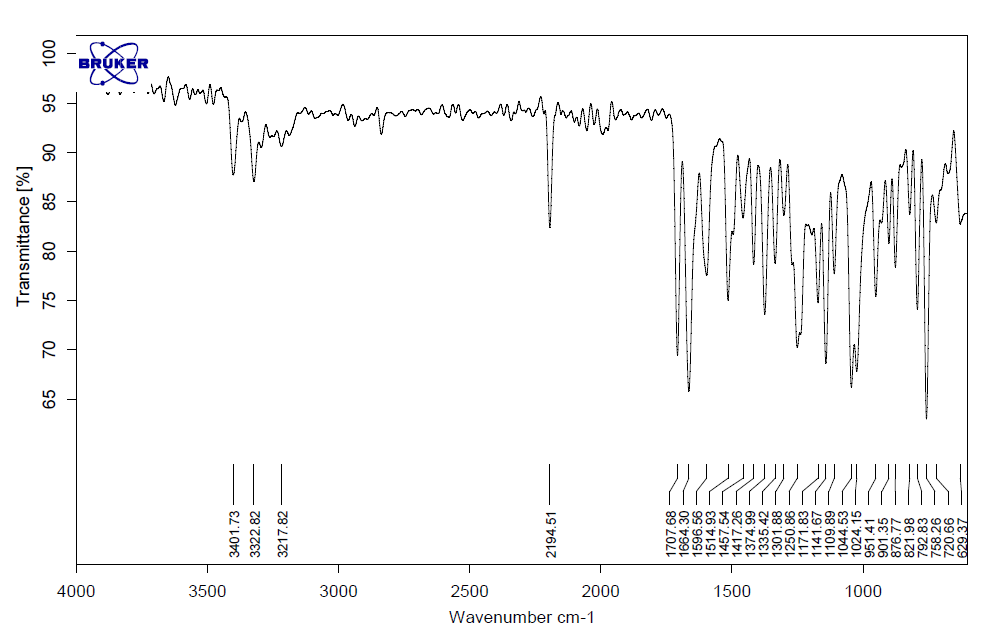
**

**Fig.S14. The FT-IR of 2-Amino-4-(4-methoxyphenyl)-5-oxo-4,5-dihydropyrano[3,2-*c*]chromene-3-carbonitrile**

**
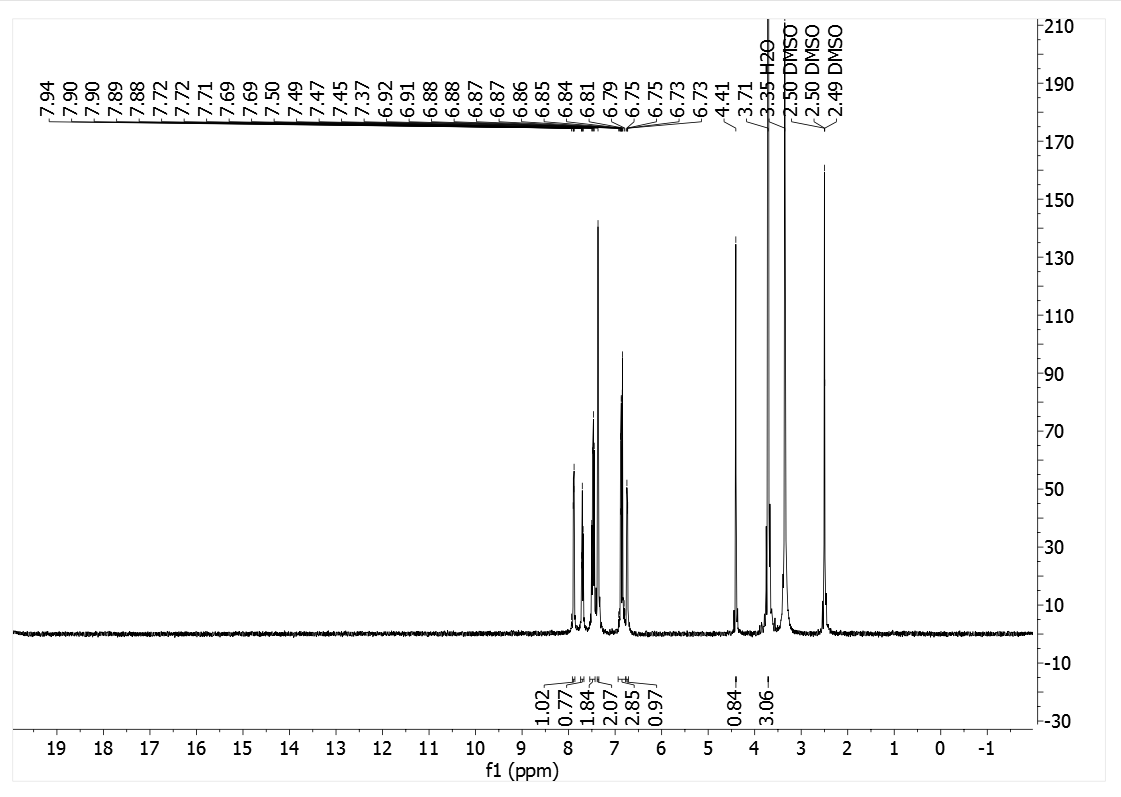
**

**Fig.S15. The ^1^H NMR spectrum of 2-Amino-4-(4-methoxyphenyl)-5-oxo-4,5-dihydropyrano[3,2-*c*]chromene-3-carbonitrile**

**
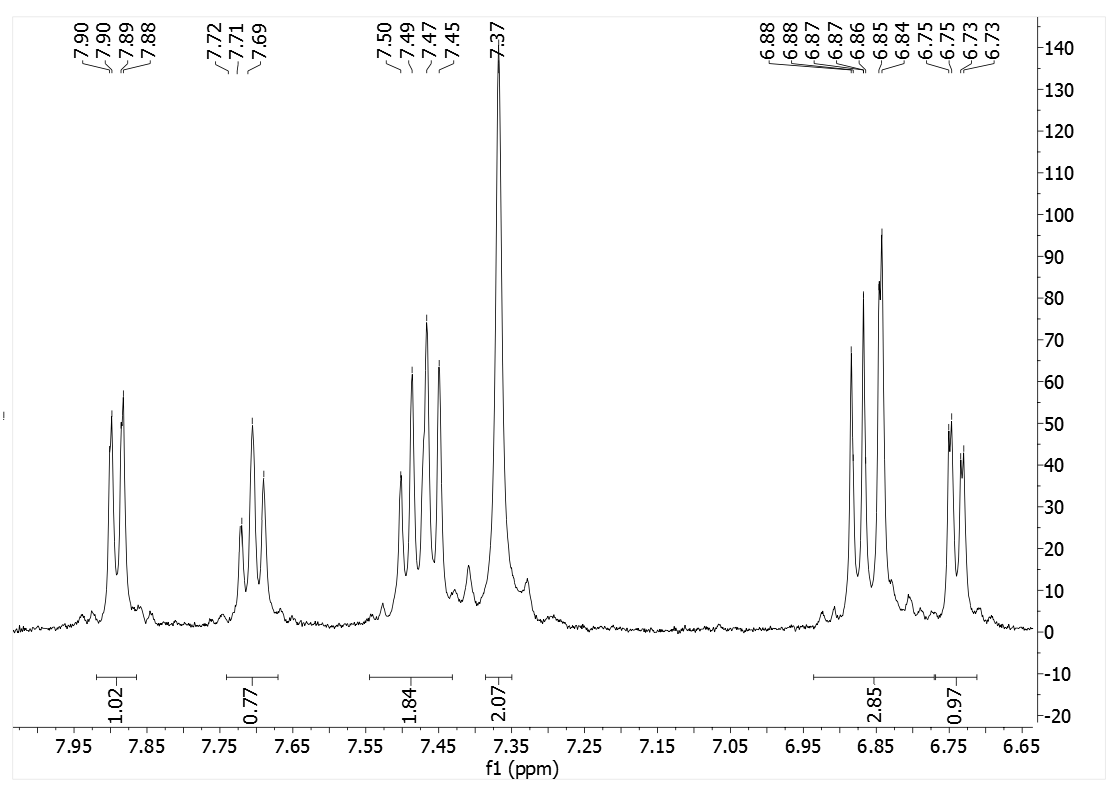
**

**Fig.S16. The ^1^H NMR spectrum of 2-Amino-4-(4-methoxyphenyl)-5-oxo-4,5-dihydropyrano[3,2-*c*]chromene-3-carbonitrile**

**
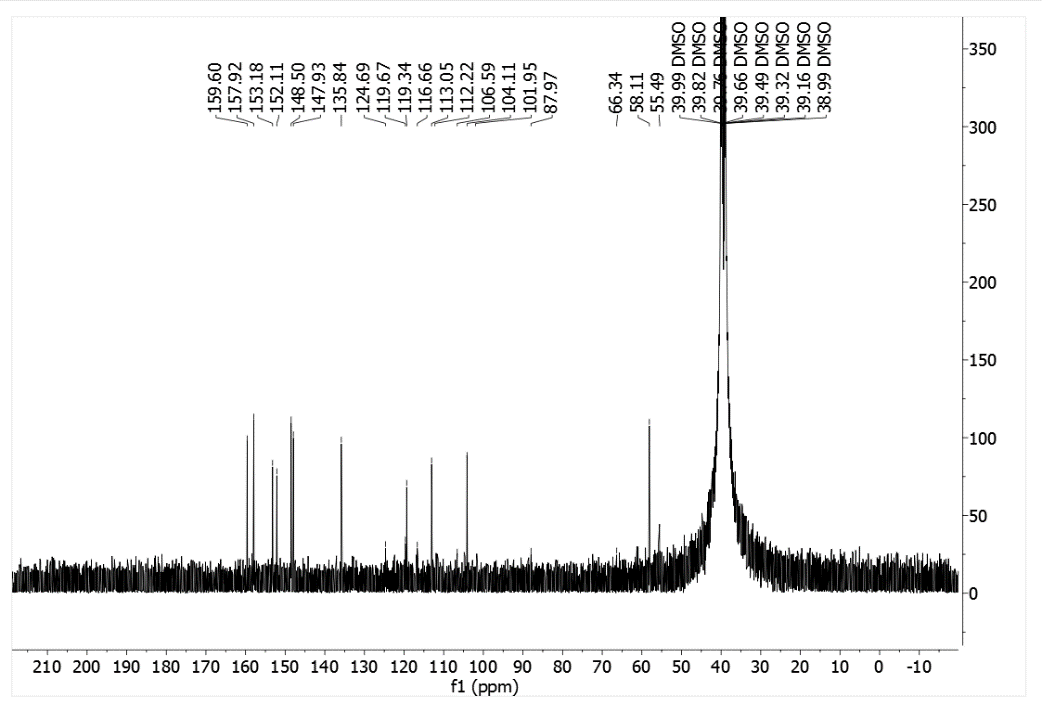
**

**Fig.S17. The ^1^H NMR spectrum of 2-Amino-4-(4-methoxyphenyl)-5-oxo-4,5-dihydropyrano[3,2-*c*]chromene-3-carbonitrile**

**2-Amino-4-(4-isopropylphenyl)-5-oxo-4,5-dihydropyrano[3,2-*c*]chromene-3-carbonitrile** Yellow solid, Melting point: 250-252 °C; FT-IR (ATR)/υ(cm^-1^): 3387, 3297, 3199, 2201, 1710, 1668, 1634, 1604; ^1^HNMR(500MHz,DMSO-d_6_) δ (ppm): 1.16 (d, 6H, *J* = 8Hz, 2CH_3_), 2.80-2.85 (m, 1H, CH), 4.41 (s, 1H, CH), 7.40-7.48 (m, 7H, Ar-H, NH_2_), 7.67 (t, 1H, *J* = 8Hz, Ar-H), 7.90 (d, 1H, *J* =7.5 Hz, Ar-H). ^13^C NMR (125 MHz, DMSO-d_6_) *δ* (ppm) 23.82, 33.06, 36.56, 58.02, 103.91, 104.20, 112.96, 116.56, 119.37, 122.34, 122.53, 124.71, 126.45, 127.50, 132.99, 140.75, 147.14, 152.10, 152.33, 153.30, 158.04, 159.56.

**
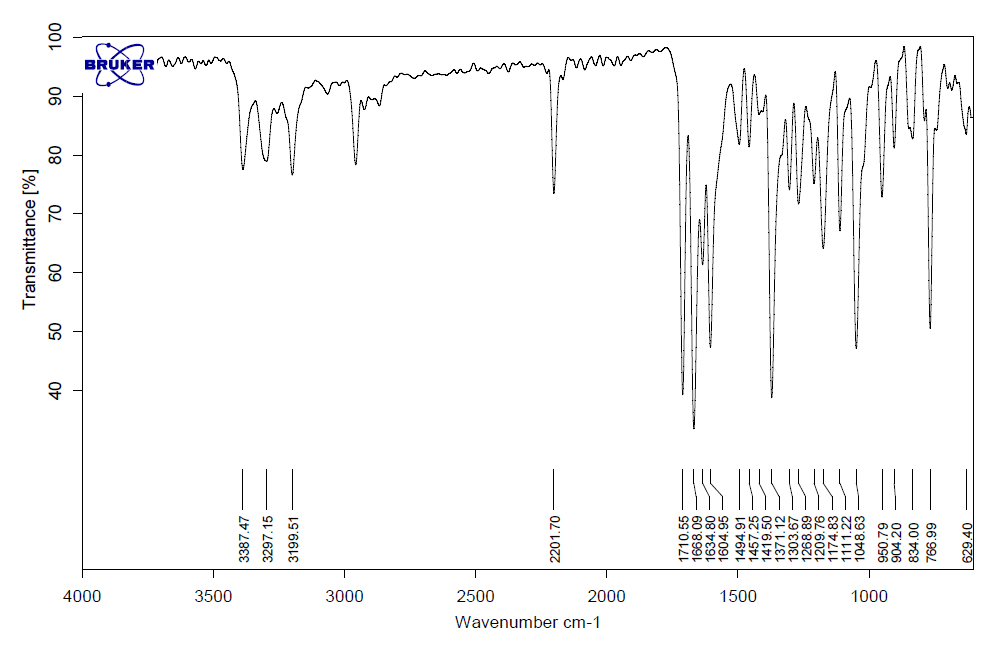
**

**Fig.S18. The FT-IR of 2-Amino-4-(4-isopropylphenyl)-5-oxo-4,5-dihydropyrano[3,2-*c*]chromene-3-carbonitrile**

**
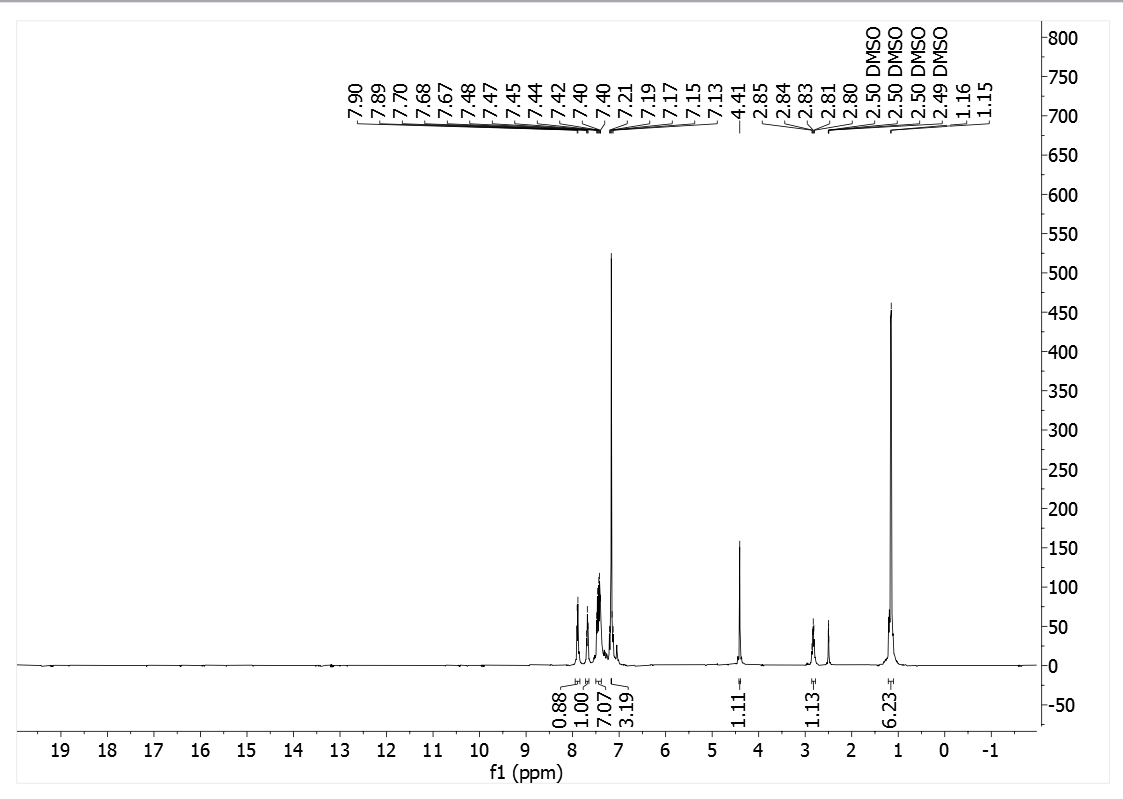
**

**Fig.S19. The ^1^H NMR spectrum of 2-Amino-4-(4-isopropylphenyl)-5-oxo-4,5-dihydropyrano[3,2-*c*]chromene-3-carbonitrile**

**
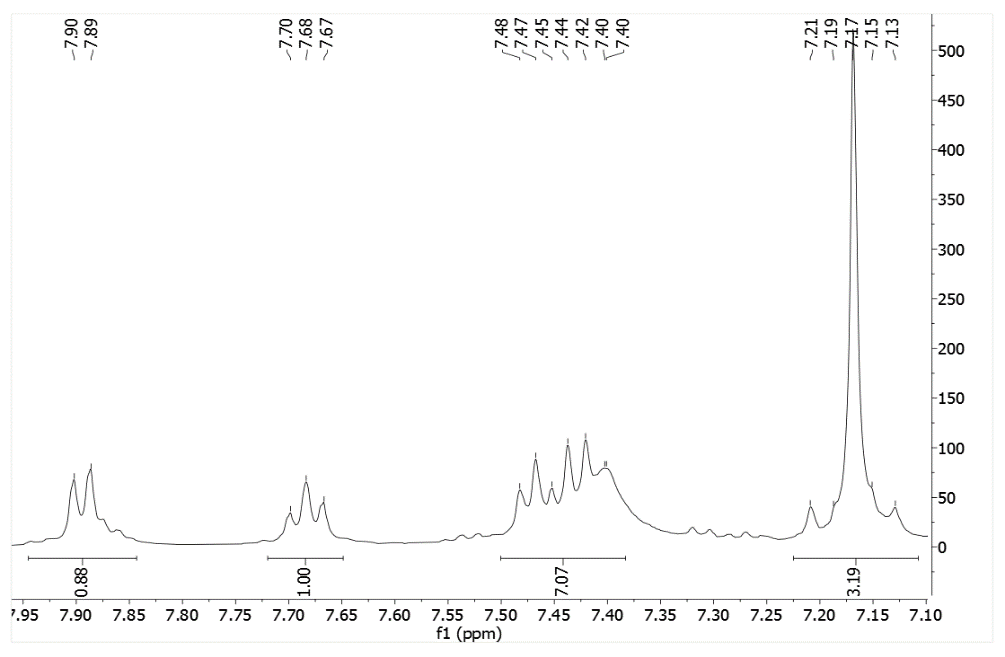
**

**Fig.S20. The ^1^H NMR spectrum of 2-Amino-4-(4-isopropylphenyl)-5-oxo-4,5-dihydropyrano[3,2-*c*]chromene-3-carbonitrile**

**
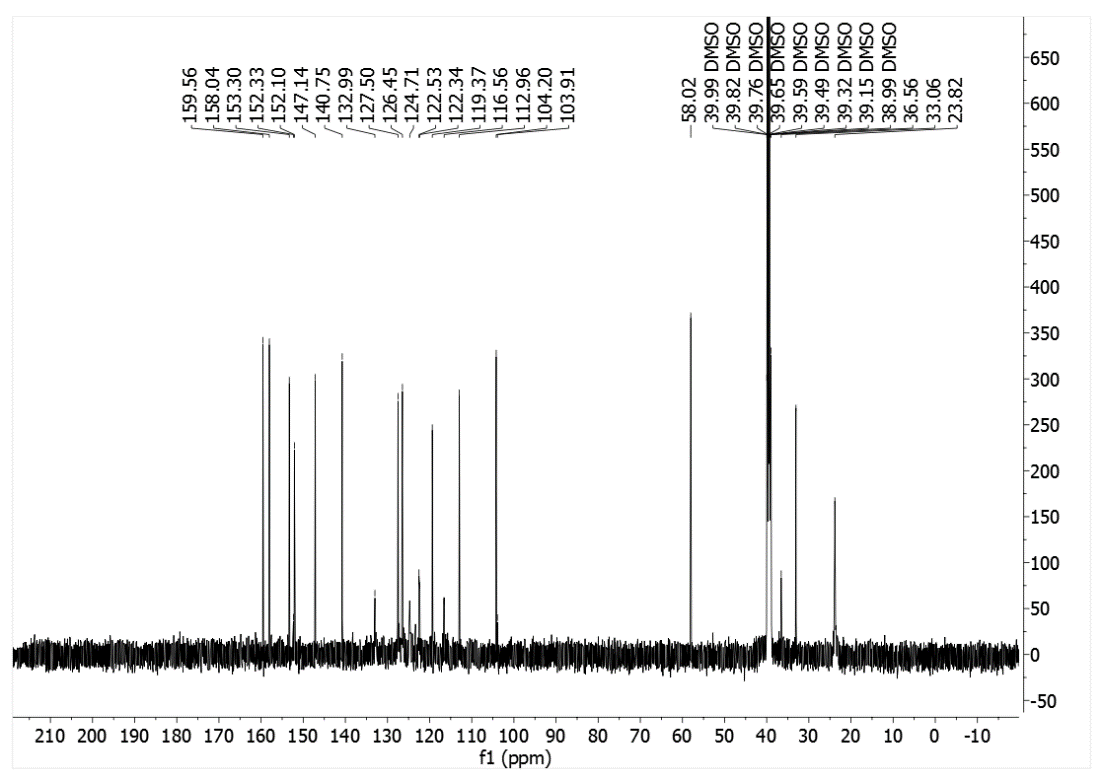
**

**Fig.S21. The ^1^H NMR spectrum of 2-Amino-4-(4-isopropylphenyl)-5-oxo-4,5-dihydropyrano[3,2-*c*]chromene-3-carbonitrile**

**2-Amino-4-(3-nitrophenyl)-5-oxo-4,5-dihydropyrano[3,2-*c*]chromene-3-carbonitrile**

Yellow solid, Melting point: 261-263 ºC, FT-IR (ATR)/υ(cm^-1^): 3401, 3317, 3191, 2202, 1700, 1668, 1605, 1527, 1380, 1207, 1112, 1055, 956, 733; ^1^HNMR (400MHz, DMSO-d_6_) δ( ppm): 4.73 (1H,s, CH),7.45-7.52 (2H, m, Ar-H), 7.57 (2H, s, NH_2_), 7.61-7.65 (1H, m, Ar-H), 7.72 (1H, td, *J_1_*=1.6 Hz, *J*_2_=8Hz, Ar-H), 7.81 (1H, d, *J*=8Hz, Ar-H), 7.92 (1H, dd, *J*_1_=1.6Hz, *J_2_*=8Hz, Ar-H), 8.11 (2H, d, *J*=8Hz, Ar-H)

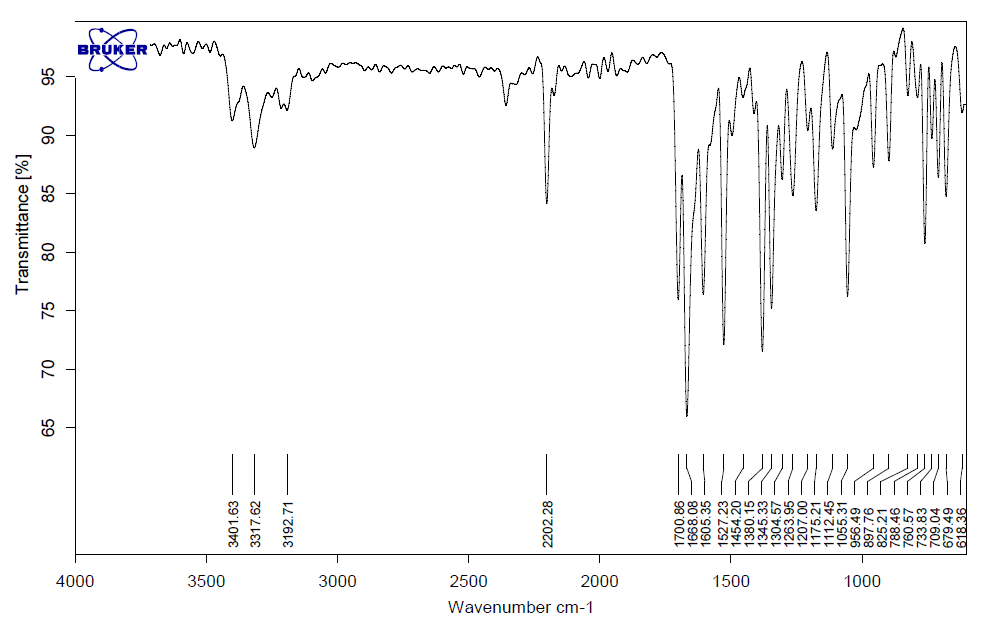


**Fig.S22. The FT-IR of 2-Amino-4-(3-nitrophenyl)-5-oxo-4,5-dihydropyrano[3,2-*c*]chromene-3-carbonitrile**


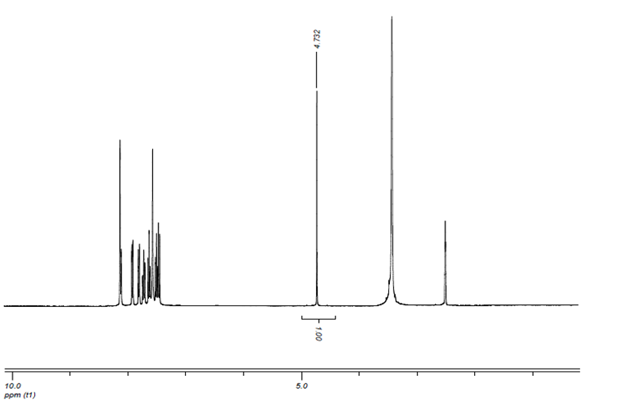


**Fig.S23. The ^1^H NMR spectrum of 2-Amino-4-(3-nitrophenyl)-5-oxo-4,5-dihydropyrano[3,2-*c*]chromene-3-carbonitrile**


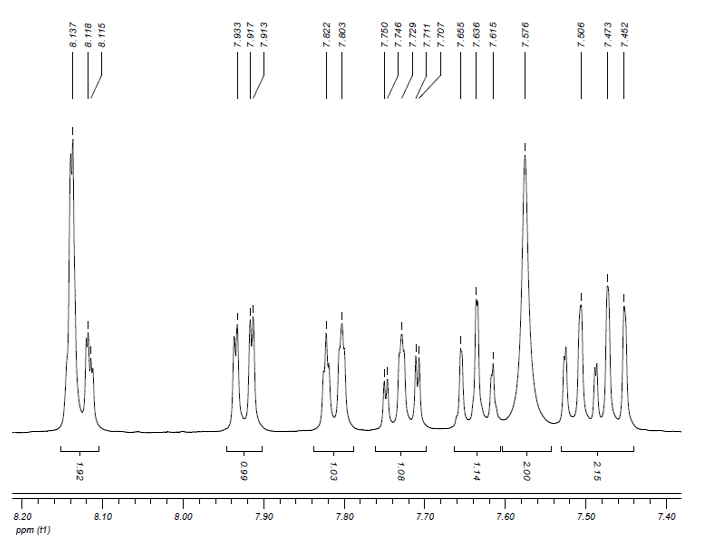


**Fig.S24. The ^1^H NMR spectrum of 2-Amino-4-(3-nitrophenyl)-5-oxo-4,5-dihydropyrano[3,2-*c*]chromene-3-carbonitrile**

**2-Amino-4-(2-nitrophenyl)-5-oxo-4,5-dihydropyrano[3,2-*c*]chromene-3-carbonitrile**

Pale yellow solid, Melting point: 259-261 ºC, FT-IR (ATR)/υ(cm^-1^): 3398, 3287, 3183, 2195, 1700, 1670, 1602; ^1^HNMR(500MHz, DMSO-d_6_) δ( ppm): 5.24 (s, 1H), 7.44-7.57 (m, 6H), 7.66 (t, 1H, *J* = 7.5 Hz), 7.73 (dt, 1H, *J* = 7.5 Hz), 7.90 (d, 2H, *J* = 8 Hz). ^13^C NMR (125MHz, DMSO-d6) *δ* (ppm) 31.56, 56.02, 103.32, 112.84, 116.67, 118.72, 122.48, 123.90, 124.08, 124.87, 131.29, 133.48, 137.36, 149.19, 152.18, 153.59, 158.61, 159.74.

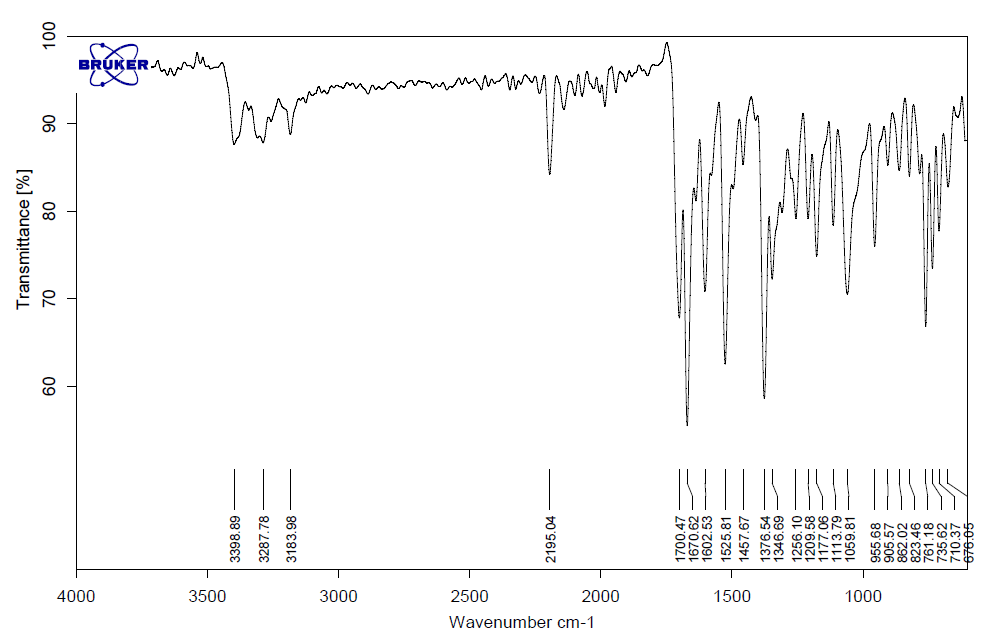


**Fig.S25. The FT-IR of 2-Amino-4-(2-nitrophenyl)-5-oxo-4,5-dihydropyrano[3,2-*c*]chromene-3-carbonitrile**


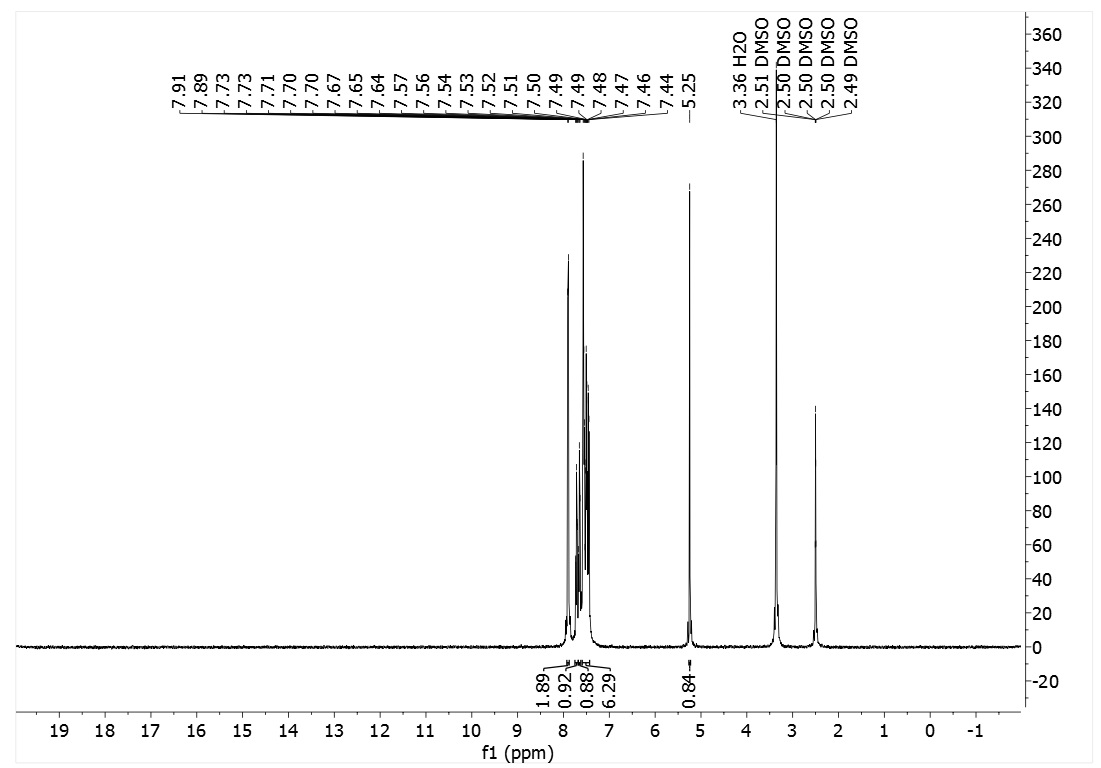


**Fig.S26. The ^1^H NMR spectrum of 2-Amino-4-(2-nitrophenyl)-5-oxo-4,5-dihydropyrano[3,2-*c*]chromene-3-carbonitrile**


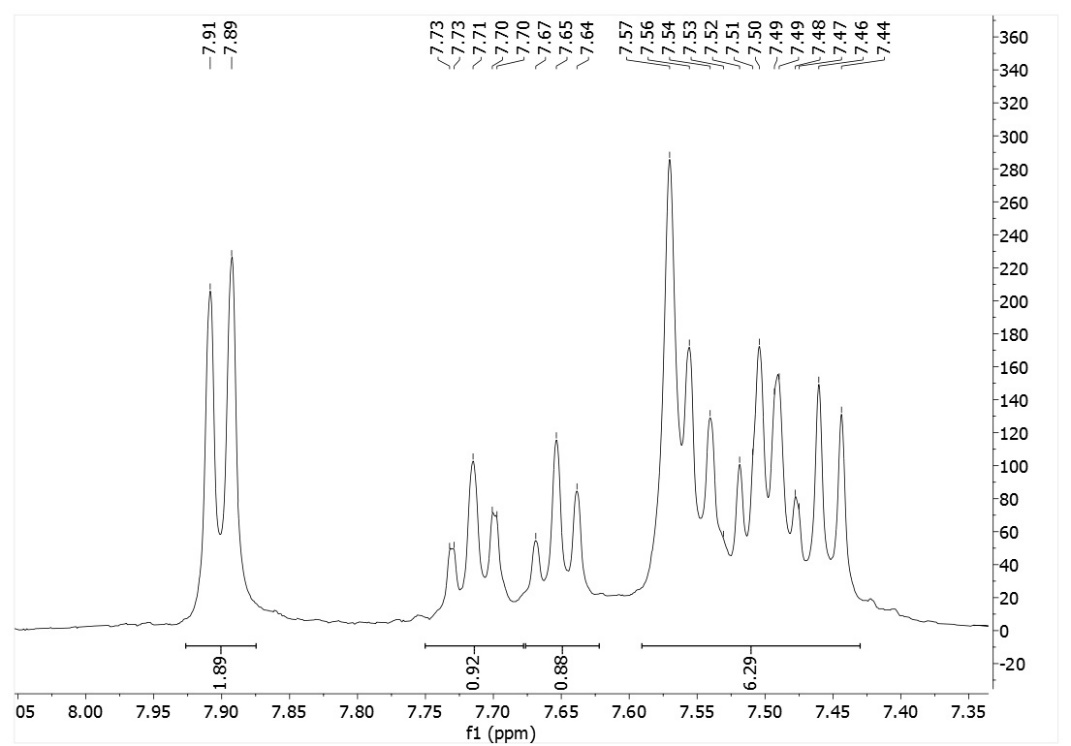


**Fig.S27. The ^1^H NMR spectrum of 2-Amino-4-(2-nitrophenyl)-5-oxo-4,5-dihydropyrano[3,2-*c*]chromene-3-carbonitrile**


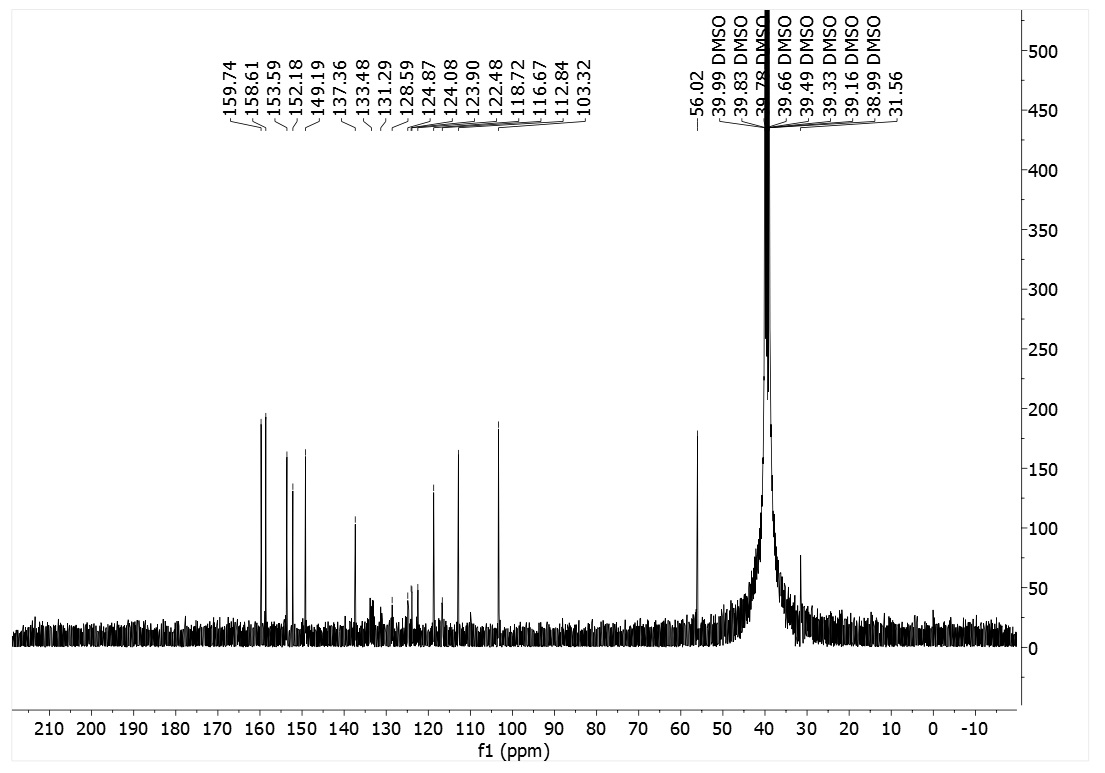


**Fig.S28. The ^1^H NMR spectrum of 2-Amino-4-(2-nitrophenyl)-5-oxo-4,5-dihydropyrano[3,2-*c*]chromene-3-carbonitrile**

**2-Amino-4-(2-chlorophenyl)-5-oxo-4,5-dihydropyrano[3,2-*c*]chromene-3-carbonitrile**

White Solid, Melting point: 273-275 ºC**,** FT-IR (ATR)/υ(cm^-1^): 3392, 3281, 3175, 2200, 1704, 1671, 1601; ^1^HNMR (500MHz, DMSO-d_6_) δ( ppm): 4.98 (1H, s, CH ), 7.26-7.33 (m, 3H, Ar-H), 7.40-7.52 (m, 5H, Ar-H, NH_2_), 7.65-7.74 (m, 2H, Ar-H), 7.90-7.92 (dd, 1H, *J_1_*=1Hz, *J_2_*=7.5Hz, Ar-H);^13^C NMR (125 MHz, DMSO-d_6_) δ( ppm): 56.44, 86.64, 102.92, 112.21, 112.82, 113.47, 118.84, 127.72, 128.82, 129.63, 132.40, 134.28, 140.21, 152.20, 154.04, 158.03, 158.13, 159.42.

**
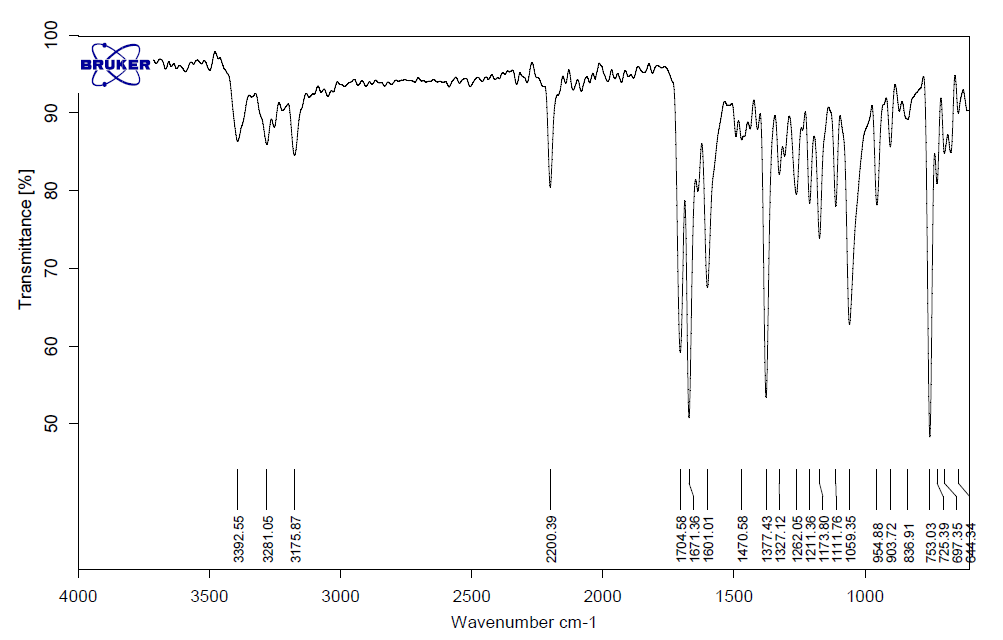
**

**Fig.S29. The FT-IR of 2-Amino-4-(2-chlorophenyl)-5-oxo-4,5-dihydropyrano[3,2-*c*]chromene-3-carbonitrile**


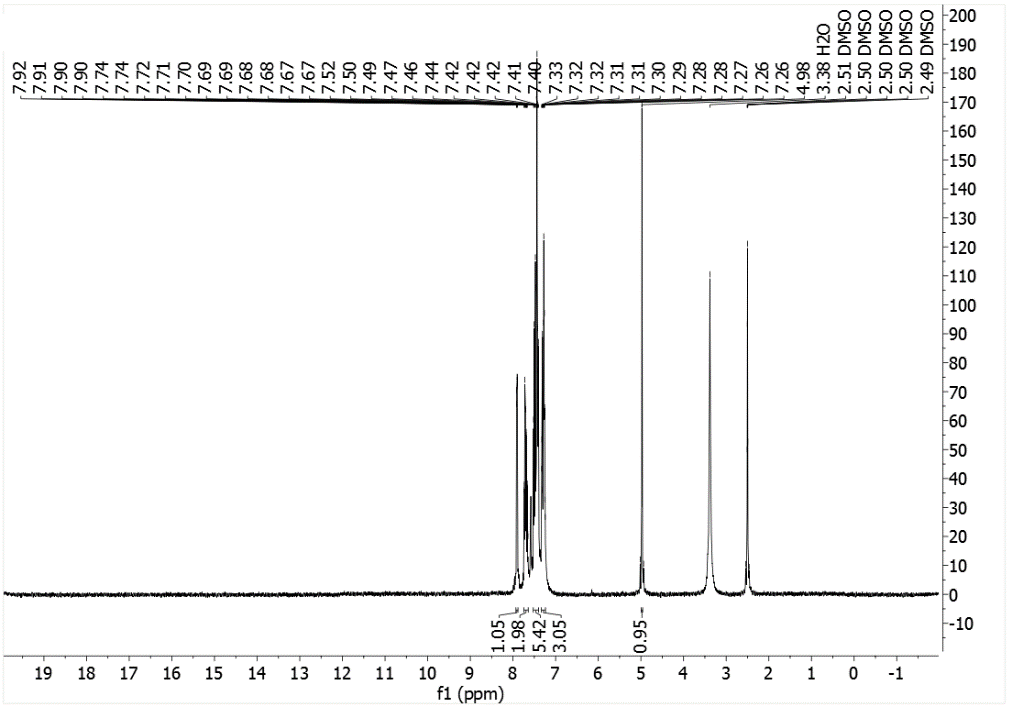


**Fig.S30. The ^1^H NMR spectrum of 2-Amino-4-(2-chlorophenyl)-5-oxo-4,5-dihydropyrano[3,2-*c*]chromene-3-carbonitrile**


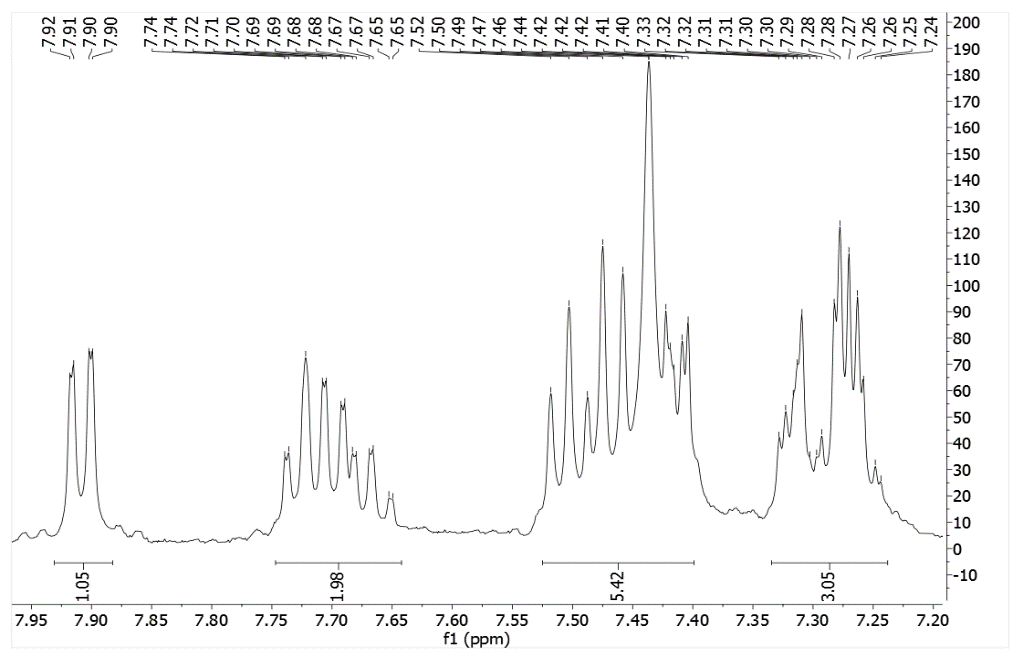


**Fig.S31. The ^1^H NMR spectrum of 2-Amino-4-(2-chlorophenyl)-5-oxo-4,5-dihydropyrano[3,2-*c*]chromene-3-carbonitrile**


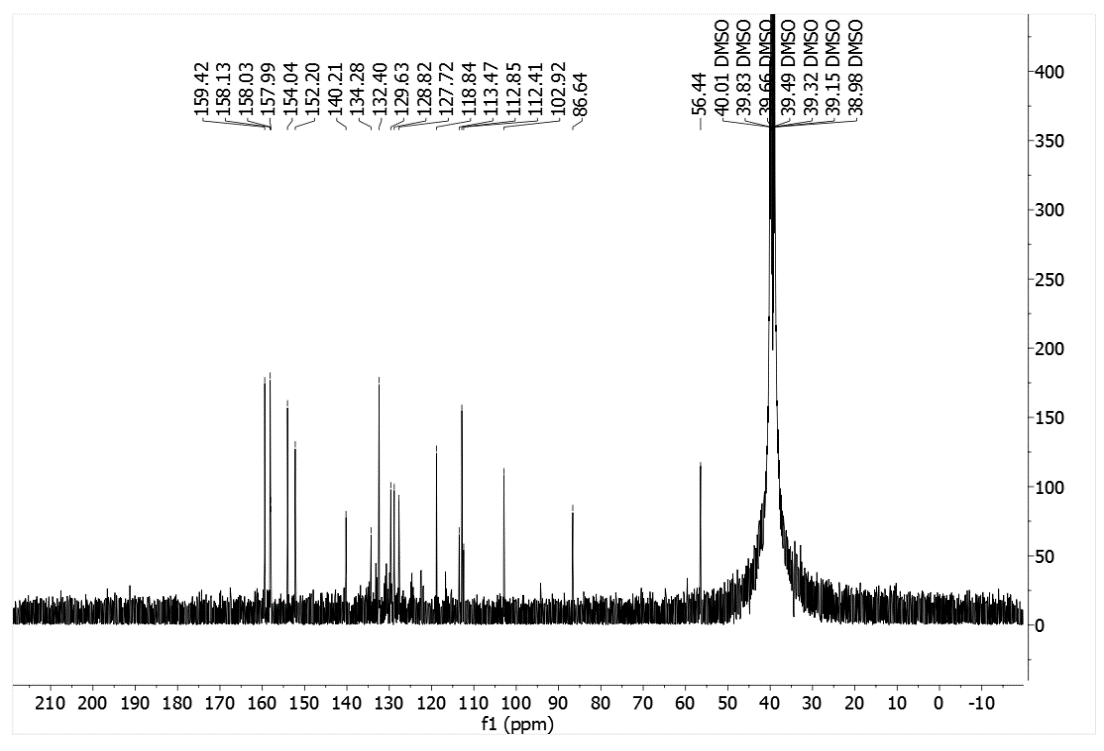


**Fig.S32. The ^1^H NMR spectrum of 2-Amino-4-(2-chlorophenyl)-5-oxo-4,5-dihydropyrano[3,2-*c*]chromene-3-carbonitrile**

**2-Amino-4-(2,6-dichlorophenyl)-5-oxo-4,5-dihydropyrano[3,2-*c*]chromene-3-carbonitrile** White Solid, Melting point: 273-276 ºC**,** FT-IR (ATR)/υ(cm^-1^): 3415, 3276, 3170, 2200, 1703, 1669, 1633. ^1^HNMR (400MHz, DMSO-d_6_) δ( ppm): 5.52 (d, 1H,CH), 7.29-7.38 (m, 2H, Ar-H), 7.46-7.52 (m, 5H, Ar-H, NH_2_), 7.74 (t, 1H, *J*=8.4 Hz, Ar-H), 7.88 (d, 1H, *J*=7.5, Ar-H).

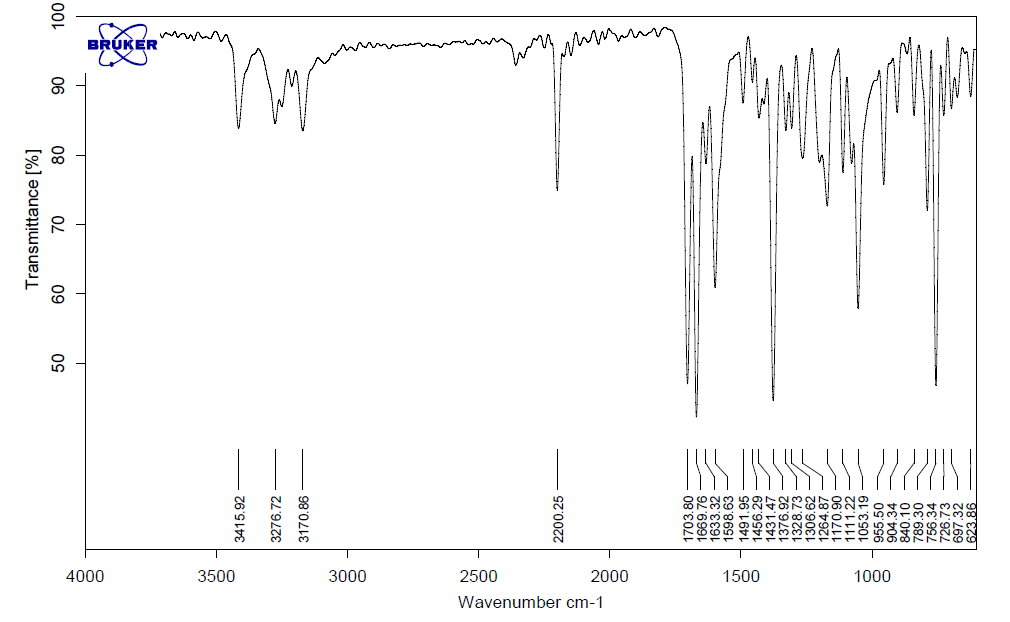


**Fig.S33. The FT-IR of 2-Amino-4-(2,6-dichlorophenyl)-5-oxo-4,5-dihydropyrano[3,2-*c*]chromene-3-carbonitrile**


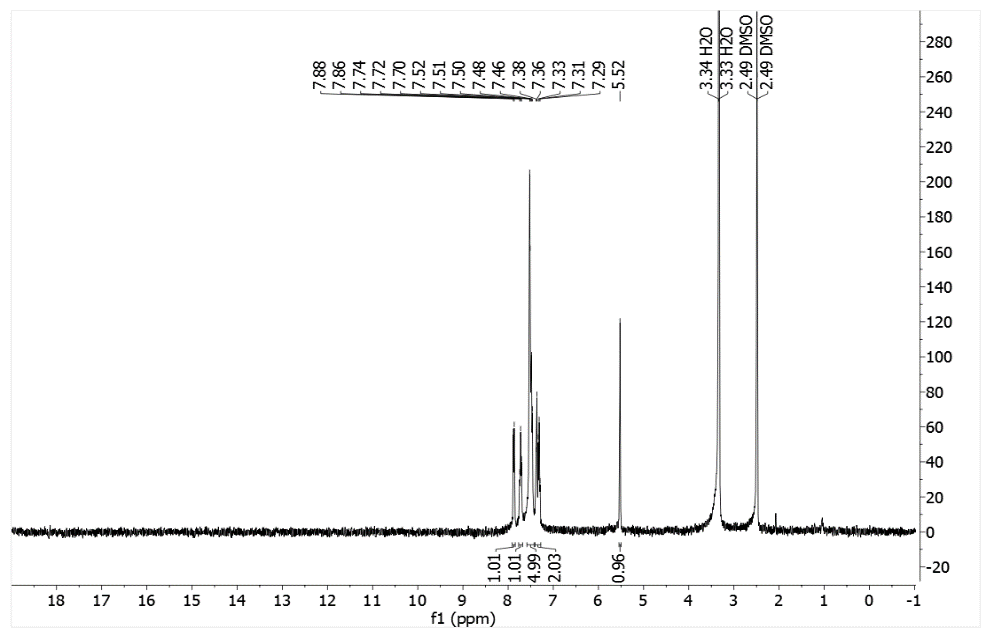


**Fig.S34. The ^1^H NMR spectrum of 2-Amino-4-(2,6-dichlorophenyl)-5-oxo-4,5-dihydropyrano[3,2-*c*]chromene-3-carbonitrile**


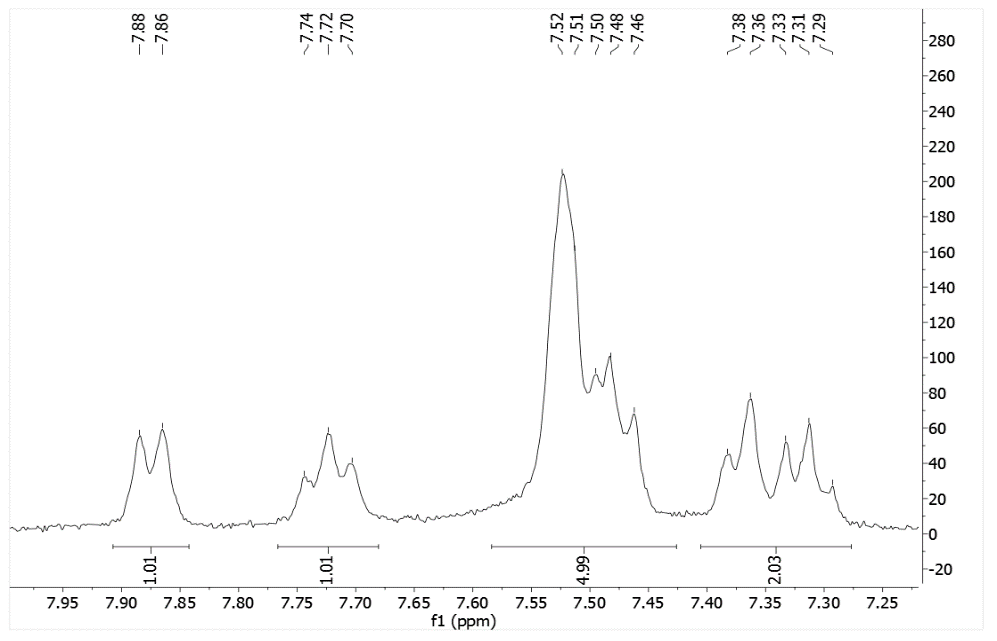


**Fig.S35. The ^1^H NMR spectrum of 2-Amino-4-(2,6-dichlorophenyl)-5-oxo-4,5-dihydropyrano[3,2-*c*]chromene-3-carbonitrile**

**2-Amino-5-oxo-4-phenyl-4,5-dihydropyrano[3,2-*c*]chromene-3-carbonitrile**

White Solid, Melting point: 255-257 ºC, FT-IR (ATR)/υ(cm^-1^): 3371, 3284, 3176, 2196, 1706, 1671, 1604. ^1^HNMR (500MHz, DMSO-d_6_) δ( ppm): 4.45 (1H, s, CH ), 7.22-7.33 (5H, m, Ar-H), 7.42-7.49 (4H, m, Ar-H,NH_2_), 7.71 (1H, t, *J=*8Hz, Ar-H), 7.91 (1H, d, *J*=8Hz, Ar-H). ^13^C NMR (125 MHz, DMSO-d_6_) δ( ppm): 37.00, 57.94, 103.99, 112.95, 116.61, 119.28, 122.38, 122.56, 124.77, 127.13, 127.65, 128.53, 132.83, 133.04, 143.36, 152.13, 153.42, 157.98, 159.55.

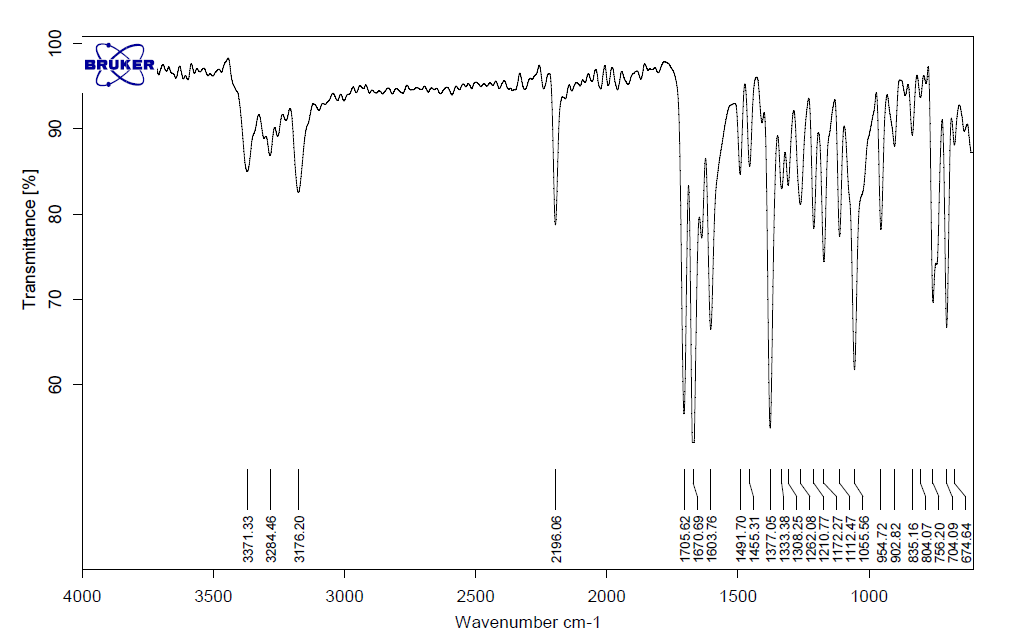


**Fig.S36. The FT-IR of 2-Amino-5-oxo-4-phenyl-4,5-dihydropyrano[3,2-*c*]chromene-3-carbonitrile**


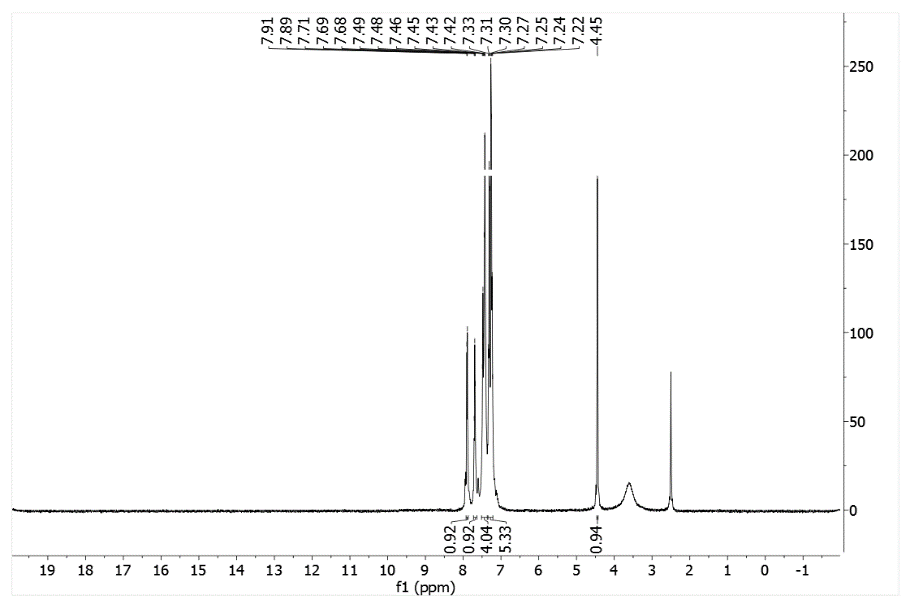


**Fig.S37. The ^1^H NMR spectrum of 2-Amino-5-oxo-4-phenyl-4,5-dihydropyrano[3,2-*c*]chromene-3-carbonitrile**


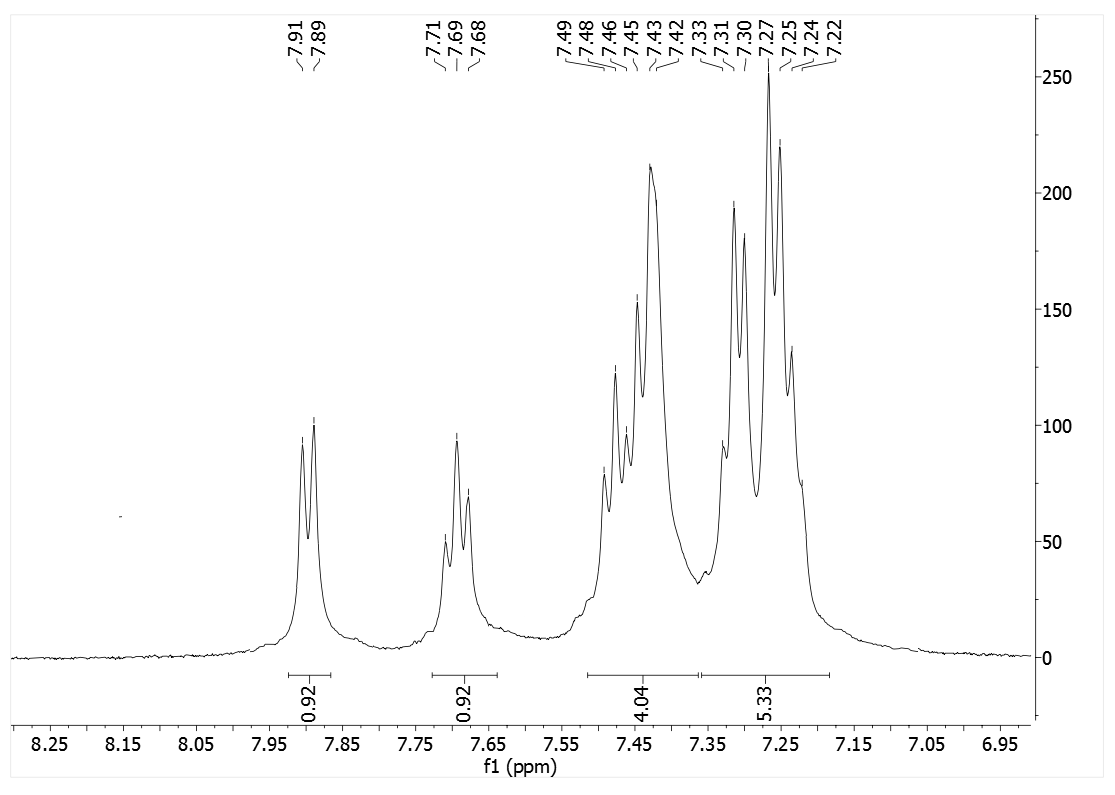


**Fig.S38. The ^1^H NMR spectrum of 2-Amino-5-oxo-4-phenyl-4,5-dihydropyrano[3,2-*c*]chromene-3-carbonitrile**


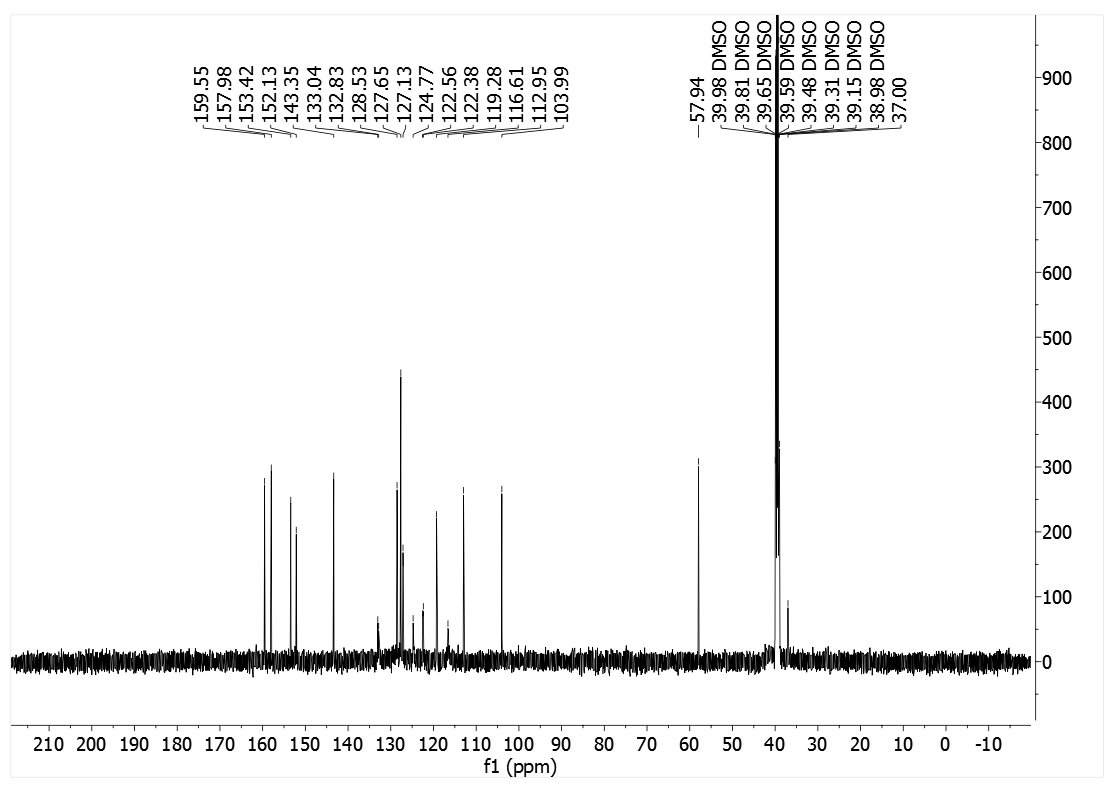


**Fig.S39. The ^1^H NMR spectrum of 2-Amino-5-oxo-4-phenyl-4,5-dihydropyrano[3,2-*c*]chromene-3-carbonitrile**

**2-Amino-5-oxo-4-(pyridin-3-yl)- 4,5-dihydropyrano[3,2-*c*]chromene-3-carbonitrile**

Cream powder**,** Melting point: 250- 252 °C; FT-IR (ATR)/υ(cm^-1^): 3317, 3204, 2199, 1702, 1671, 1604; ^1^HNMR(400MHz,DMSO-d_6_) δ (ppm): 4.55 (s,1H), 7.35 (dd, 1H *J_1_*=2.8 Hz, *J*_2_=4.8 Hz), 7.45 -7.49 (m, 2H), 7.52 (s, 2H), 7.69-7.77 (m, 2H), 7.90 (dd, 1H, *J*_1_=1.2Hz, *J_2_*=6.8Hz), 8.46 (d, 1H, *J*=3.6 Hz), 8.55 (d, 1H, *J*=1.6 Hz); ^13^C NMR (100 MHz, DMSO-d_6_) *δ* (ppm) ): 35.1, 57.4, 103.4, 113.4, 117.0, 119.5, 123.0, 124.2, 125.1, 133.5, 135.8, 139.2, 148.8, 149.5, 152.7, 154.2, 158.5, 160.0.

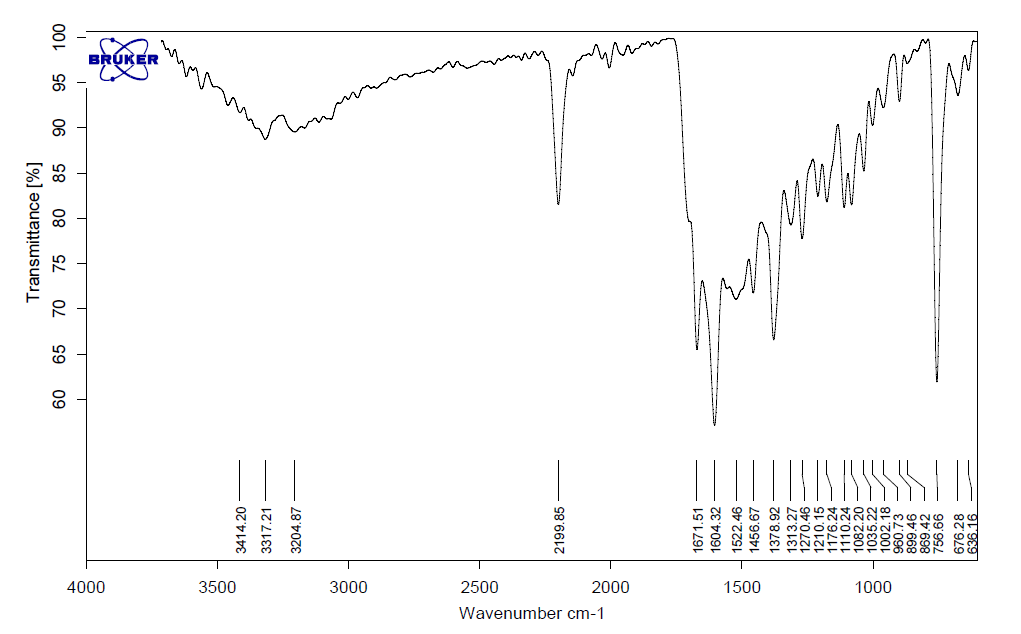


**Fig.S40. The FT-IR of 2-Amino-5-oxo-4-(pyridin-3-yl)- 4,5-dihydropyrano[3,2-*c*]chromene-3-carbonitrile**


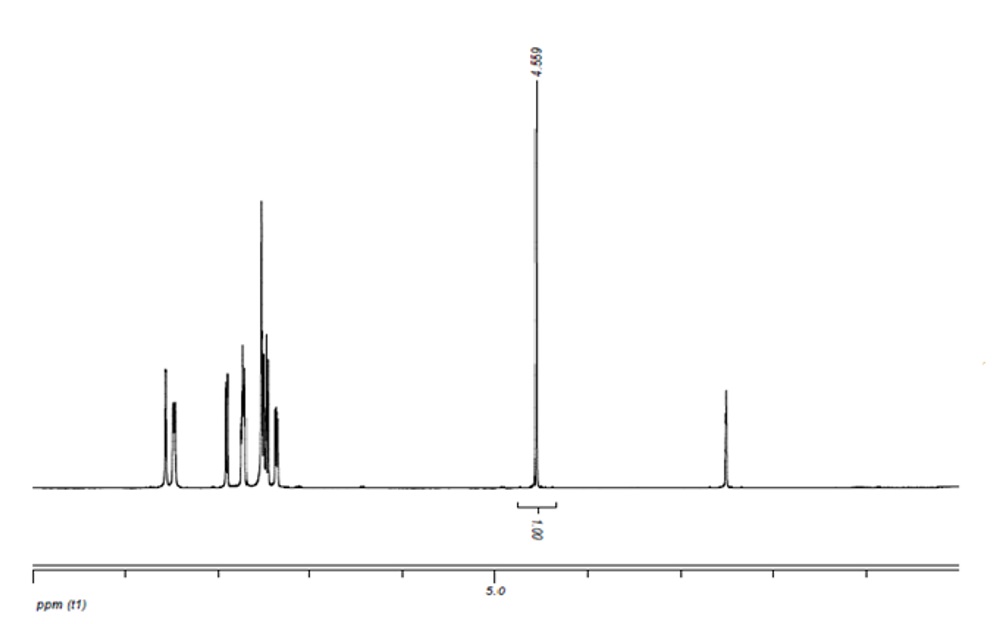


**Fig.S41. The ^1^H NMR spectrum of 2-Amino-5-oxo-4-(pyridin-3-yl)- 4,5-dihydropyrano[3,2-*c*]chromene-3-carbonitrile**


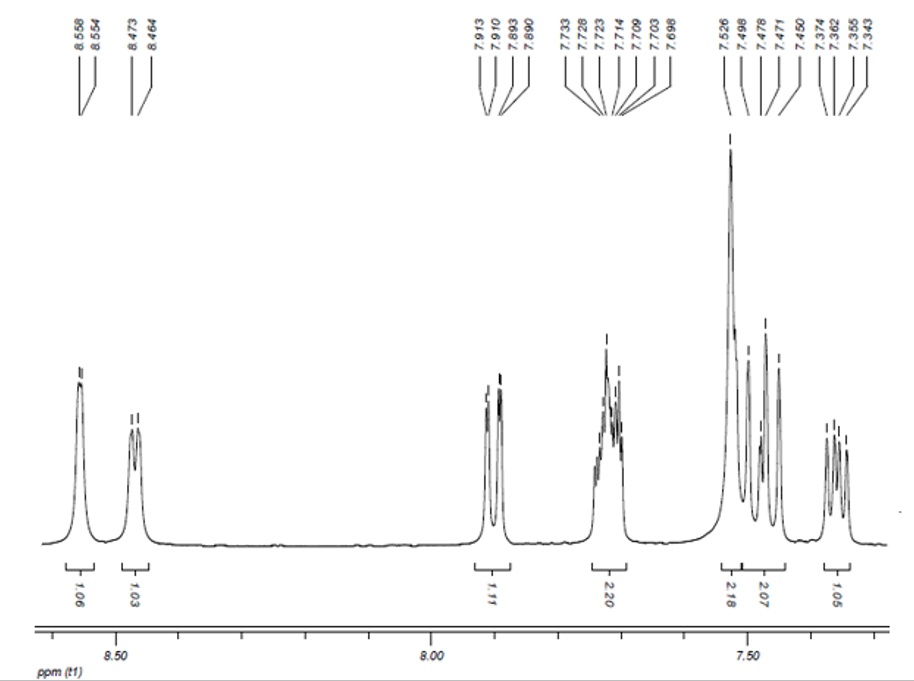


**Fig.S42. The ^1^H NMR spectrum of 2-Amino-5-oxo-4-(pyridin-3-yl)- 4,5-dihydropyrano[3,2-*c*]chromene-3-carbonitrile**


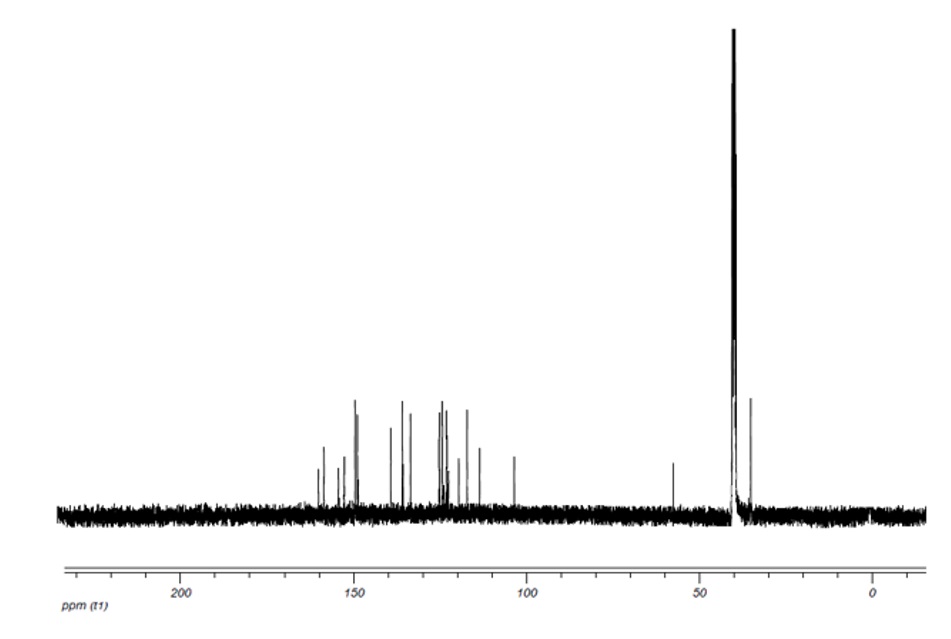


**Fig.S43. The ^1^H NMR spectrum of 2-Amino-5-oxo-4-(pyridin-3-yl)- 4,5-dihydropyrano[3,2-*c*]chromene-3-carbonitrile**

**2-amino-5-oxo-4-pentyl-4H,5H-pyrano[3,2-c]chromene-3-carbonitrile**

Pale yellow solid, m. p. 188-190 ºC. FT-IR (ATR) ῡ (cm^-1^): 3323, 3196, 2202, 1701, 1665, 1606 .^1^H NMR (400 MHz, DMSO-*d_6_*)/ δ ppm: 0.84 (t, *J* = 6.8 Hz, 3H, CH), 1.25 (d*, J* = 3.2 Hz, 6H, CH), 1.54 -1.61 (m, 1H, CH), 1.72-1.78 (m, 1H, CH), 3.46 (t, *J =* 4 Hz, 1H, CH), 7.34 (s, 2H, NH_2_), 7.47-7.51 (m, 2H, Ar-H), 7.73 (td, *J_1_ =* 1.6 Hz, *J_2_* = 7.2 Hz, 1H, Ar-H), 7.83 (dd, *J* _1_= 1.2 Hz, *J_2_* = 8.0 Hz, 1H, Ar-H).


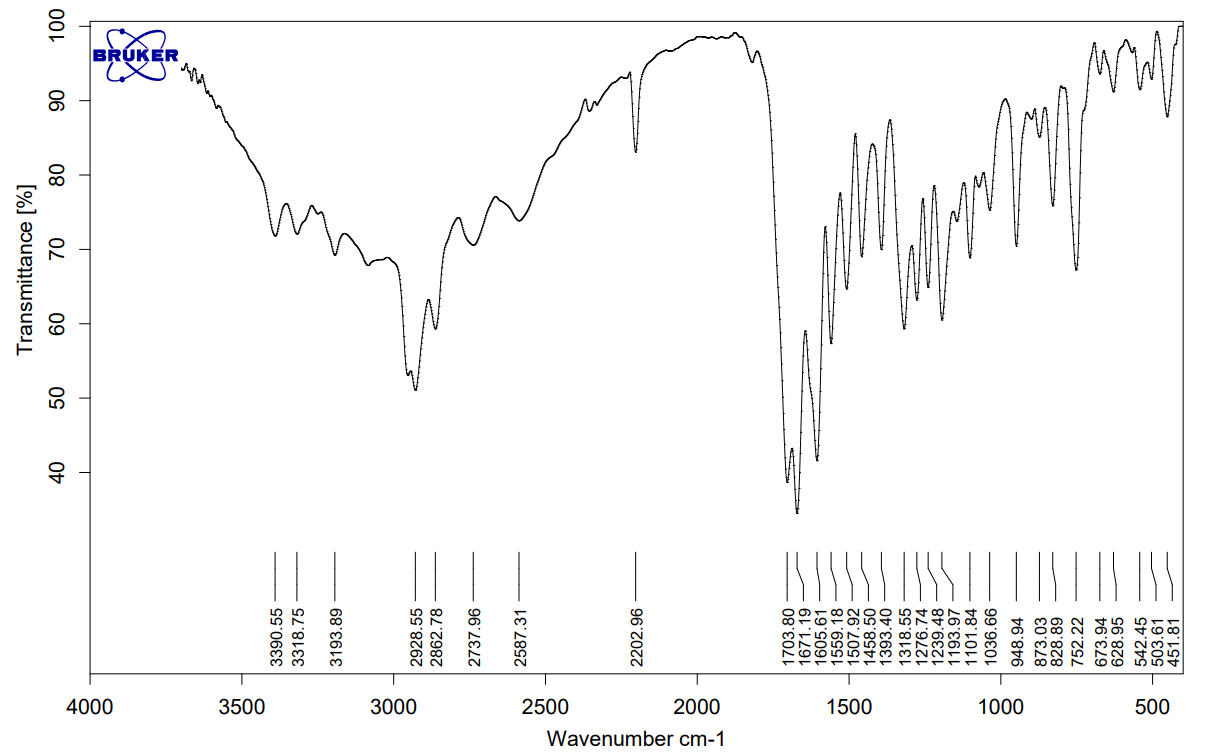


**Fig.S44. The FT-IR of 2-amino-5-oxo-4-pentyl-4H,5H-pyrano[3,2-c]chromene-3-carbonitrile**

**
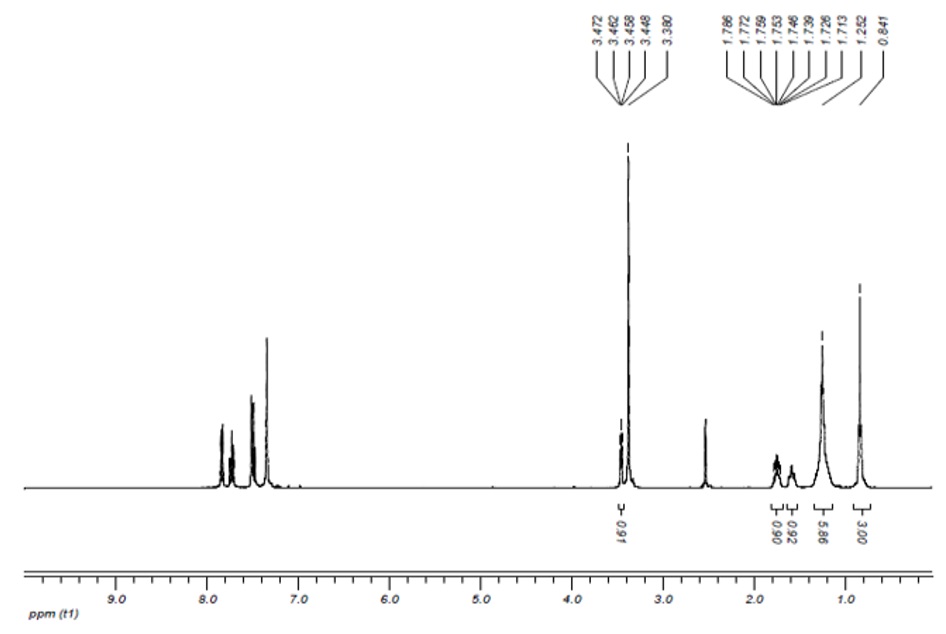
**

**Fig.S45. The ^1^H NMR spectrum of 2-amino-5-oxo-4-pentyl-4H,5H-pyrano[3,2-c]chromene-3-carbonitrile**

**
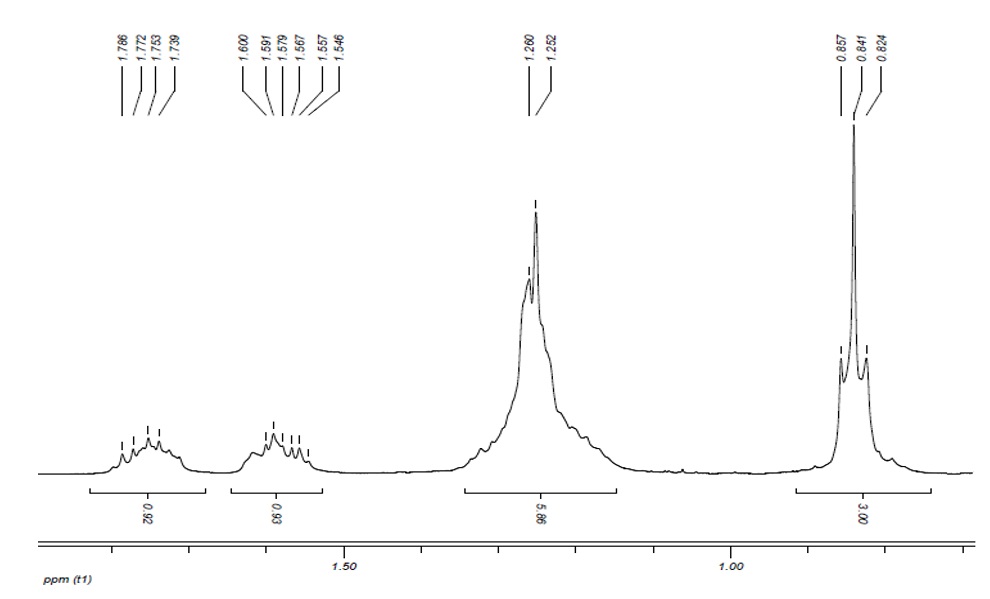
**

**Fig.S46. The ^1^H NMR spectrum of 2-amino-5-oxo-4-pentyl-4H,5H-pyrano[3,2-c]chromene-3-carbonitrile**

**
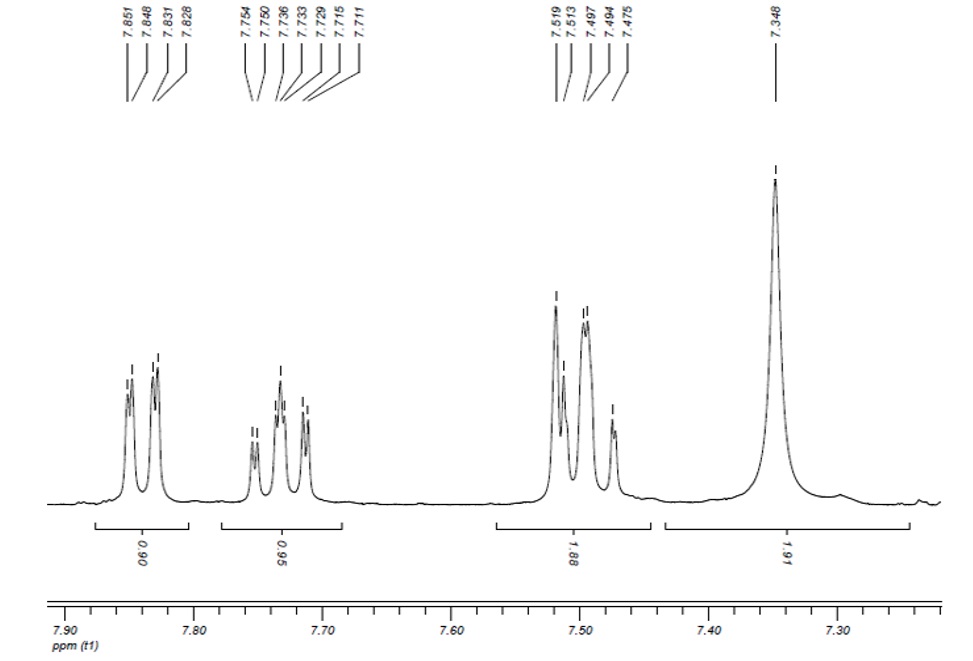
**

**Fig.S47. The ^1^H NMR spectrum of 2-amino-5-oxo-4-pentyl-4H,5H-pyrano[3,2-c]chromene-3-carbonitrile**

**2-Amino-7,7-dimethyl-4-(4-nitrophenyl)-5-oxo-5,6,7,8-tetrahydrobenzo[*b*]pyran**

Yellow solid, m.p. 181-217 ºC FT-IR (ATR) ῡ (cm^-1^): 3421, 3314, 2190, 1682, 1654, 1515, 1343, 1213. ^1^H NMR (400 MHz, DMSO-*d_6_*)/ δ ppm: 0.94 (s, 3H), 1.03 (s, 3H), 2.12 (d, *J* = 16.0 Hz, 1H), 2.27 (d, *J* = 16.0 Hz, 1H), 2.49-2.52 (m, 2H), 4.35 (s, 1H), 7.17 (s, 2H, NH_2_), 7.44 (d, *J* = 8 Hz, 2H), 8.17 (d, *J* = 8 Hz, 2H)


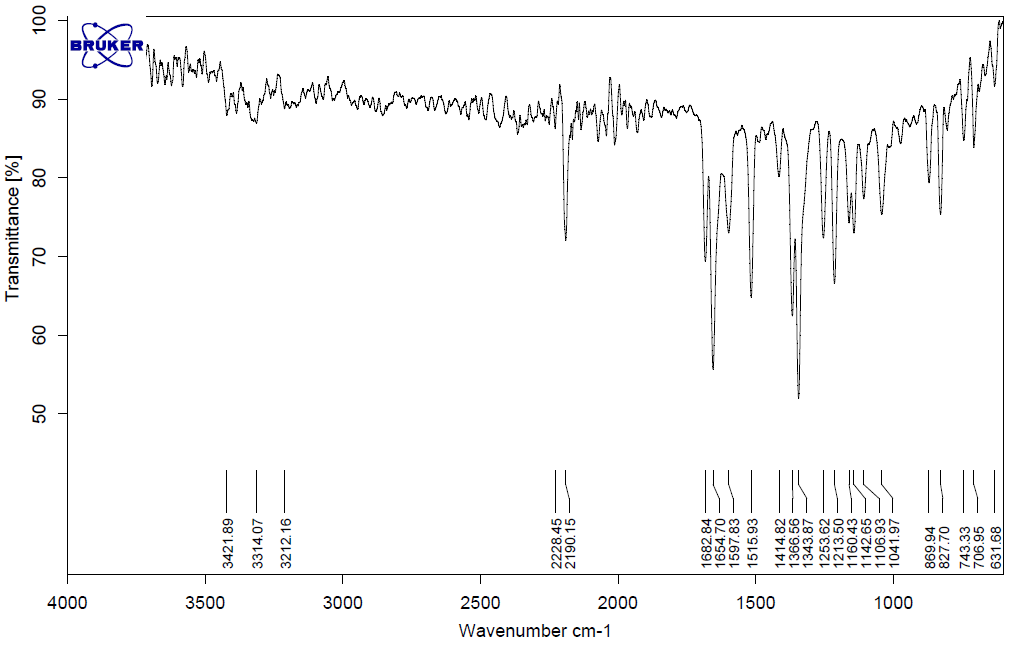


**Fig.S48. The FT-IR of** **2-Amino-7,7-dimethyl-4-(4-nitrophenyl)-5-oxo-5,6,7,8-tetrahydrobenzo[*b*]pyran**


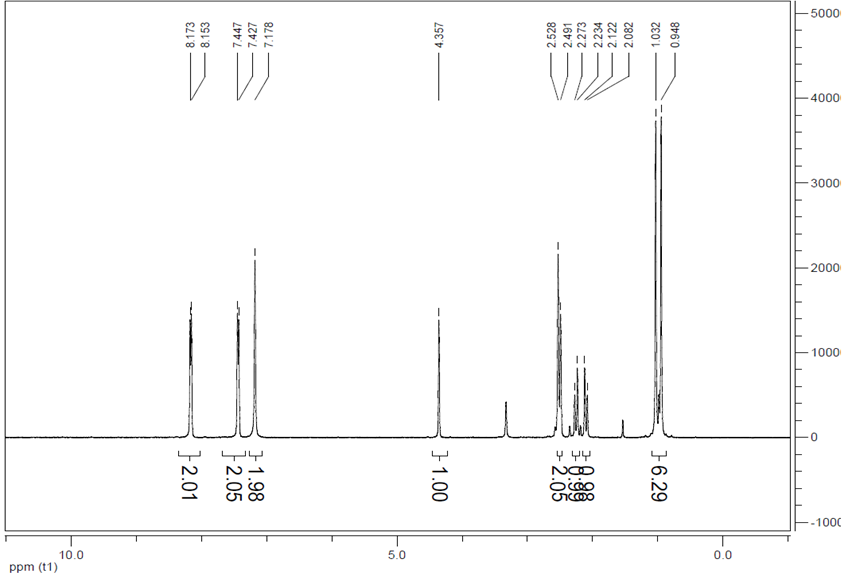


**Fig.S49. The ^1^H NMR spectrum of 2-Amino-7,7-dimethyl-4-(4-nitrophenyl)-5-oxo-5,6,7,8-tetrahydrobenzo[*b*]pyran**

**2-Amino-3-cyano-4-(4-fluorophenyl)-7,7-dimethyl-5-oxo-4*H*-5,6,7,8-tetrahydrobenzo[*b*]pyran** .

White powder, m.p. 191-194 ºC. FT-IR (ATR) ῡ (cm^-1^): 3405, 3214, 2201, 1656, 1604, 1216.

^1^H NMR (400 MHz, DMSO-*d_6_*)/ δ ppm: 0.93 (s, 3 H), 1.02 (s, 3 H), 2.11 (d, *J* = 16.0 Hz, 1 H), 2.25 (d, *J* = 16.0 Hz, 1 H), 2.45–2.54 (m, 2 H), 4.18 (s, 1 H), 7.02 (s, 2 H, NH_2_), 7.09-7.16 (m, 4 H) .


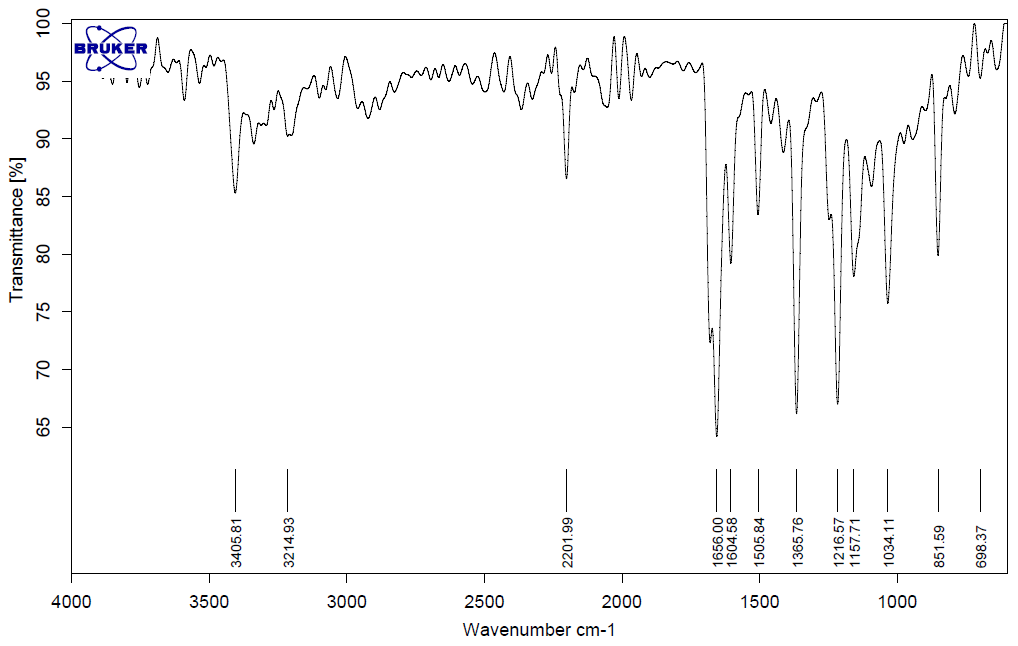


**Fig.S50. The FT-IR of 2-Amino-3-cyano-4-(4-fluorophenyl)-7,7-dimethyl-5-oxo-4*H*-5,6,7,8-tetrahydrobenzo[*b*]pyran**

**2-Amino-3-cyano-4-(4-bromophenyl)-7,7-dimethyl-5-oxo-4*H*-5,6,7,8 -tetrahydrobenzo [*b*]pyran**

White powder, m.p. 199-200 ºC.

FT-IR (ATR) ῡ (cm^-1^): 3399, 3306, 3209, 2188, 1681, 1653, 1213.


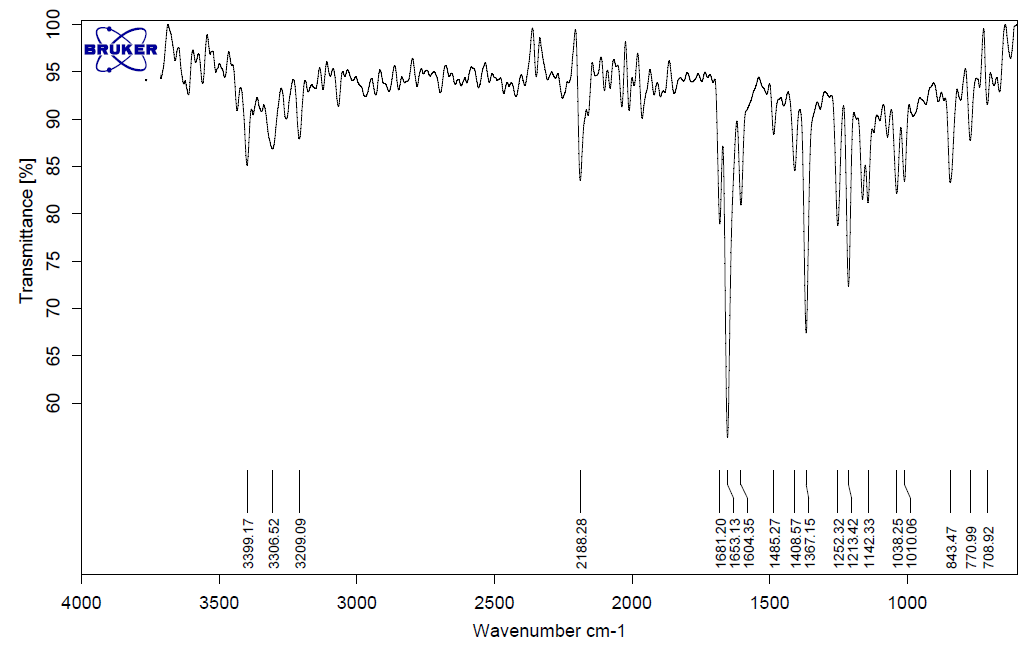


**Fig.S 51. The FT-IR of 2-Amino-3-cyano-4-(4-bromophenyl)-7,7-dimethyl-5-oxo-4*H*-5,6,7,8 -tetrahydrobenzo [*b*]pyran**

**2-Amino-4-(4-chlorophenyl)-3-cyano-7,7-dimethyl-5-oxo-4*H*-5,6,7,8-tetrahydrobenzo[*b*]pyran**

White solid, m.p. 215-216 ºC FT-IR (ATR) ῡ (cm^-1^): 3487, 3377, 3179, 2188, 1674, 1632, 1215 ^1^H NMR (400 MHz, DMSO-*d_6_*)/ δ ppm: 0.95 (s, 3H), 1.01 (s, 3H), 2.10 (d, *J* = 16.0 Hz, 1H), 2.25 (d, *J* = 16.0 Hz, 1H), 2.44-2.53 (m, 2H), 4.18 (s, 1H), 7.08 (s, 2H, NH_2_), 7.16 (d, *J* = 7.6 Hz, 2H), 7.34 (d, *J* = 7.6 Hz, 2H) ^13^C NMR (DMSO-d_6_, 100 MHz)/ δ ppm: 27.31, 28.80, 32.24, 35.60, 50.40, 58.23, 112.80, 120.07, 128.76, 129.59, 131.61, 144.21, 158.96, 163.08, 196.13


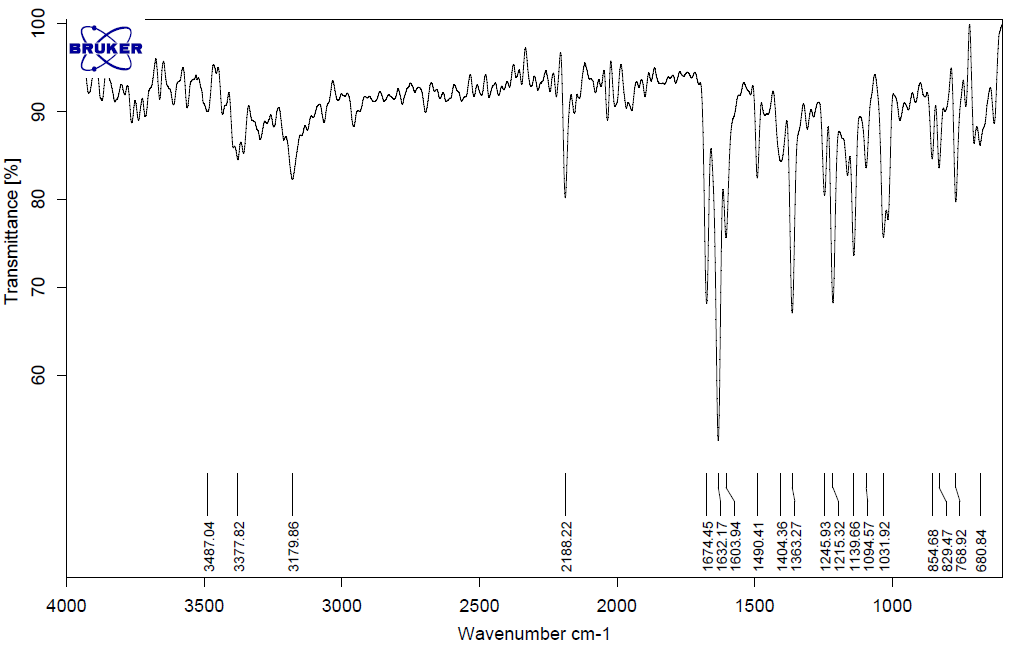


**Fig.S 52. The FT-IR of** **2-Amino-4-(4-chlorophenyl)-3-cyano-7,7-dimethyl-5-oxo-4*H*-5,6,7,8-tetrahydrobenzo[*b*]pyran**

**Fig.S 53. The ^1^H NMR spectrum of 2-Amino-4-(4-chlorophenyl)-3-cyano-7,7-dimethyl-5-oxo-4*H*-5,6,7,8-tetrahydrobenzo[*b*]pyran**

**Fig.S 54. The ^1^H NMR spectrum of 2-Amino-4-(4-chlorophenyl)-3-cyano-7,7-dimethyl-5-oxo-4*H*-5,6,7,8-tetrahydrobenzo[*b*]pyran**

**2-Amino-3-cyano-4-(4-hydroxyphenyl)-7,7-dimethyl-5-oxo-4*H*-5,6,7,8-tetrahydrobenzo[*b*]pyran**

Pale yellow solid, m.p. 213-215 ºC FT-IR (ATR) ῡ (cm^-1^): 3308, 3194, 2201, 1680, 1653, 1213

^1^H NMR (400 MHz, DMSO-*d_6_*)/ δ ppm: 0.93 (s, 3H), 1.02 (s, 3H), 2.10 (d, *J* = 16.0 Hz, 1H), 2.24 (d, *J* = 16.0 Hz, 1H), 2.42–2.53 (m, 2H), 4.04 (s, 1H), 6.63-6.65 (d, *J* = 8.4 Hz, 2H, Ar-H), 6.90-6.92 (m, 4H, NH_2_, Ar-H), 9.25 (s, 1H, OH)


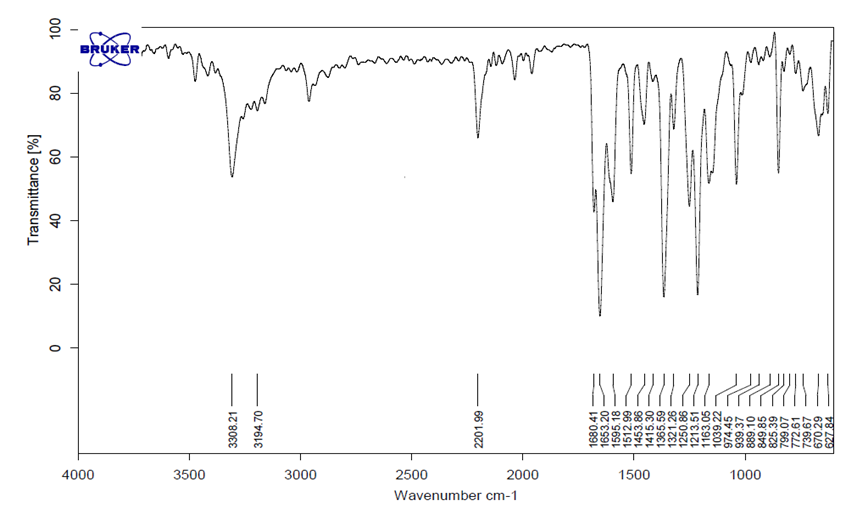


**Fig.S 55. The FT-IR of** **2-Amino-3-cyano-4-(4-hydroxyphenyl)-7,7-dimethyl-5-oxo-4*H*-5,6,7,8-tetrahydrobenzo[*b*]pyran**


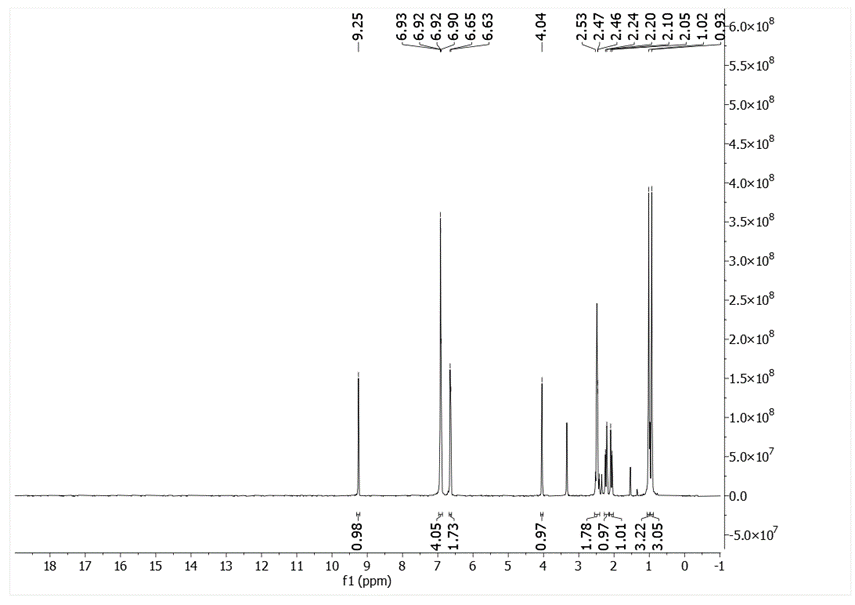


**Fig.S 56. The ^1^H NMR spectrum of 2-Amino-3-cyano-4-(4-hydroxyphenyl)-7,7-dimethyl-5-oxo-4*H*-5,6,7,8-tetrahydrobenzo[*b*]pyran**

**2-Amino-3-cyano-4-(4-methoxyphenyl)-7,7-dimethyl-5-oxo-4*H*-5,6,7,8-tetrahydrobenzo[*b*]pyran**

Pale yellow solid, m.p. 208-210 ºC.

FT-IR (ATR) ῡ ‎(cm^-1^): 3391, 3329, 2191, 1678, 1654, 1214


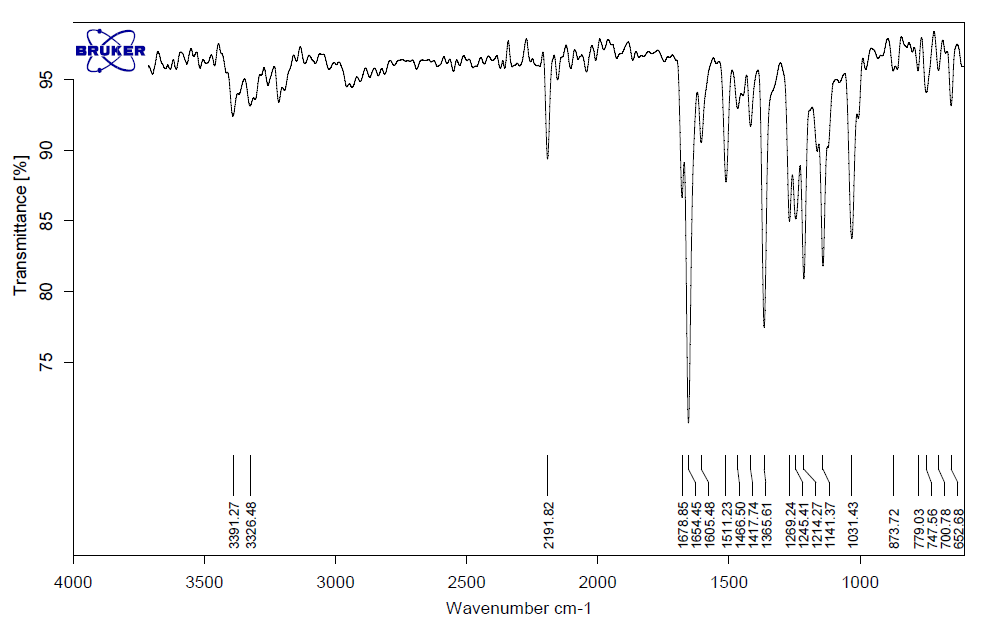


**Fig.S 57. The FT-IR of 2-Amino-3-cyano-4-(4-methoxyphenyl)-7,7-dimethyl-5-oxo-4*H*-5,6,7,8-tetrahydrobenzo[*b*]pyran**

**2-Amino-7,7-dimethyl-4-(3-nitrophenyl)-5-oxo-5,6,7,8-tetrahydrobenzo[*b*]pyran**

Yellow solid, m.p. 216-218 ºC. FT-IR (ATR) ῡ (cm^-1^): 3429, 3332, 2185, 1659, 1637, 1528, 1348, 1208. ^1^H NMR (400 MHz, DMSO-*d_6_*)/ δ ppm: 0.96 (s, 3H), 1.04 (s, 3H), 2.14 (d, *J* = 16.0 Hz, 1H), 2.30 (d, *J* = 16.0 Hz, 1H), 2.50-2.60 (m, 2H), 4.42 (s, 1H,( 7.21 (s, NH_2_, 2H), 7.63 (t, *J* = 8.0 Hz, 1H), 7.68 (d, *J* = 8.0 Hz, 1H), 7.98(s, 1H), 8.08 (d, *J* = 8.0 Hz, 1H). ^13^C NMR(100 MHz, DMSO-*d_6_*)/ δ ppm: 26.69, 28.30, 31.63, 35.35, 49.81, 57.11, 111.72, 119.34, 121.62, 121.77, 130.01, 134.17, 146.98, 147.72, 158.58, 163.15, 195.77


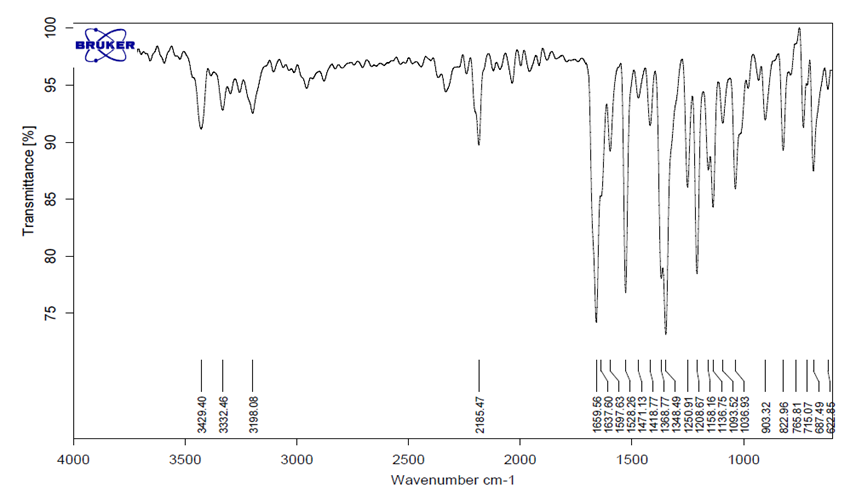


**Fig.S 58. The FT-IR of** **2-Amino-7,7-dimethyl-4-(3-nitrophenyl)-5-oxo-5,6,7,8-tetrahydrobenzo[*b*]pyran**

**Fig.S 59. The ^1^H NMR spectrum of 2-Amino-7,7-dimethyl-4-(3-nitrophenyl)-5-oxo-5,6,7,8-tetrahydrobenzo[*b*]pyran**

**Fig.S 60. The ^1^H NMR spectrum of 2-Amino-7,7-dimethyl-4-(3-nitrophenyl)-5-oxo-5,6,7,8-tetrahydrobenzo[*b*]pyran**

**2-Amino-7,7-dimethyl-4-(2-nitrophenyl)-5-oxo-5,6,7,8-tetrahydrobenzo[*b*]pyran**

Yellow solid, m.p. 234-236 ºC. FT-IR (ATR) ‎ῡ ‎(cm^-1^): 3472, 3300, 2957, 2192, 1686, 1660, 1522, 1356, 1213. ^1^H NMR (400 MHz, DMSO-*d_6_*(/ δ ppm: 0.88 (s, 3H), 1.02 (s, 3H), 2.02 (d, *J* = 16.0 Hz, 1H), 2.21 (d ,*J* = 16.0 Hz, 1H), 2.44-2.57 (m, 2H), 4.94 (s, 1H), 7.22 (s, NH_2_ , 2H), 7.36 (d, *J* = 8.0 Hz , 1H), 7.43 (t, *J* = 8.0 Hz, 1H), 7.66 (t, *J* = 8.0 Hz, 1H), 7.82 (d, *J* = 8.0 Hz, 1H) . ^13^C NMR (100 MHz ,DMSO-*d_6_* )/ δ ppm: 26.64, 28.26, 29.86, 31.82, 49.50, 56.25, 112.28, 119.06, 123.69, 127.84, 130.25, 133.34, 138.93, 148.93, 159.16, 162.69, 195.81 .


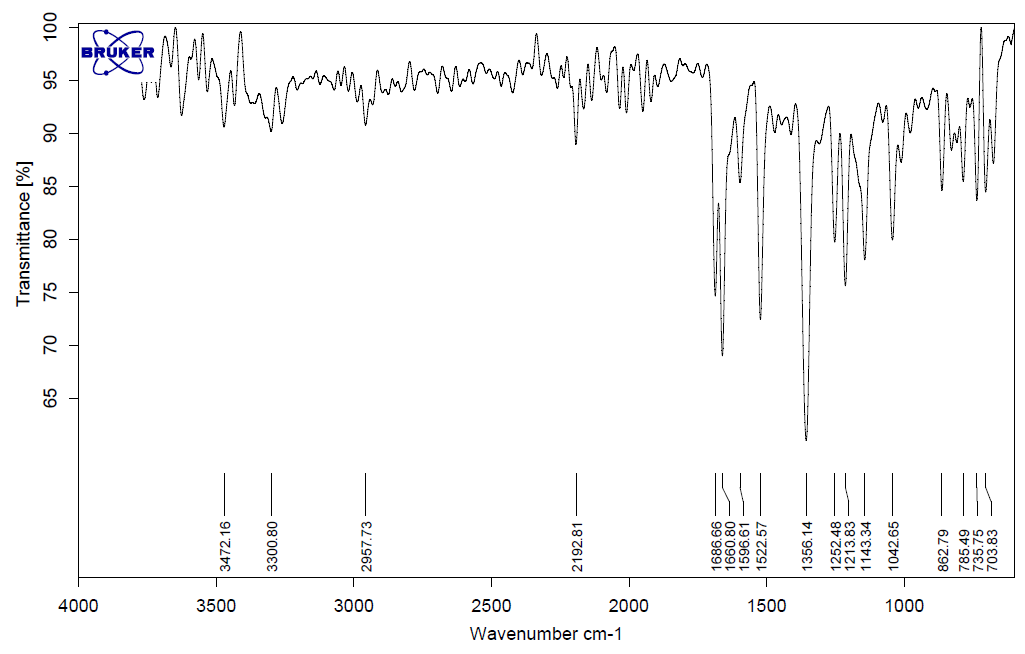


**Fig.S 61. The FT-IR of** **2-Amino-7,7-dimethyl-4-(2-nitrophenyl)-5-oxo-5,6,7,8-tetrahydrobenzo[*b*]pyran**

**Fig.S 62. The ^1^H NMR spectrum of 2-Amino-7,7-dimethyl-4-(2-nitrophenyl)-5-oxo-5,6,7,8-tetrahydrobenzo[*b*]pyran**

**Fig.S 63. The ^1^H NMR spectrum of 2-Amino-7,7-dimethyl-4-(2-nitrophenyl)-5-oxo-5,6,7,8-tetrahydrobenzo[*b*]pyran**

**2-Amino-4-(2-chlorophenyl)-3-cyano-7,7-dimethyl-5-oxo-4*H*-5,6,7,8 tetrahydrobenzo[*b*]pyran**

Pale yellow solid, m.p. 216-218 ºC

FT-IR (ATR) ῡ (cm^-1^): 3388, 3327, 2197, 1653, 1654, 1214

^1^H NMR (400 MHz, DMSO-*d_6_*)/ δ ppm: 0.97 (s, 3H), 1.03 (s, 3H), 2.08 (d, *J* = 16.0 Hz, 1H), 2.25 (d, *J* = 16.0 Hz, 1H), 2.45-2.56 (m, 2H), 4.68 (s, 1H), 7.03 (s, 2 H, NH_2_), 7.15-7.36 (m, 4H)


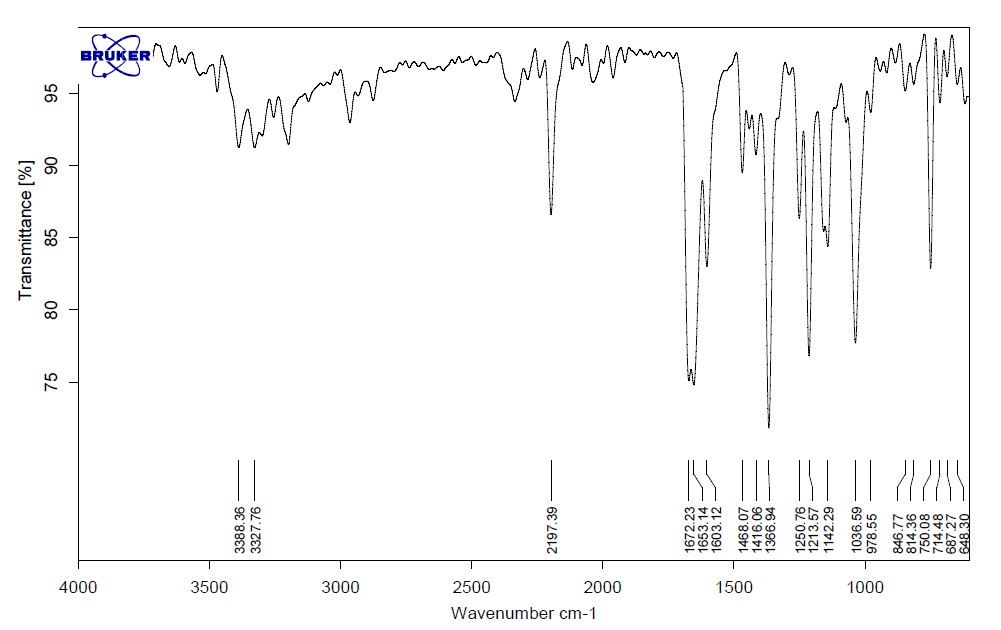


**Fig.S 64. The FT-IR of 2-Amino-4-(2-chlorophenyl)-3-cyano-7,7-dimethyl-5-oxo-4*H*-5,6,7,8 tetrahydrobenzo[*b*]pyran**

**
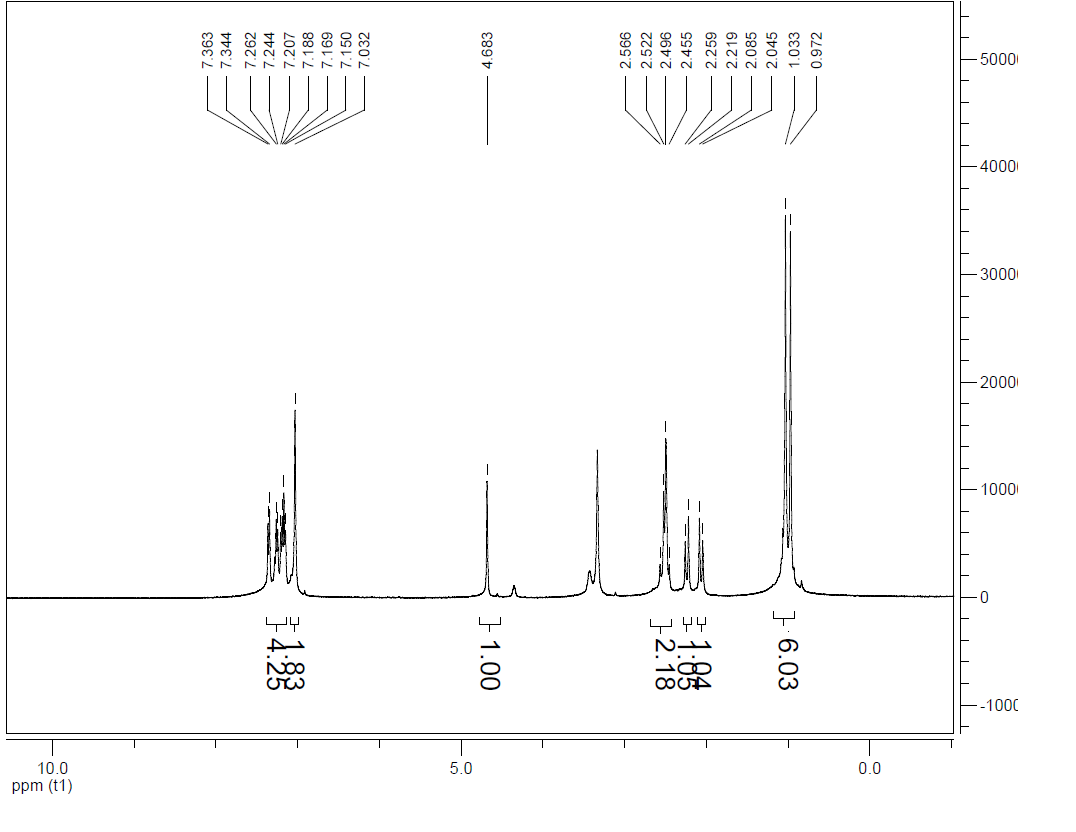
**

**Fig.S 65. The ^1^H NMR spectrum of 2-Amino-4-(2-chlorophenyl)-3-cyano-7,7-dimethyl-5-oxo-4*H*-5,6,7,8 tetrahydrobenzo[*b*]pyran**

**2-Amino-4-(2,6-chlorophenyl)-3-cyano-7,7-dimethyl-5-oxo-4*H*-5,6,7,8tetrahydrobenzo[*b*]pyran**

White solid, m.p. 249-251 ºC FT-IR (ATR) ῡ ‎ (cm^-1^): 3378, 3322, 2187, 1674, 1631, 1214. ^1^H NMR (400 MHz, DMSO-*d_6_*)/ δ ppm: 0.98 (s, 3H), 1.04 (s, 3H), 2.05 (d, *J* = 16.0 Hz, 1H), 2.23 (d *,J* = 16.0 Hz, 1H), 2.38 (d, *J* = 16.0 Hz, 1H), 2.52 (d, *J* =16.0 Hz, 1H), 5.19 (s, 1H,( 7.09 (s, NH_2_, 2H), 7.23 (t ,*J* = 8.0 Hz, 1H), 7.33 (d, *J* = 7.2 Hz, 1H), 7.43 (d, *J* = 6.8 Hz, 1H) ^13^C NMR (100 MHz, DMSO-*d_6_*)/ δ ppm: 28.93, 32.00, 32.70, 50.37, 56.50, 110.41, 128.94, 129.45, 130.67, 134.59, 136.77, 159.91, 164.26, 196.22


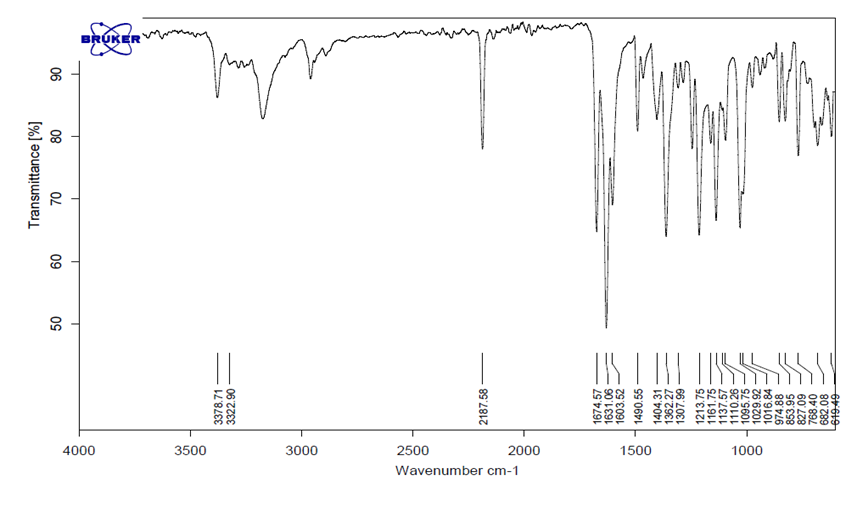


**Fig.S 66. The FT-IR of** **2-Amino-4-(2,6-chlorophenyl)-3-cyano-7,7-dimethyl-5-oxo-4*H*-5,6,7,8tetrahydrobenzo[*b*]pyran**

**Fig.S 67. The ^1^H NMR spectrum of 2-Amino-4-(2,6-chlorophenyl)-3-cyano-7,7-dimethyl-5-oxo-4*H*-5,6,7,8tetrahydrobenzo[*b*]pyran**

**Fig.S 68. The ^1^H NMR spectrum of 2-Amino-4-(2,6-chlorophenyl)-3-cyano-7,7-dimethyl-5-oxo-4*H*-5,6,7,8tetrahydrobenzo[*b*]pyran**

**2-Amino-3-cyano-7,7-dimethyl-5-oxo-4-phenyl-4*H*-5,6,7,8 tetrahydrobenzo[*b*]pyran**

Wite solid, m.p. 231-234 ºC ‎‎ FT-IR (ATR) ῡ (cm^-1^): 3386, 3208, 2198, 1677, 1656, 1212

^1^H NMR (400 MHz, DMSO-*d_6_*)/ δ ppm: 0.94 (s, 3H), 1.02 (s, 3H), 2.10 (d, *J* = 16.0 Hz, 1H), 2.26 (d, *J* = 16.0 Hz, 1H), 2.49-2.55 (m, 2H), 4.15 (s, 1H), 7.00 (s, 2H, NH_2_), 7.11-7.29 (m, 5H)


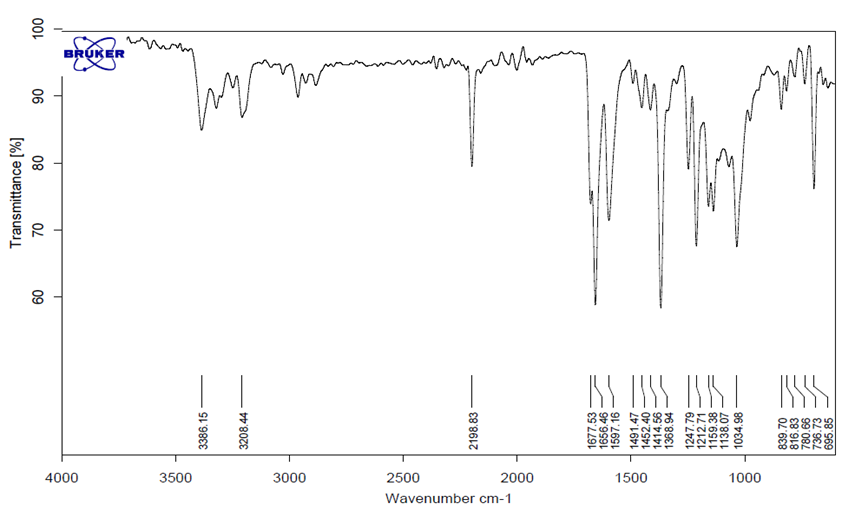


**Fig.S 69. The FT-IR of 2-Amino-3-cyano-7,7-dimethyl-5-oxo-4-phenyl-4*H*-5,6,7,8 tetrahydrobenzo[*b*]pyran**


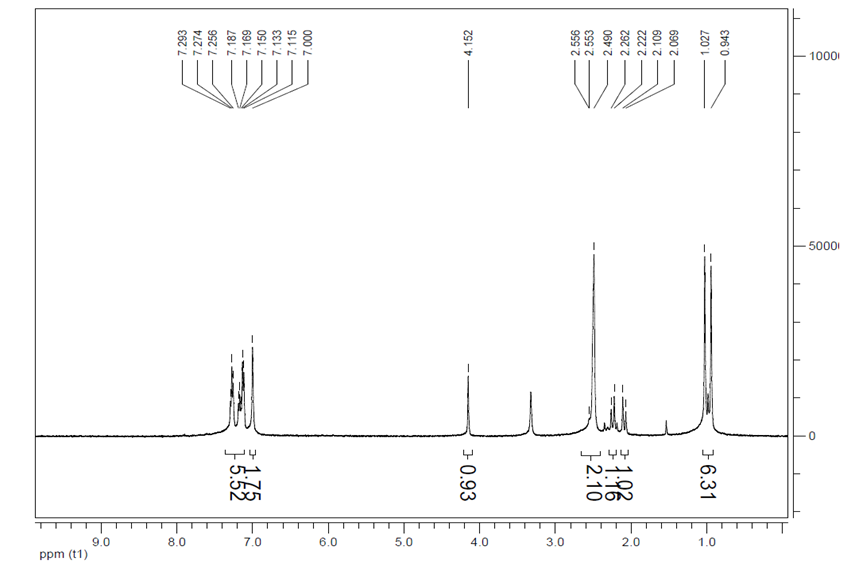


**Fig.S 70. The ^1^H NMR spectrum of 2-Amino-3-cyano-7,7-dimethyl-5-oxo-4-phenyl-4*H*-5,6,7,8 tetrahydrobenzo[*b*]pyran**

**2-Amino-3-cyano-4-(furan-2-yl)-7,7-dimethyl-5-oxo-4*H*-5,6,7,8-tetrahydrobenzo [*b*]pyran**

Cream powder, m.p. 217-219 ºC. FT-IR (ATR) ῡ (cm^-1^): 3385, 3213, 2194, 1654, 1601, 1214. ^1^H NMR (400 MHz, DMSO-*d_6_*)/ δ ppm: 0.99 (s, 3 H), 1.05 (s, 3H), 2.15 (d, *J* = 16.0 Hz, 1H), 2.29 (d, *J* = 16.0 Hz, 1H), 2.43–2.68 (m, 2H), 4.33 (s, 1H), 6.09 (d, *J* = 4.0 Hz, 1H), 6.31 (m, 1H), 7.12 (s, NH_2_, 2H), 7.47 (d, *J* = 4.0 Hz, 1H). ^13^C NMR (100 MHz, DMSO-*d_6_*)/ δ ppm: 26.51, 28.39, 28.93, 31.79, 49.85, 55.31, 105.03, 110.31, 110.42, 119.52, 141.73, 155.67, 159.23, 163.25, 195.42


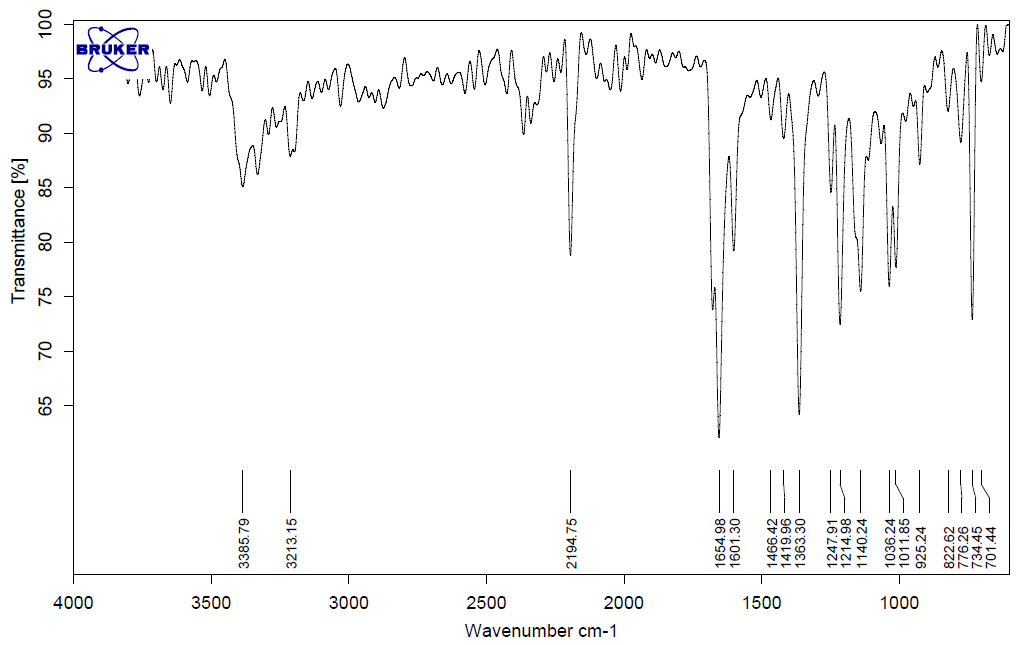


**Fig.S 71. The FT-IR of** **2-Amino-3-cyano-4-(furan-2-yl)-7,7-dimethyl-5-oxo-4*H*-5,6,7,8-tetrahydrobenzo [*b*]pyran**

**Fig.S 72. The ^1^H NMR spectrum of 2-Amino-3-cyano-4-(furan-2-yl)-7,7-dimethyl-5-oxo-4*H*-5,6,7,8-tetrahydrobenzo [*b*]pyran**

**Fig.S 73. The ^1^H NMR spectrum of 2-Amino-3-cyano-4-(furan-2-yl)-7,7-dimethyl-5-oxo-4*H*-5,6,7,8-tetrahydrobenzo [*b*]pyran**

**2-Amino-3-cyano-4-pentyl-7,7-dimethyl-5-oxo-4*H*-5,6,7,8-tetrahydrobenzo[*b*] pyran**

Pale yellow solid, m. p. 162-164 ºC. FT-IR (ATR) ῡ (cm^-1^): 3383 ,3299, 2183, 1678, 1653, 1217.^1^H NMR (400 MHz, CDCl_3_)/ δ ppm: 0.79 (t, *J* = 8.0 Hz, 3H, CH_3_), 1.02-1.04 (2s, 6H, 2CH_3_), 1.11-1.30 (m, 6H, 3CH_2_), 1.40-1.57 (m, 2H ,CH_2_), 2.17-2.37 (m, 4H, 2CH_2_,( 3.33 (t, 1H *,J* = 8.0 Hz), 4.62 (s, NH_2_, 2H). ^13^C NMR (100 MHz, CDCl_3_)/ δ ppm: 14.02, 22.57, 24.52, 27.40, 29.11, 29.32, 31.69, 32.04, 34.67, 40.64, 50.84, 60.76, 113.95, 119.48, 159.01, 162.73, 196.76.


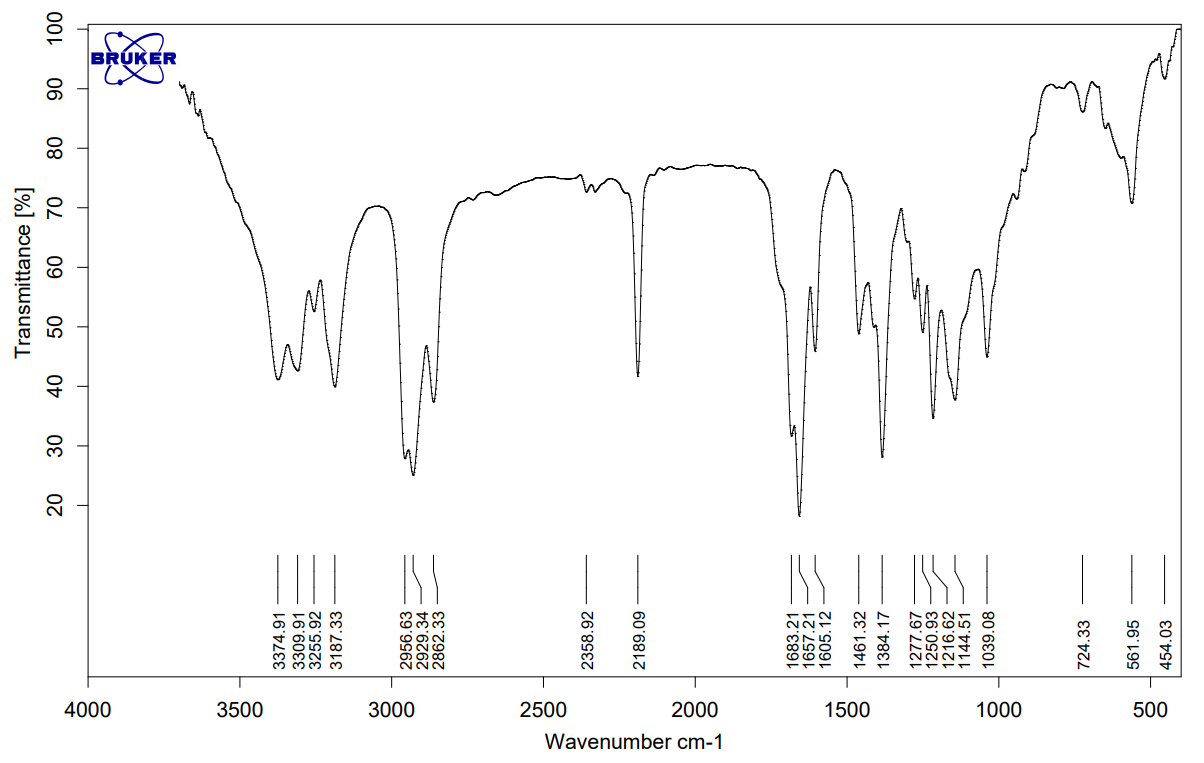


**Fig.S 74. The FT-IR of 2-Amino-3-cyano-4-pentyl-7,7-dimethyl-5-oxo-4*H*-5,6,7,8-tetrahydrobenzo[*b*] pyran**


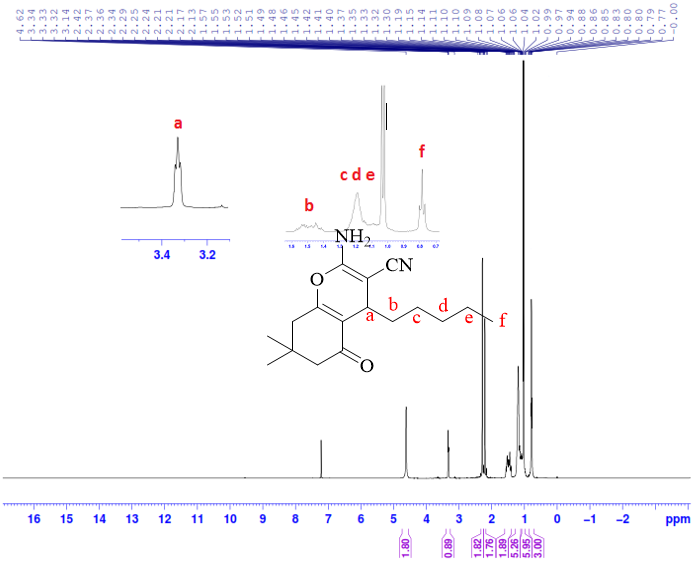


**Fig.S 75. The ^1^H NMR spectrum of 2-Amino-3-cyano-4-pentyl-7,7-dimethyl-5-oxo-4*H*-5,6,7,8-tetrahydrobenzo[*b*] pyran**


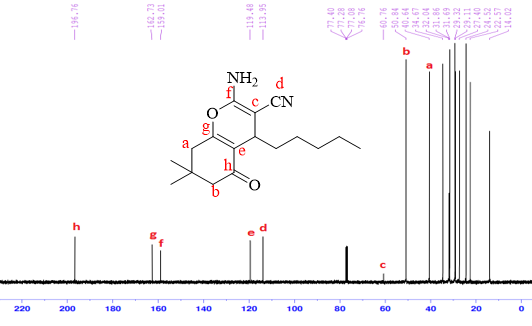


**Fig.S 76. The ^13^C NMR spectrum of** **2-Amino-3-cyano-4-pentyl-7,7-dimethyl-5-oxo-4*H*-5,6,7,8-tetrahydrobenzo[*b*] pyran**

**(E)-2-amino-3-cyano-7,7-dimethyl-5-oxo-4-styryl-4H-5,6,7,8-tetrahydrobenzo [*b*]pyran**

Pale yellow solid, m. p. 217-219ºC. FT-IR (ATR) ῡ (cm^-1^): 3383, 3292, 2181, 1680, 1649, 1215. ^1^H NMR (400 MHz, DMSO-d_6_)/ δ ppm: 1.01 (s, 3H), 1.04 (s, 3H), 2.21 (d, *J* = 16.0 Hz, 1H), 2.29 (d ,*J* = 16.0 Hz, 1H), 2.39-2.50 (m, 2H, CH_2_), 3.82 (d *,J* = 8.0 Hz, 1H), 6.08 (dd, *J_1_* = 16.0 Hz, *J_2_* = 8.0 Hz, 1H) ,6.37 (d ,*J* = 16.0 Hz, 1H) 7.08(s, NH_2_, 2H), 7.23 (t ,*J* = 8.0 Hz, 1H), 7.31 (t, *J* = 8.0 Hz, 2H), 7.39 (d, *J* = 8.0 Hz, 2H). ^13^C NMR (100 MHz, DMSO-*d_6_* )/ δ ppm: 26.89, 28.18, 31.81, 32.77, 50.05, 55.10, 111.82, 119.87, 126.19, 127.40, 128.31, 129.23, 131.07, 136.43, 159.16, 162.39, 195.90.


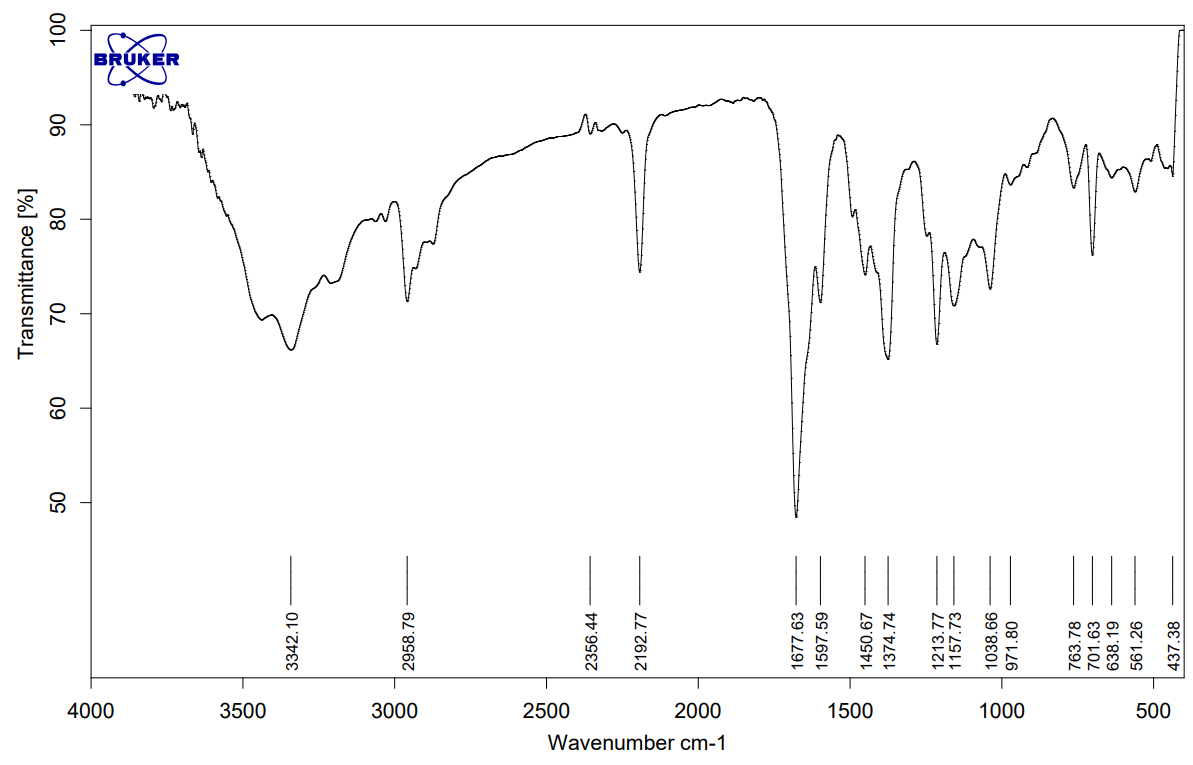


**Fig.S 77. The FT-IR of (E)-2-amino-3-cyano-7,7-dimethyl-5-oxo-4-styryl-4H-5,6,7,8-tetrahydrobenzo [*b*]pyran**


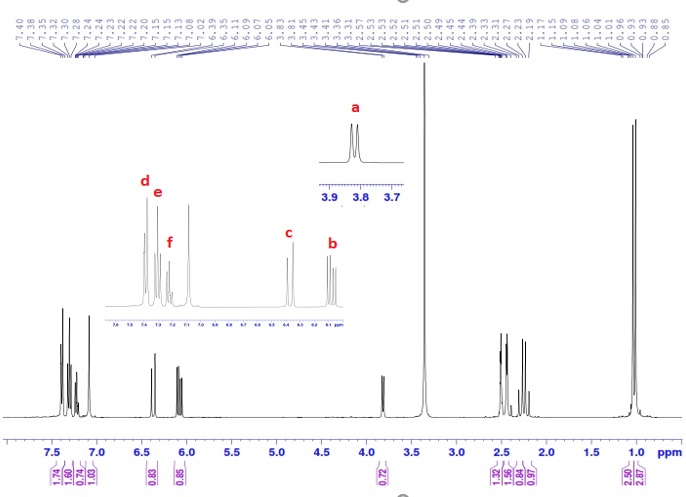


**Fig.S 78. The ^1^H NMR spectrum of (E)-2-amino-3-cyano-7,7-dimethyl-5-oxo-4-styryl-4H-5,6,7,8-tetrahydrobenzo [*b*]pyran**


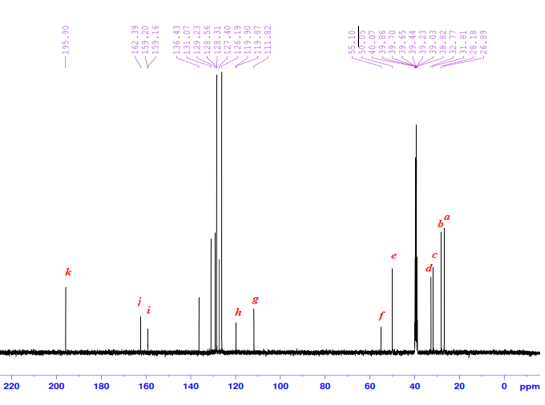


**Fig.S 79. The ^13^C NMR spectrum of (E)-2-amino-3-cyano-7,7-dimethyl-5-oxo-4-styryl-4H-5,6,7,8-tetrahydrobenzo [*b*]pyran**
